# Bridging the Energy Balance Gap in Eddy‐Covariance Measurements: Insights From Standardized Network Data

**DOI:** 10.1111/gcb.70892

**Published:** 2026-05-04

**Authors:** Giacomo Nicolini, David Durden, Luca Di Fiore, Christopher Florian, Simone Sabbatini, Bert Gielen, Arne Iserbyt, Benjamin Loubet, Ivan Mammarella, Adriana Mariotti, Maarten Op de Beeck, Caleb Slemmons, Carlo Trotta, Adam Young, Abad Chabbi, Iris Feigenwinter, Bernard Heinesch, Natalia Kowalska, Matthias Mauder, Ladislav Šigut, Michiel van der Molen, Flavio Bastos Campos, Daniel Berveiller, Christian Brümmer, Matthias Cuntz, Jean‐Christophe Domec, Benjamin Dumont, Silvano Fares, Damiano Gianelle, Rasmus Jensen, Carmen Kalalian, Natascha Kljun, Holger Lange, Jean‐Marc Limousin, Erik Lundin, Antonio Manco, Leonardo Montagnani, Eiko Nemitz, Matthias Peichl, Erkka Rinne, Marilyn Roland, Marius Schmidt, Guillaume Simioni, Abin Thomas, Caroline Vincke, Dario Papale

**Affiliations:** ^1^ CMCC Foundation‐Euro‐Mediterranean Center on Climate Change‐IAFES Division Viterbo Italy; ^2^ National Ecological Observatory Network Battelle Boulder Colorado USA; ^3^ Plants and Ecosystems (PLECO), Department of Biology University of Antwerp Antwerp Belgium; ^4^ UMR ECOSYS, Ecologie Fonctionnelle et Écotoxicologie des Agroécosystèmes Université Paris‐Saclay, INRAE, AgroParisTech Paris France; ^5^ Institute for Atmospheric and Earth System Research/Physics, Faculty of Science University of Helsinki Helsinki Finland; ^6^ URP3F, Pluridisciplinaire Prairies et Plantes Fourragères, Centre de Recherche INRAE Lusignan France; ^7^ Institute of Agricultural Sciences ETH Zurich Zurich Switzerland; ^8^ TERRA Teaching and Research Center, Gembloux Agro‐Bio Tech University of Liege Liege Belgium; ^9^ Department of Matters and Energy Fluxes Global Change Research Institute of the Czech Academy of Sciences Brno Czech Republic; ^10^ Institute of Hydrology and Meteorology Dresden University of Technology Dresden Germany; ^11^ Meteorology and Air Quality Group Wageningen University Wageningen the Netherlands; ^12^ Helmholtz‐Centre for Environmental Research Leipzig Germany; ^13^ Ecologie Société Evolution, CNRS, AgroParisTech Université Paris‐Saclay Paris France; ^14^ Thünen Institute of Climate‐Smart Agriculture Braunschweig Germany; ^15^ Université de Lorraine, AgroParisTech, INRAE, UMR Silva Nancy France; ^16^ Bordeaux Sciences Agro UMR ISPA INRAE Bordeaux France; ^17^ Department of Biology William and Mary Williamsburg Virginia USA; ^18^ Nicholas School of the Environment Duke University Durham North Carolina USA; ^19^ Gembloux Agro‐Bio Tech University of Liege Gembloux Belgium; ^20^ National Research Council of Italy Rome Italy; ^21^ Fondazione E. Mach San Michele all'Adige Italy; ^22^ Department of Ecoscience Aarhus University Aarhus Denmark; ^23^ Department of Earth and Environmental Sciences Lund University Lund Sweden; ^24^ Norwegian Institute of Bioeconomy Research Ås Norway; ^25^ CEFE, Univ Montpellier, CNRS, EPHE, IRD Montpellier France; ^26^ Abisko Scientific Research Station Swedish Polar Research Secretariat Abisko Sweden; ^27^ Institute for Agriculture and Forestry Systems in the Mediterranean (ISAFoM) National Research Council of Italy Naples Italy; ^28^ Faculty of Agricultural, Environmental and Food Sciences Free University of Bolzano Bolzano Italy; ^29^ UK Centre for Ecology & Hydrology Penicuik UK; ^30^ Department of Forest Ecology and Management Swedish University of Agricultural Sciences Umeå Sweden; ^31^ Climate System Research Finnish Meteorological Institute Helsinki Finland; ^32^ Institute of Bio‐ and Geosciences: Agrosphere (IBG‐3) Jülich Germany; ^33^ Ecologie des Forêt Méditerranéennes INRAE‐URFM Avignon France; ^34^ Earth and Life Institute, Environmental Sciences Université Catholique de Louvain (UCLouvain) Louvain‐la‐Neuve Belgium; ^35^ Department for Innovation in Biological, Agro‐Food and Forest Systems (DIBAF) University of Tuscia Viterbo Italy

**Keywords:** eddy covariance, energy balance closure, FLUXNET, ICOS, NEON, standardization

## Abstract

The lack of energy balance closure in Eddy‐Covariance (EC) measurements is a well‐known, still unresolved challenge in micrometeorology, with energy balance closure (EBC) rates typically ranging between 60% and 80%. While numerous hypotheses have been proposed to explain this imbalance, the relative contributions of neglected energy storage terms, data quality and flux processing options remain insufficiently disentangled. Using standardized ICOS and NEON datasets, we show that a significant portion of the observed energy imbalance can be attributed to overlooked or inconsistently handled energy components and turbulent flux quality control. Using data drawn from 84 sites, we show that comprehensive energy accounting—including soil heat flux, storage terms (soil, air, biomass), photosynthetic energy demand, and strict quality filtering of turbulent fluxes—improved EBC by 16% on average, with site‐specific gains up to 40%. However, we also identify a persistent residual imbalance that is unlikely to be resolved through methodological refinements or additional measurements alone, pointing to fundamental physical processes that are not accounted for in the standard measurement and processing. We argue that this unresolved imbalance should be explicitly acknowledged and bounded, rather than implicitly absorbed into correction schemes, and we outline practical guidance for diagnosing and interpreting EBC in standardized flux networks. This perspective evaluates methodological advances and residual uncertainties, providing an actionable framework for the appropriate use of EC energy fluxes in carbon, water, and climate research.

## Graphical abstract

Written Summary: Measuring energy exchanges between land and atmosphere is fundamental to understanding climate, but these measurements are systematically incomplete, with 20%–40% of energy consistently unaccounted for. Analyzing data from 84 sites worldwide, we show that carefully tracking all energy components and applying strict data quality filters recovers a significant portion of this missing energy. However, a residual gap remains that cannot be closed with current methods, and we provide practical guidance for scientists to properly account for this limitation in climate and ecosystem research.

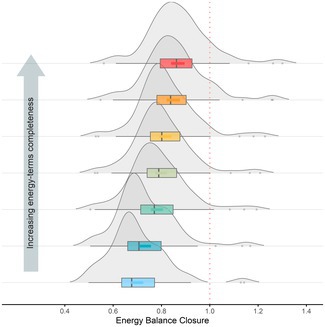

## 1 Introduction

The accurate quantification of turbulent exchange processes between terrestrial ecosystems and the atmosphere is fundamental to understanding global biogeochemical cycles, hydrological budgets, and climate dynamics. The eddy covariance (EC) method is the most widely adopted micrometeorological technique for directly measuring these fluxes, providing invaluable insights into ecosystem‐scale carbon, water, and energy transfers across diverse biomes worldwide (Baldocchi 2020). EC systems are critical tools in validating remote sensing products, informing Earth system models, and monitoring the impacts of environmental change, as well as in providing observations to policymakers for evidence‐based decisions.

Despite this central role, incomplete energy balance closure (EBC) remains an ongoing challenge that limits both the interpretation and credibility of EC‐derived fluxes (e.g., Mauder et al. 2020). Numerous studies across various ecosystems consistently report that the sum of measured turbulent fluxes (sensible and latent heat) typically accounts for only 60%–80% of the available energy (net radiation minus heat stored in the ground, the air column and biomass below the measurement point), with closure rates at half‐hour resolution occasionally dropping as low as 40% (Wilson et al. 2002; Foken 2008; Mauder et al. 2020; Stoy et al. 2013). This consistent shortfall, observed over decades, suggests a systemic bias rather than mere random measurement errors, indicating significant uncertainties in our understanding of surface energy partitioning. In addition to affecting the quality of heat flux measurements directly, many of the potential causes for nonclosure of the energy balance could also affect the quality of flux measurements of CO_2_, H_2_O and other compounds (Mauder et al. 2007, 2010).

The root causes of energy balance nonclosure continue to fuel extensive debate within the micrometeorological community. Proposed explanations can be broadly categorized into physical and technical factors. Among physical causes, the unaccounted contribution of mesoscale atmospheric motions, organized circulations that transport energy beyond single‐tower detection scales, is now regarded as the principal explanation for the persistent energy imbalance (see Foken et al. 2009, 2012; Gao et al. 2017; Mauder et al. 2024). Additional physical factors include surface heterogeneity‐driven motions (Butterworth et al. 2024; Moderow et al. 2009; Stoy et al. 2013), unaccounted energy in biomass, air, and soil layers not fully captured by standard EC measurements (Leuning et al. 2012; Meyers and Hollinger 2004), and spatial heterogeneity in surface characteristics (Steinfeld et al. 2006). Several studies indicate that large coherent eddies can disrupt the classical scalar surface layer under convective conditions, resulting in asymmetric transport and systematic flux underestimation, and thereby contributing to energy balance nonclosure, especially over heterogeneous landscapes (Liu et al. 2021, 2025).

On the technical side, instrumental limitations, such as inadequate frequency response corrections (Massman and Lee 2002; Ibrom et al. 2007), limitations in the sensors like the transducers shadowing in the sonic anemometers (Frank et al. 2016), and uncertainties in data‐processing schemes (Frank et al. 2013; Mauder et al. 2013) have also been implicated. While these factors undoubtedly contribute to the imbalance, the consistent and often substantial magnitude of the discrepancy suggests a more complex interplay of contributing mechanisms, with no single, universally accepted explanation emerging.

A critical, yet often underexplored, factor contributing to persistent energy balance nonclosure is the foundational quality of the input data itself. Incomplete measurements of the relevant terms or suboptimal data processing options can propagate systematic biases throughout the flux calculation chain, confounding the interpretation of the mechanisms leading to the imbalance. Addressing these fundamental data quality aspects could provide crucial clarity regarding the true magnitude of the unexplained imbalance and guide more targeted future research efforts.

Global networks like FLUXNET have been instrumental in synthesizing EC data from hundreds of sites worldwide, enabling unprecedented large‐scale analyses of ecosystem processes (Pastorello et al. 2020). However, the inherent heterogeneity in site‐specific protocols, instrumentation, and data processing methods across FLUXNET contributes to uncertainty and complicates direct cross‐site comparisons of energy balance components (Mauder et al. 2024). In contrast, standardized observation networks, such as Europe's Integrated Carbon Observation System (ICOS, Heiskanen et al. 2022) and the U.S. National Ecological Observatory Network (NEON, www.neonscience.org), enforce stringent and uniform data collection protocols, standardized quality assurance/quality control (QA/QC) procedures, and comprehensive monitoring of the relevant energy balance components, including often‐overlooked terms like soil and air heat storage (Lindroth et al. 2010). This approach offers a unique opportunity to minimize measurement‐related uncertainties and isolate the physical or theoretical gaps contributing to non‐closure.

This study leverages the high‐quality, standardized datasets from ICOS and NEON to dissect the ecosystem‐specific contributions of individual energy balance terms. By systematically analyzing these comprehensive datasets, we aim to (1) identify the dominant factors leading to non‐closure across a range of ecosystems at different temporal scales, (2) differentiate between measurement‐related uncertainties and actual physical or theoretical gaps, and (3) improve the quantitative characterization of the unresolved energy imbalance. We do not aim to propose a correction for energy balance closure. Instead, we synthesize evidence from standardized network data to identify which components of the imbalance can be attributed to methodological omissions and delineate the residual imbalance. The insights gained will serve to guide future methodological improvements in EC measurements and to advance our understanding of surface energy partitioning.

## 2 Materials and Methods

### 2.1 Data Collection

In this study, we use data and metadata from the ICOS Level 2 (L2) collection covering the period up to December 2024: “Ecosystem final quality (L2) product in ETC‐Archive format—release 2025‐1”, DOI: https://doi.org/10.18160/S6HM‐CP8Q (ICOS RI et al. 2025). NEON eddy covariance data (DP4.00200.001; NEON (47) (2025)) were gathered from the AmeriFlux API, and the AmeriFlux BASE product was formatted similarly to the ICOS Level 2 collection using a custom workflow to provide additional variables not available from AmeriFlux (Chu et al. 2023). A total of 84 stations have been included covering a wide range of terrestrial ecosystems: 38 ICOS stations and 46 NEON stations (Table 1, station map and further details on vegetation cover in the Supporting Information). The individual station data span from 1 to 7 years. The two networks are both characterized by centralized data processing, a richness of measurements and metadata, and an open data sharing policy. However, they also differ in three main technical choices: (1) the sonic anemometer model deployed, the Gill HS‐50/100 at ICOS stations and the Campbell CSAT3 at NEON stations; (2) the setup of the gas analyzer, both using the LI‐COR LI7200, but with a different intake tube and rain cup; and (3) the raw data processing pipeline (implementation, corrections and their order).

**TABLE 1:** Stations used in the study.

Station	Latitude	Longitude	elev.	PFT	EC h	ST	Slope	asp.	can. h	biom.	References
	°.d	°.d	m		m		°		m	kg m^−2^	
BE‐Bra	51.30761	4.51984	16	ENF	32.6	Y	0.31	W	13.1	22.0	Janssens et al. (2025)
BE‐Dor	50.31188	4.96811	253	GRA	2.0	N	0.94	ESE	0.2	0.2	Heinesch et al. (2025)
BE‐Lon	50.55162	4.74623	170	CRO	2.0	N	0.07	WNW	0.3	1.1	Dumont et al. (2025)
BE‐Maa	50.97987	5.63185	87	CSH	3.0	N	0.38	SE	1.1	1.9	Roland et al. (2025)
BE‐Vie	50.30496	5.99810	490	MF	51.0	Y	3.44	NNW	29.0	58.0	Vincke et al. (2025)
CH‐Dav	46.81533	9.85591	1637	ENF	35.0	Y	16.21	WNW	19.2	62.5	Feigenwinter et al. (2025)
CZ‐BK1	49.50208	18.53688	881	ENF	25.0	Y	5.12	N	17.2	21.4	Šigut et al. (2025)
CZ‐Lnz	48.68155	16.94633	182	DBF	44.0	Y	0.12	W	27.0	76.6	Kowalska et al. (2025)
DE‐Geb	51.09973	10.91463	163	CRO	3.0	N	0.22	ENE	0.5	2.7	Brümmer et al. (2025)
DE‐HoH	52.08656	11.22235	217	DBF	45.0	Y	0.26	SSE	29.5	54.8	Rebmann et al. (2025)
DE‐RuS	50.86591	6.44714	106	CRO	2.7	N	0.55	W	0.4	2.2	Schmidt et al. (2025)
DE‐Tha	50.96256	13.56515	380	ENF	42.0	Y	1.78	E	30.6	41.1	Bernhofer et al. (2025)
DK‐Sor	55.48587	11.64464	55	DBF	43.6	Y	0.22	WSW	23.5	61.1	Ibrom et al. (2025)
FI‐Hyy	61.84741	24.29477	181	ENF	27.0	Y	1.27	SW	21.4	19.2	Mammarella et al. (2025)
FI‐Sii	61.83265	24.19285	164	WET	3.0	N	0.26	NW		0.2	Tuittila et al. (2025)
FI‐Sod	67.36239	26.63859	187	ENF	25.1	N	0.72	WSW	12.4	10.5	Aurela et al. (2025)
FR‐Bil	44.49365	−0.95609	39	ENF	15.6	Y	0.32	NNW	9.5	8.7	Domec et al. (2025)
FR‐FBn	43.24079	5.67865	436	ENF	17.4	Y	2.95	NW	8.4	16.5	Simioni et al. (2025)
FR‐Fon	48.47636	2.78010	103	DBF	37.0	Y	2.39	W	22.2	50.0	Berveiller et al. (2025)
FR‐Gri	48.84422	1.95191	125	CRO	2.0	N	0.86	WNW	0.3	4.2	Buysse et al. (2025)
FR‐Hes	48.67410	7.06465	310	DBF	30.0	Y	1.71	NNE	16.4	42.6	Cuntz et al. (2025)
FR‐Lqu	45.64440	2.73490	1040	GRA	2.0	N	5.75	W	0.1	0.2	Klumpp et al. (2025)
FR‐Lus	46.41425	0.12065	154	GRA	2.0	N	0.36	NE	0.2	0.2	Gastal et al. (2025)
FR‐Pue	43.74130	3.59570	271	EBF	12.0	Y	5.76	NNW	5.2	20.1	Limousin et al. (2025)
GL‐ZaF	74.48152	−20.55577	42	WET	3.2	N	1.22	S	0.2	0.3	Jackowicz‐Korczynski et al. (2025)
IT‐BCi	40.52375	14.95744	10	CRO	2.1	N	0.27	WSW	0.8	2.3	Magliulo et al. (2025)
IT‐Cp2	41.70427	12.35729	17	MF	20.9	N	0.08	S	13.8	33.0	Fares et al. (2025)
IT‐MBo	46.01468	11.04583	1550	GRA	2.4	N	4.71	S	0.1	0.4	Gianelle et al. (2025)
IT‐Ren	46.58686	11.43369	1744	ENF	33.7	Y	9.57	S	15.1	38.8	Montagnani et al. (2025)
IT‐SR2	43.73202	10.29091	4	ENF	24.3	Y	0.07	NW	20.3	32.0	Arriga et al. (2025)
NL‐Loo	52.16645	5.74355	33	ENF	38.2	Y	0.24	SW	16.4	24.9	van der Molen et al. (2025)
NO‐Hur	60.37163	11.07949	275	ENF	42.0	Y	2.54	W	17.6	20.4	Lange et al. (2025)
SE‐Deg	64.18203	19.55654	270	WET	3.0	Y	0.66	NNW	0.4	0.2	Nilsson et al. (2025)
SE‐Htm	56.09763	13.41897	115	ENF	27.0	Y	1.01	S	19.3	31.5	Heliasz et al. (2025)
SE‐Nor	60.08650	17.47950	58	ENF	36.0	Y	0.50	NE	26.3	42.0	Kljun et al. (2025)
SE‐Sto	68.35594	19.04521	352	WET	2.2	N	0.57	ENE	0.3	0.2	Lundin et al. (2025)
SE‐Svb	64.25611	19.77450	267	ENF	34.5	Y	2.17	ESE	14.7	29.0	Peichl et al. (2025)
UK‐AMo	55.79255	−3.24369	268	WET	4.0	N	0.73	NNE	0.6	0.5	Nemitz et al. (2025)
PR‐xGU	17.96955	−66.86870	125	EBF	23.0	Y	3.22	SSE	10.0	13.3	NEON (1) (2025)
PR‐xLA	18.02126	−67.07689	16	GRA	8.0	Y	0.08	N	0.4	0.6	NEON (2) (2025)
US‐xAB	45.76244	−122.33032	365	ENF	19.0	Y	2.40	N	34.0	15.6	NEON (3) (2025)
US‐xAE	35.41060	−99.05878	519	GRA	8.0	Y	0.36	SSE	1.0	0.5	NEON (4) (2025)
US‐xBA	71.28241	−156.61936	4	WET	9.0	Y	0.10	W	0.3	0.1	NEON (5) (2025)
US‐xBL	39.03370	−78.04179	183	DBF	8.0	Y	0.47	WSW	1.0	13.1	NEON (6) (2025)
US‐xBN	65.15401	−147.50258	230	ENF	19.0	Y	3.95	ENE	8.0	12.8	NEON (7) (2025)
US‐xBR	44.06389	−71.28738	274	DBF	35.0	Y	2.53	N	23.0	45.1	NEON (8) (2025)
US‐xCL	33.40123	−97.57000	272	GRA	22.0	Y	1.14	NE	13.0	11.0	NEON (9) (2025)
US‐xCP	40.81554	−104.74559	1654	GRA	9.0	Y	0.09	NNW	0.4	0.2	NEON (10) (2025)
US‐xDC	47.16165	−99.10656	575	GRA	8.0	Y	0.59	NNW	0.4	0.6	NEON (11) (2025)
US‐xDJ	63.88112	−145.75136	517	ENF	22.0	Y	2.15	NE	10.0	6.6	NEON (12) (2025)
US‐xDL	32.54173	−87.80388	25	MF	42.0	Y	0.18	NE	30.0	30.9	NEON (13) (2025)
US‐xDS	28.12505	−81.43619	20	CRO	8.0	Y	0.04	W	1.5	0.5	NEON (14) (2025)
US‐xGR	35.68896	−83.50195	575	DBF	45.0	Y	8.57	W	30.0	53.5	NEON (15) (2025)
US‐xHA	42.53691	−72.17265	348	DBF	39.0	Y	2.54	SE	26.0	41.4	NEON (16) (2025)
US‐xHE	63.87580	−149.21335	677	OSH	9.0	Y	2.53	NE	0.3	0.9	NEON (17) (2025)
US‐xJE	31.19484	−84.46862	47	ENF	42.0	Y	0.34	WNW	27.0	17.4	NEON (18) (2025)
US‐xJR	32.59069	−106.84254	1324	OSH	8.0	Y	0.52	NE	0.4	0.1	NEON (19) (2025)
US‐xKA	39.11045	−96.61294	323	GRA	8.0	Y	1.67	N		1.4	NEON (20) (2025)
US‐xKZ	39.10077	−96.56308	414	GRA	8.0	Y	2.05	NNE	1.5	1.9	NEON (21) (2025)
US‐xLE	31.85386	−88.16118	13	MF	47.0	Y	0.33	SE	35.0	48.5	NEON (22) (2025)
US‐xMB	38.24828	−109.38827	1799	OSH	8.0	Y	1.19	WNW	0.2	0.1	NEON (23) (2025)
US‐xML	37.37831	−80.52485	1170	DBF	29.0	Y	2.36	N	18.0	38.7	NEON (24) (2025)
US‐xNG	46.76972	−100.91535	589	GRA	8.0	Y	0.64	NNW	0.4	0.4	NEON (25) (2025)
US‐xNQ	40.17760	−112.45245	1662	OSH	8.0	Y	1.52	ESE	1.2	0.5	NEON (26) (2025)
US‐xNW	40.05425	−105.58237	3490	ENF	8.0	Y	6.50	S	0.2	15.9	NEON (27) (2025)
US‐xRM	40.27590	−105.54596	2742	ENF	25.0	Y	2.44	E	19.0	27.3	NEON (28) (2025)
US‐xRN	35.96413	−84.28259	344	DBF	39.0	Y	0.84	NW	28.0	59.5	NEON (29) (2025)
US‐xSB	29.68928	−81.99343	46	ENF	35.0	Y	0.82	SSE	23.0	8.5	NEON (30) (2025)
US‐xSC	38.89293	−78.13949	352	DBF	52.0	Y	2.73	NNW	35.0	50.5	NEON (31) (2025)
US‐xSE	38.89013	−76.56001	33	DBF	62.0	Y	0.69	SW	38.0	61.9	NEON (32) (2025)
US‐xSJ	37.10878	−119.73228	400	SAV	39.0	Y	2.40	SW	21.0	4.5	NEON (33) (2025)
US‐xSL	40.46189	−103.02929	1365	CRO	8.0	Y	0.22	NNE		0.7	NEON (34) (2025)
US‐xSP	37.03337	−119.26219	1210	ENF	52.0	Y	5.49	E	32.0	3.1	NEON (35) (2025)
US‐xSR	31.91068	−110.83549	997	OSH	8.0	Y	1.50	NW	2.0	0.2	NEON (36) (2025)
US‐xST	45.50894	−89.58637	476	DBF	22.0	Y	0.51	SW	20.0	13.4	NEON (37) (2025)
US‐xTA	32.95047	−87.39326	166	ENF	35.0	Y	0.50	S	25.0	26.4	NEON (38) (2025)
US‐xTE	37.00583	−119.00602	2149	ENF	59.0	Y	4.15	S	35.0	43.1	NEON (39) (2025)
US‐xTL	68.66109	−149.37047	832	WET	9.0	Y	0.50	WNW	0.3	0.1	NEON (40) (2025)
US‐xTR	45.49369	−89.58571	467	DBF	36.0	Y	0.58	WSW	23.0	31.4	NEON (41) (2025)
US‐xUK	39.04043	−95.19215	322	DBF	35.0	Y	1.39	S	19.0	39.3	NEON (42) (2025)
US‐xUN	46.23391	−89.53725	521	MF	39.0	Y	0.91	ESE	24.0	30.3	NEON (43) (2025)
US‐xWD	47.12820	−99.24133	591	GRA	8.0	Y	0.78	NE	1.0	0.5	NEON (44) (2025)
US‐xWR	45.82049	−121.95191	351	ENF	74.0	Y	2.51	E	50.0	69.3	NEON (45) (2025)
US‐xYE	44.95348	−110.53914	2133	ENF	18.0	Y	2.78	N	14.0	23.7	NEON (46) (2025)

*Note:* In addition to station official ID and coordinates are reported: Elevation (elev., m), plant functional type (PFT), height of the eddy covariance system (EC h, m), whether the station is equipped with a storage profile system (ST, yes or no), mean slope at the site (slope, degrees °), site aspect (asp.), mean canopy height (can_h, m), mean aboveground wet biomass (biom., kg m^−2^), and official station/dataset reference. PFT acronyms: CRO, Croplands; CSH, Closed Shrublands; DBF, Deciduous Broadleaf Forests: EBF, Evergreen Broadleaf Forests; ENF, Evergreen Needleleaf Forests; GRA, Grasslands; MF, Mixed Forests; OSH, Open Shrublands; SAV, Savannas; WET, Permanent Wetlands. Further details on fractional vegetation cover and green area index are reported in the Supporting Information.

For ecosystem‐level analyses, underrepresented plant functional types were aggregated into broader categories to improve sample size and statistical power. Groupings were based on structural and functional affinities: closed shrublands, open shrublands, and savannas were combined into a single category (SHR|SAV), while evergreen broadleaf forests were grouped with deciduous broadleaf forests (DBF|EBF).

In addition to the basic site metadata collected from the network databases (e.g., coordinates and heights of the EC systems, ecosystem type), to be consistent across networks elevation, exposure and slope data were estimated using a custom algorithm implemented in Google Earth Engine (GEE, Gorelick et al. 2017). The algorithm queries multiple digital elevation model (DEM) products in hierarchical order based on data availability and spatial coverage: CGIAR/SRTM90_V4 (90 m resolution, 60° N to 56° S latitude, Reuter et al. 2007), NASA/ASTER_GED/AG100_003 (100 m resolution, global coverage excluding Greenland, Hulley et al. 2015), OSU/GIMP/DEM (30 m resolution, Greenland only, Howat et al. 2014), and USGS/GMTED2010_FULL (231.92 m resolution, global coverage, Danielson and Gesch 2011). For each site location, the algorithm extracts elevation values from a 2 × 2 pixel window centered on the site coordinates using the first available DEM in the hierarchy. Topographic metrics (slope and aspect) are then computed using standard finite‐difference methods. Biomass data for the ICOS stations were collected from the ancillary data distributed with the flux data (ICOS RI et al. 2025 and references in Table 1), extracting the mean total aboveground dry biomass measured during inventory campaigns. This allowed having dynamic biomass values over the whole data span. A similar method was used to get the dynamics of the mean canopy heights within the EC systems target area. For the NEON stations, biomass estimates were derived for each year at each site using the neonPlants R package (https://github.com/NEONScience/neonPlants). Separate algorithms were used to get herbaceous and woody biomass from their respective data products on the NEON data portal, Herbaceous clip harvest (DP1.10023.001; NEON (49) (2025)) and Vegetation structure (DP1.10098.001; NEON (50) (2025)). The canopy height data were extracted from NEON Airborne Observation Platform (AOP) Ecosystem structure (DP3.30015.001; NEON (48) (2025)) data product.

Half‐hourly continuous data, both meteorological and EC data, were collected from the networks' data portal (https://data.icos‐cp.eu/portal/ and https://data.neonscience.org for ICOS and NEON, respectively). The main meteorological variables used in the analysis were the air temperature (TA, °C) and relative humidity (RH, %), incoming and outgoing shortwave and longwave radiations (SW_IN, SW_OUT, LW_IN, and LW_OUT, respectively, W m^−2^) including surface albedo (A, %) and net radiation (NETRAD, W m^−2^), soil heat flux (G, W m^−2^) and soil heat storage flux (S_G_, W m^−2^). As for the ICOS data, each meteorological variable comes with a set of metadata including sensors' models and position, which have been used in the radiation corrections.

The half‐hourly EC datasets were collected from the L2 ICOS and NEON data products, elaborated by means of respective network processing pipelines (ICOS: Sabbatini et al. 2018; Vitale et al. 2020; Metzger et al. 2022). Although they differ in some basic processing steps—the most notable differences are the spectral corrections and quality filtering—these pipelines share the same post‐processing stage resulting in friction velocity (u*) filtered, gapfilled, and partitioned fluxes (the ONEFlux pipeline, Pastorello et al. 2020, more details are given in the Supporting Information).

EC half‐hourly data used in the present study included sensible and latent heat fluxes (H and LE respectively, W m^−2^); heat and evapotranspiration storage fluxes derived from air temperature and humidity profile measurements when available (S_H_ and S_LE_ respectively, W m^−2^) and by the single point approximation (S_H_1P_ and S_LE_1P_, respectively, W m^−2^) otherwise; gross primary productivity (GPP, μmol CO_2_ m^−2^ s^−1^) as derived from daytime partitioning of net ecosystem exchange (Lasslop et al. 2009).

Gaps in flux data, mainly coming from quality evaluation in the raw data processing and low turbulence filtering, were filled using the marginal distribution sampling (MDS) method as described in Reichstein et al. (2005). Gap‐filled data at 30 min resolution were used for aggregations up to annual scales. The EBC analysis was performed over both 30 min data (with and without gaps) and longer period aggregation data.

For the ICOS stations, the footprint of the EC systems was estimated based on the Flux Footprint Prediction (FFP) model by Kljun et al. (2015). Flux footprints at 30 min resolution were estimated for the whole data span according to the availability of required model input data and used to estimate the ratio of contribution of the footprint area to the target area. In addition, footprint climatologies (aggregation of footprints over longer time steps) were derived for the year 2024 and used in the radiation correction analysis.

In addition to the L2 data, the 2024 L1 data were also used for the ICOS stations. This dataset shares the same processing parameters and QC rules used in the L2 data, yet differs in the application of the type of spectral correction for the high frequency losses. In the L2 data the “in situ” method by Fratini et al. (2012) is applied to assess the specific system attenuation of energy spectra. This approach utilizes all the available high‐quality spectra and co‐spectra, and accounts for, as well as compensates for, the effects of RH on LE fluxes. In contrast, for the L1 data the “analytical” method is used (Moncrieff et al. 1997), which models the major sources of flux attenuation through a mathematical formulation, independent of the actual RH values.

### 2.2 Energy Balance Terms Calculations

According to the principle of energy conservation and assuming no advective and lateral fluxes, the surface energy balance at an EC station requires that the sum of sensible (H) and latent (LE) heat fluxes measured at height *z*_m_, referred to as turbulent energy (TE), equals the available energy (AE) at the surface.

The available energy is defined as the net radiation, corrected for changes in energy storage within the ground, in the air column beneath *z*_m_, and in the aboveground biomass. This must hold for each flux averaging interval, the standard 30 min period commonly employed in EC measurements included.

#### 2.2.1 Available Energy

The theoretical available energy (AE, W m^−2^) at the surface, assuming no lateral flow of energy not captured by the EC measurements, can be expressed as follows:

(1) $$\mathrm{AE}=\text{NETRAD}-G-S_{G}-S_{H}-S_{\mathrm{LE}}-S_{\mathrm{pho}}-S_{\mathrm{bio}}$$

where NETRAD is the net radiation absorbed by the surface, G is the measured heat flux into soil, S_G_ is the storage heat flux in the soil layer (between the soil surface and the G measurement point), S_H_ and S_LE_ are the changes in heat storage in the air column below the EC system associated with air temperature and water vapor respectively, S_pho_ is the biochemical energy absorbed during photosynthesis, and S_bio_ is the heat storage change in the biomass. All the energy fluxes by convention have a negative sign when entering in the ecosystem virtual box (volume between the EC system and the soil surface) and positive when exiting, either toward the atmosphere or in the soil. NETRAD was computed at each station using the measured incoming and outgoing short‐ and long‐wave radiation as NETRAD = SW_IN + LW_IN − SW_OUT − LW_OUT. G was measured using self‐calibrating soil heat flux plates (installed at 0.05 m depth), and corrected using calorimetric estimates of the heat storage in the soil layer above the plates (S_G_) derived from soil temperature and moisture measurements as:

(2) $$S_{G}=\sum_{i=1}^{i=n}\left(c_{s,i}\frac{{\mathrm{\Delta T}}_{s,i}}{\mathrm{\Delta t}}\Delta z_{i}\right)$$

where *c*_*s,i*_ (J °C^−1^ m^−3^) is the soil volumetric heat capacity calculated as a function of soil moisture at the layer *i* (see the Supporting Information for details), *ΔT*_*S,i*_ (°C) is the temporal temperature change (*T*_*S,t+Δt*_ *− T*_*S,t*_) measured at layer *i*, *Δz*_*i*_ (m) is the thickness of layer *i*, *Δt* is the averaging interval (1800 s), and the summation is performed over all layers between the soil surface and the depth at which G is measured. G and S_G_ values represent the spatial average of all available soil heat flux measurements at each station, with 3 to 5 measurement points for ICOS stations and 5 measurement points for NEON stations.

S_H_ and S_LE_ fluxes (W m^−2^) were estimated from temperature and moisture profile measurement along the air column respectively by (Haverd et al. 2007; Leuning et al. 2012):

(3) $$S_{H}=\rho_{a}c_{p}\sum_{i=1}^{n}\left(\frac{\Delta {\mathrm{TA}}_{i}}{\mathrm{\Delta t}}\Delta z_{i}\right)$$

(4) $$S_{\mathrm{LE}}={⍴}_{d}\;\lambda \;M_{w}\sum_{i=1}^{n}\left(\frac{\Delta X_{w,i}}{\mathrm{\Delta t}}\Delta z_{i}\right)$$

where in (3) *ρ*_*a*_ is the air density (kg m^−3^), *c*_*p*_ is the specific heat of air at constant pressure (J kg^−1^ K^−1^), *TA*_*i*_ is the air temperature (K), *Δz* is the profile layers depth (m), *i* is the index of the respective layer, in (4) *ρ*_*d*_ is the dry air molar density (mol m^−3^), 𝜆 is the latent heat of vaporization for water (J kg^−1^), *M*_*w*_ is the molecular mass of water (kg mol^−1^) and *X*_*w*_ is the mole fraction of water vapor relative to dry air (mol mol^−1^). For stations without profile measurements (ICOS stations with EC measurement height < 4 m), the two terms were approximated using the one‐point approach, based on the temperature and moisture measurements at the EC system height and assuming no vertical gradient below. The one‐point estimations were performed also for the stations with profile measurements and both estimates were used and compared in the EBC analysis.

The energy used by the plants' biological activity was estimated by the following equation (Moderow et al. 2009; McGloin et al. 2018).

(5) $$S_{\mathrm{pho}}=-\mu \mathrm{GPP}$$

where *μ* is the specific biochemical energy for conversion due to photosynthesis (10.88 × 10^6^ J kg CO_2_^−1^) and GPP was converted from μmol CO_2_ m^−2^ s^−1^ to kg CO_2_ m^−2^ s^−1^ using the molar mass of CO_2_ (0.044 kg CO_2_ mol^−1^).

Heat storage change in the above ground biomass was calculated as (Moderow et al. 2009; McGloin et al. 2018):

(6) $$S_{\mathrm{bio}}=m_{v}c_{v}\frac{\underline{{\mathrm{\Delta T}}_{v}}}{\mathrm{\Delta t}},$$

where *m*_*v*_ is the wet mass of wood and leaves (kg m^−2^), *c*_*v*_ is the specific heat capacity of biomass components (2958 J K^−1^ kg^−1^), $\underline{{\mathrm{\Delta T}}_{v}}$ is the change in biomass temperature (K) here approximated by the layer‐weighted average air temperatures calculated from temperature profile measurements. To get *m*_*v*_ from inventory data, we adopted an average dry‐to‐fresh weight conversion factor of 2 (i.e., dry biomass ~40%–60% of fresh biomass), consistent with typical values reported in destructive sampling studies (e.g., Puc‐Kauil et al. 2020; Verwijst and Telenius 1999).

#### 2.2.2 Net Radiation Corrections

In addition to considering the NETRAD as derived from measured components, a series of corrections were applied to the incoming and outgoing radiation terms, and their impact on the EBC was analyzed. The errors considered were those deriving from: (1) incoming radiation measurement over sloping terrain, (2) possible shadow‐casting below the measurement point by the surrounding topography, (3) outgoing radiation disturbance caused by the inclusion of the hosting structure (e.g., tower, trellis) in the field‐of‐view (FoV) of the radiometer, (4) possible mismatch between radiative sources within the radiometer FoV and the turbulent fluxes footprint. Although computations were performed for all stations with available data, results are only reported for stations where the single correction is significant. In particular, the slope effect (1) is reported only for non‐flat terrains (landscape‐scale slope ≥ 2° ≈3.5%), the FoV mismatch (4) is only reported for stations where the overlap between the EC flux footprint and the FoV of the radiometer is poor, that is, when the overlapping area represents less than 60% of the larger of the two areas. The first potential error source was evaluated for both ICOS and NEON stations, whereas assessment of the remaining sources was restricted to ICOS stations due to data availability constraints. Specifically, comprehensive tower setup and dimensioning data were not uniformly available across NEON sites at the time of this analysis. Future incorporation of expanded NEON ancillary data streams would enable cross‐network comparison of all error sources.

##### 2.2.2.1 Sloping Terrain

Radiation sensors are usually mounted horizontally, but the actual incoming radiation reaching the surface depends on the slope and aspect of the surface. For example, a south facing sloping surface in the northern hemi‐sphere will absorb more energy than measured by a horizontally mounted instrument (Olmo et al. 1999; Leuning et al. 2012), as setup in the ICOS and NEON stations (Papale et al. 2023). Incoming short‐wave radiation measurements were corrected for this issue following the method proposed by Olmo et al. (1999) and applied in, for example, Serrano‐Ortiz et al. (2015) and McGloin et al. (2018). The method requires calculating, for each averaging interval, the solar zenith angle (*θ*_*s*_), the angle between the normal to the inclined surface and the direction of the sun (𝛹), and the ratio of measured radiation to extraterrestrial horizontal irradiance (clearness index, *k*_*t*_). The incoming solar radiation for an inclined surface (SW_IN(𝜓)) can be estimated by.

(7) $$\mathrm{SW}\_\text{IN}\left(\psi \right)=\mathrm{SW}\_\text{IN}\;\exp\left[-k_{t}\left(\psi^{2}-\Theta_{s}^{2}\right)\right]\left[1-\mathrm{ALB}\;\sin^{2}\left(\psi /2\right)\right]$$

where ALB is the surface albedo estimated as the ratio of SW_OUT and SW_IN measurements. The measured SW_IN values were then replaced by the SW_IN(𝜓) values in the net radiation formula to get the slope‐corrected net radiation (NETRAD_slope_). SW_OUT, LW_IN and LW_OUT were not corrected as they do not differ significantly whether measured by horizontal or slope‐parallel radiometers (Holst et al. 2005; McGloin et al. 2018).

##### 2.2.2.2 Topographic Shadowing

Accounting for topographic effects on spatial radiation patterns has been widely studied in ecological and remote sensing modelling (Dozier 2022; Druel et al. 2025), in weather forecasting (Ruiz‐Arias et al. 2011), and in the EC context (Hammerle et al. 2006; Hrach et al. 2021; Wohlfahrt et al. 2016). Especially in mountain areas, horizon shadowing from surrounding topography may occur when a surface lies below the local horizon, causing direct‐beam surface irradiance to be blocked by nearby peaks while the radiometer could remain in full exposure to light. To characterize the topography, the Copernicus Digital Surface Model (DSM) GLO‐30 (ESA 2024) at 30 m resolution was retrieved in 20 km x 20 km frames around each EC tower using a custom algorithm developed on GEE. Topographic horizon shadowing was accounted for by separating the direct and diffuse components of incoming shortwave radiation (SW_IN and SW_DIF respectively). For each 30 min time step, the solar elevation (90° − zenith angle) was compared with the horizon elevation at the corresponding azimuth derived from the DSM. When the solar elevation was lower than the local horizon, direct‐beam shortwave radiation was assumed to be fully obstructed by surrounding topography, and incoming shortwave radiation (SW_shadow_) was set equal to the diffuse component only. At sites where SW_DIF was not available, it was estimated using an empirical parameterization based on the clearness index and solar elevation angle (Reindl et al. 1990). Diffuse fraction was only estimated for time steps with positive extraterrestrial radiation and SW_IN exceeding 5 W m^−2^, while values during nighttime conditions were excluded. The reflected shortwave component was neglected as assumed to be irrelevant. Uncertainty associated with the estimation of the diffuse fraction is expected to be large, especially under cloudy conditions, but it was considered preferable to neglecting diffuse radiation entirely. SW_IN was replaced by SW_IN_shadow_ in the NETRAD formulation and recalculated accordingly (NETRAD_shadow_).

##### 2.2.2.3 Mounting Tower Interference

Measurement of SW_OUT and LW_OUT may be biased by the inclusion of the mounting tower in the FoV of the downward‐looking radiometers both because the tower obstructs part of the FoV, and because the reflectance (albedo, ALB) and emissivity (ε) of the metallic structure impact the measured short‐wave and long‐wave canopy reflected/emitted radiations, respectively (Kidston et al. 2010). This error was addressed for each ICOS station, accounting for specific instrumentation setup and tower geometry. To this aim, it has accounted for the radiometer FoV (considering a total angle of 170° for pyranometers and 150° for pyrgeometers, for all the stations), its mounting height above the surface, the horizontal distance from the tower, and the tower cross‐sectional dimensions. The vegetation canopy height was subtracted from the mounting height to determine the effective vertical extent of the tower intercepted by the FoV. The tower was modeled as a vertical rectangle, with a width equal to the tower's cross‐section and a height corresponding to the visible vertical profile within the FoV. To account for the void spaces in the lattice tower structures, the physical cross‐section width was arbitrarily reduced by 40%. The corresponding solid angle subtended by the tower was computed using an exact geometric formulation based on spherical projection. The total radiometer FoV solid angle was also calculated as a reference. The disturbance fraction (*δ*_*frac*_) was then derived as the ratio between the solid angle of the tower and the total FoV solid angle. Following Kidston et al. (2010), the variables *ALB* and *ε* sensed within the FoV of the downward facing pyranometer and pyrgeometer were defined as

(8) $${\mathrm{ALB}}_{\mathrm{FoV}}=\left(1-\delta_{\text{frac}}\right){\mathrm{ALB}}_{g}+\delta_{\text{frac}}{\mathrm{ALB}}_{t}$$

(9) $$\varepsilon_{\mathrm{FoV}}=\left(1-\delta_{\text{frac}}\right)\varepsilon_{g}+\delta_{\text{frac}}\varepsilon_{t}$$

where the subscripts *g* and *t* refer to the ground and tower, respectively. For ALB_*t*_, a value of 0.2 was used for common ferrous structures, given a range of 0.08–0.25 for bare steel, oxidized metal, iron, unpainted steel (Morini et al. 2017; PVPMC 2025). Equation (8) was then rearranged to get ALB_*g*_, and the ratio between ALB_FoV_ and ALB_*g*_ was used as a correction factor for SW_OUT. It was assumed that (a) SW_OUT is proportional to the measured albedo (as SW_OUT = ALB × SW_IN) and that (b) the irradiance illuminating the portion of the tower seen by the sensor is equal to that on the whole scene.

To correct LW_OUT it was applied the same strategy as for SW_OUT. The thermal emissivity of the towers were set to *ε*_*t*_ = 0.3, considering average values for aluminum and iron (Fluke 2025; Kidston et al. 2010). The emissivity within the FoV was estimated by (Faysash and Smith 1999; Jin and Liang 2006)

(10) $$\varepsilon_{\mathrm{FoV}}=\frac{\mathrm{LW}\_\text{OUT}-\tau \mathrm{LW}\_\text{IN}-{\mathrm{LW}}_{\mathrm{ATM}}}{\tau \left(\sigma T_{\text{surf}}^{4}-\mathrm{LW}\_\text{OUT}\right)}$$

in which *τ* is the atmospheric transmittance, here considered to equal 1 as for the case of a dry atmosphere, *σ* is the Stefan–Boltzmann constant (5.670374419 × 10^−8^ W m^−2^ K^−4^) and *T*_surf_ is the surface temperature (*K*). The atmospheric longwave radiative emission between the sensor and the surface (LW_ATM_) was assumed to be negligible and the term set to 0. In absence of direct measurements of *T*_surf_, air temperature (TA) is often used as a proxy. However, *T*_surf_ and TA are known to differ due to thermal inertia and surface energy balance processes, with *T*_surf_ generally exceeding TA during daytime and responding differently at night (e.g., Gallo et al. 2011). Based on observed typical daytime and nighttime *T*_surf_ − TA differences, we approximated *T*_surf_ ≈ TA + *ΔT*, with *ΔT* set to +4 *K* in daytime and +2 *K* at night, to partially correct this bias. Under similar assumptions as for SW_OUT, from Equation (9) *ε*_*g*_ was derived and the ratio between *ε*_FoV_ and *ε*_*g*_ was used as a correction factor for LW_OUT.

The measured SW_OUT and LW_OUT values were then replaced by the corrected respective values in the net radiation formula to get the tower‐interference corrected net radiation (NETRAD_tower_).

##### 2.2.2.4 Radiometer and EC Field‐Of‐View Consistency

The AE of different land covers is predominantly influenced by their specific albedo and surface temperature. This means that heterogeneous surfaces, composed of a mixture of different cover types, can absorb and emit energy in significantly different ways (Schmid 1997). The surface projections of the FoV of the downward‐looking radiometers and the “FoV” of the EC system, that is, its footprint, rarely match, often sensing different patches of land use. The former is dependent on the optical characteristic of the sensor and its mounting height and remains constant over time, the latter depends on atmospheric and turbulence conditions, surface roughness, measurement height, and varies over time. Direct comparisons between AE and the TE, particularly when the surface spatial variability is large, can thus either worsen the energy balance closure or falsely enhance it (Mauder et al. 2024; Ramtvedt and Pirk 2022; Sánchez et al. 2010). Net radiation measurements should be adjusted for such a mismatch to improve the consistency with concurrent TE exchange at the surface. To this purpose, a spatial analysis was done integrating remote sensing data with the spatial features of the EC footprint and radiometer FoV, in order to apply a correction coefficient to measured SW_OUT (300–3000 nm) and LW_OUT (3000–30,000 nm). Sentinel‐2 and Landsat 9 data were used to correct SW_OUT and LW_OUT, respectively. For each ICOS station in 2024, the EC footprint climatology was calculated using FFP (Kljun et al. 2015). Each footprint was georeferenced and rasterized using a custom function (ICOS ETC, FFP‐utils, https://github.com/icos‐etc/FFP‐utils), clipped to the 70% cumulative contribution bounds (EC_FoV_), and re‐scaled such that the total sum of actual contributions equals 1. Then, the projected circular area of the radiometers FoV was calculated based on the measurement height above the canopy top and FoV total angle (rad_FoV_), and georeferenced as for the concurrent footprint data. Sentinel‐2 level 2A and Landsat‐9 L2 images for 2024 were collected using GEE. Each image was filtered for cloud coverage (< 50%) and masked for clouds and shadows. To estimate the surface reflectance (SR) in the SW range, Sentinel images were aggregated at pixel level by calculating an average between the visible bands (2–4: 490–665 nm) and near‐infrared bands (5–8, 11 and 12: 705–842 nm, 1610 nm and 2190 nm). For the LW spectral domain, Landsat‐9 Land Surface Temperature (LST, derived from the band 10, 10,800 nm) at 30 m resolution was used. Finally, images were temporally aggregated to a 30 days interval using a pixel‐oriented median.

Temporal‐aggregated SR and LST images were then clipped at the site level for both EC_FoV_ and rad_FoV_ boundaries (SR_EC_ and SR_rad_, LST_EC_ and LST_rad_). Time steps where valid pixel medians were less than 50% within each area of interest were discarded. Pixel values were integrated into EC_FoV_ and rad_FoV_ weighting for the respective pixel contribution, that is, the footprint values (previously resampled to spatially match SR_EC_ and LST_EC_) for EC_FoV_, constant weights in $\Sigma_{i=1}^{n}$ *w*_*i*_ = 1 for rad_FoV_. Lastly, the ratios used to correct SW_OUT and LW_OUT were obtained respectively by

(11) $$\delta_{\mathrm{FoV}|\mathrm{SW}}=\frac{{\mathrm{SR}}_{\mathrm{rad}}}{{\mathrm{SR}}_{\mathrm{EC}}}$$

(12) $$\delta_{\mathrm{FoV}|\mathrm{LW}}=\frac{{\mathrm{LST}}_{\mathrm{rad}}}{{\mathrm{LST}}_{\mathrm{EC}}}$$

As for the previous correction, it was assumed linearity both between SW_OUT and the SR in the visible bands, and between LW_OUT and the LST in the thermal near infra‐red bands, and that their ratios remain invariant at monthly scale which, although follows the seasonality, is a simplification adopted for this study.

After having adjusted the SW_OUT and LW_OUT measurements using the respective SR ratios as $\mathrm{SW}\_{\text{OUT}}_{\mathrm{FoV}}=\mathrm{SW}\_\text{OUT}\;\delta_{\mathrm{FoV}|\mathrm{SW}}$ and $\mathrm{LW}\_{\text{OUT}}_{\mathrm{FoV}}=\mathrm{SW}\_\text{OUT}\;\delta_{\mathrm{FoV}|\mathrm{LW}}$, the corrected values were used in the net radiation formula instead of the measured ones to get the radiometer‐EC FoV consistency corrected net radiation (NETRAD_FoV_).

#### 2.2.3 Turbulent Energy

H and LE were processed using three versions with progressively increasing quality control (QC) stringency:

1. Unfiltered fluxes (*uncorrected*): only physically aberrant data points falling outside plausible flux ranges were removed, with no additional quality screening applied;
2. Fully quality‐filtered fluxes (*fullQC*): data were filtered according to the network‐specific optimal quality assurance protocols. For ICOS, we applied the method described in Vitale et al. (2020), which employs a suite of statistical tests to detect systematic errors and outliers, removing observations with severe quality issues (flag = 2). For NEON, quality flags were assigned when either (i) more than 10% of high‐frequency measurements failed multiple statistical tests (e.g., range checks, step tests, persistence tests, spike detection, sensor diagnostic), or (ii) more than 20% of data were missing within any 30‐min averaging period. Turbulent flux quality flags inherit the quality assessment from their constituent variables (e.g., vertical wind velocity and H_2_O for LE) and are further combined with flags from stationarity and integral turbulence characteristic tests (Foken and Wichura 1996) to generate the final filtering decision (Metzger et al. 2022). Additionally, NEON scientists manually review data prior to each year's release to apply science review flags that catch anomalous data not captured by autonomous data flagging (e.g., issues with gas validation system or leaks in the gas system).
3. Low‐turbulence filtering (u*_filter): starting from the *fullQC* version, periods with potentially non‐negligible advective fluxes arising from insufficient turbulent mixing were removed based on site‐specific friction velocity (u*) thresholds derived following Papale et al. (2006).

##### 2.2.3.1 ICOS‐Specific Analysis

Beyond the impact of H and LE quality filtering on EBC, we also compared high‐frequency spectral correction methods. Specifically, we evaluated potential underestimation of LE fluxes under medium‐to‐high relative humidity (RH) conditions when using the analytical correction method (ICOS Level 1 data) versus the RH‐dependent empirical approach implemented in the ICOS Level 2 processing workflow.

Additional quality filters were applied only to ICOS datasets due to temporary unavailability of required data in NEON archives:

- Turbulence‐related filtering (*SSITC*): data were filtered using the combined flagging policy of Foken et al. (2004), which integrates results from steady‐state and integral turbulence characteristic tests, rejecting measurements with low quality scores (flag = 2 following Foken and Wichura 1996).
- Flux footprint representativeness (*FFPrep*): half‐hourly observations were excluded when the flux footprint contribution from the target ecosystem area fell below 70%. This filter yielded negligible marginal improvements in EBC relative to u* filtering and was therefore excluded from further analysis. This outcome was anticipated, as both filters target similar conditions (inadequate turbulence), and ICOS station processing already excludes wind sectors characterized by off‐target land cover types.

Results obtained with *SSITC* and *FFPrep* filtering are not reported here because their impact was only minor.

### 2.3 Energy Balance Closure Quantification

The EBC was originally evaluated using three methods. First, an ordinary least squares (OLS) regression was performed between the TE (H + LE) and the AE, assuming no random errors in the independent variables (AE). However, this assumption ignores measurement errors in the independent variables, leading to a downward bias in the slope estimate (Wilson et al. 2002). To address this issue, the reduced major axis (RMA) regression was employed. Unlike OLS, RMA accounts for errors in both variables, making it more appropriate when both axes contain measurement uncertainties (McArdle 1988; Warton et al. 2006) as is common in energy balance studies. This potentially reduces biases and provides a more accurate estimate of the relationship between TE and AE. However, although some difference in the EBC magnitude among OLS and RMA regression results emerged, EBC patterns remained similar and results are reported here for RMA only (comparisons among the two methods in the Supporting Information, Figure S3).

Then, in order to evaluate the EBC when fluxes are integrated over longer periods (daily to annual scales) the energy balance bulk ratio (EBR) was used as a third method. The EBR is defined as the ratio of the sum of TE to the sum of AE over a defined continuous measurement period such as daily or monthly (Ramtvedt and Pirk 2022; Wilson et al. 2002), providing an integrated assessment of the energy closure. Only for this evaluation with fluxes integrated over longer periods, the sums of TE and AE were obtained from gap‐filled data.

To evaluate the marginal contribution of each AE component and TE quality filtering to the energy balance closure, regressions and ratios were calculated for each TE ~ AE pairing, considering all possible combinations (Table 2).

**TABLE 2:** Available energy (AE) and turbulent energy (TE) formulations used for energy balance closure analysis.

Available energy		Turbulent energy	
Code	Meaning	Code	Meaning
AE1	NETRAD	TE_uncorrected	Unfiltered energy fluxes
AE2	NETRAD−G	TE_fullQC	Fully quality‐filtered fluxes
AE3	NETRAD−G–SG	TE_u*_filter	Fluxes filtered for low‐turbulence conditions
AE4	NETRAD−G−SG−SH−SLE		
AE5	NETRAD−G−SG−SH−SLE−Spho		
AE6	NETRAD−G−SG−SH−SLE−Spho−Sbio		

*Note:* AE formulations (AE1–AE6) represent progressive inclusion of additional energy components; TE formulations reflect different quality filtering levels. G is the soil heat flux, S_G_ is the soil storage heat flux, S_H_ and S_LE_ are the changes in heat storage in air, S_pho_ is the biochemical energy absorbed during photosynthesis, and S_bio_ is the heat storage change in the above ground biomass.

We note that the simpler AE component (AE1) was based solely on measured net radiation. It was not intended to represent the conventional definition of available energy, but rather served as a conceptual baseline to quantify how progressively including additional energy components (e.g., ground heat flux and storage terms) affects energy balance closure estimates. Although topographic and instrumental corrections were computed and evaluated in a dedicated sensitivity analysis, they were not applied in the primary cross‐site assessment. This choice was motivated by two considerations. First, avoiding site‐specific corrections allowed us to isolate the generalized effect of energy accounting methodologies from local terrain and instrumental artifacts, thereby enabling a more robust inter‐site comparison. Second, applying corrections nonuniformly across sites would have introduced systematic bias, as not all corrections were applicable or feasible at all sites due to either missing ancillary data or lack of correction necessity; comparing sites with and without corrections would have confounded the analysis with unequal sample sizes and heterogeneous data treatment. An additional consideration is that sublimation was not differentiated from evaporation in our latent heat flux calculations. Conservative error estimation yielded < 1 Wm^−2^, indicating that this simplification does not affect energy balance closure analysis.

## 3 Results

### 3.1 General EBC Closure Improvement

**FIGURE 1:**
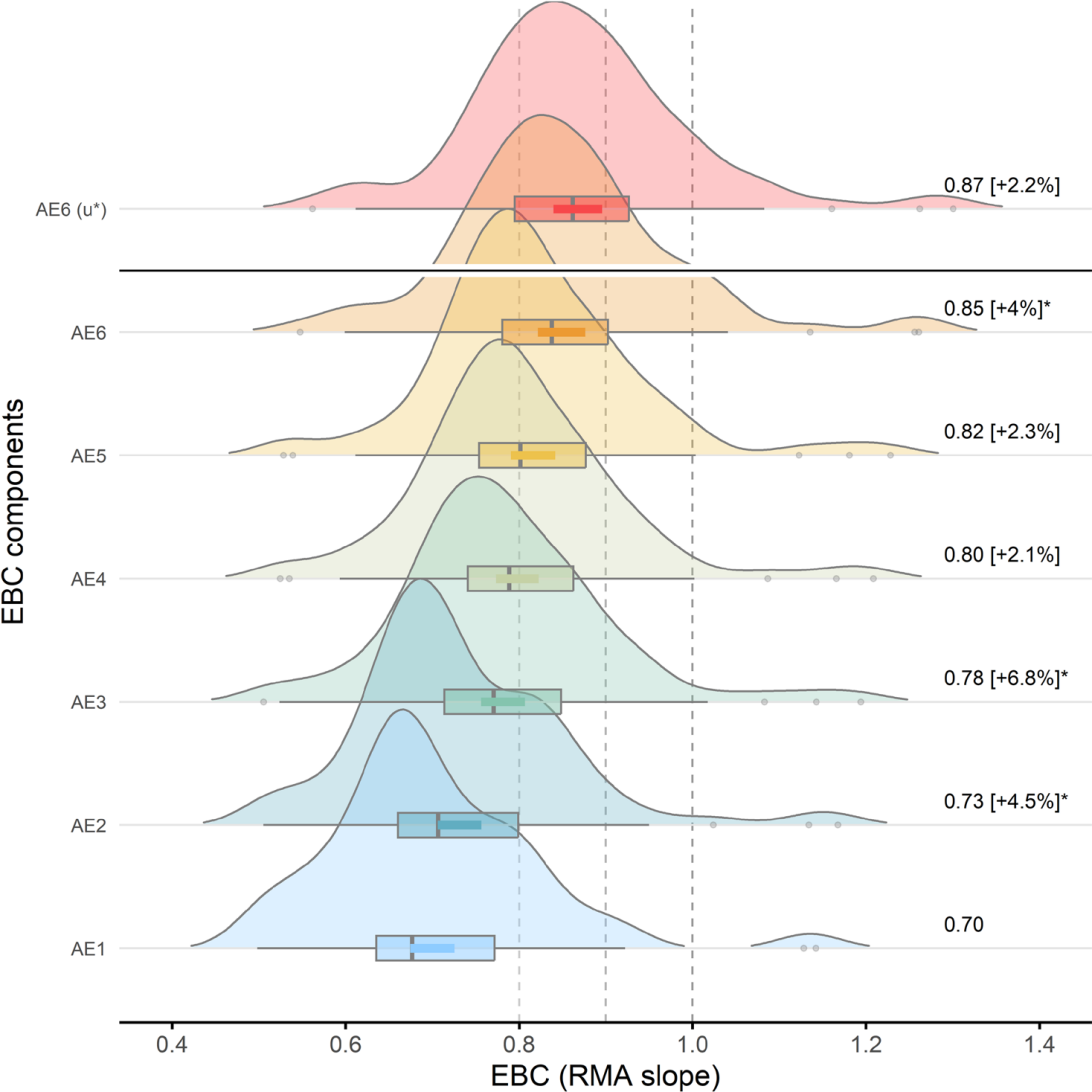
Step improvement of the Energy Balance Closure (EBC), all stations considered (*n* = 84, 38 ICOS and 46 NEON), reported as statistics of the RMA slopes between measured turbulent energy (TE) and available energy (AE) at 30 min time‐scale. AE1‐AE6: Progressively complete AE formulations: AE1 = NETRAD; AE2 = NETRAD − G; AE3 = NETRAD − G − S_G_; AE4 = NETRAD − G − S_G_ − S_H_ − S_LE_; AE5 = AE4 − S_pho_; AE6 = AE5 − S_bio_, regressed against the top‐quality filtering TE version (TE_fullQC_).The topmost distribution (AE6(u*)) is referred to the most accurate condition in this study in which TEs, in addition to being QC filtered with the top‐quality methods, are filtered for low friction velocity conditions (TE_u*_filter ~ AE6). EBC statistics are reported as density distributions, boxplots, and 95% CI (thick colored lines) of RMA slopes. Numbers on the right are mean EBC values and incremental improvements, with respect to the previous mean. Asterisks highlight significant differences at progressive steps (Wilcoxon post hoc pairwise test, *ɑ* = 0.05). Vertical dashed lines are for reference, ideal at 1.

Looking at all the stations analyzed as a whole, the energy balance closure improved systematically, at 30 min resolution, as progressively more complete definitions of available energy (AE) were considered (Figure 1). Using only the measured net radiation (AE1) yielded the lowest closures, averaging 0.70 ± 0.01 (median slope = 0.68, 95% CI: 0.67–0.73). Subtracting soil heat flux (G, AE2) increased the closure by +4.5%, with an average value of 0.73 ± 0.01 (0.71, 0.71–0.76). Further inclusion of soil heat storage (S_G_, AE3) produced the largest single improvement (+6.8%, 0.78 ± 0.01, 0.77, 0.76–0.81), highlighting the importance of below‐ground heat storage and its measurement. Including air‐column heat storage terms (S_H_ and S_LE_, AE4) resulted in a smaller +2.1% increment (0.80 ± 0.01, 0.79, 0.77–0.82), similar to the +2.2% increase obtained by including photosynthetic energy consumption (S_pho_, AE4), which further improved closure to 0.82 ± 0.01 (median = 0.80, 0.79–0.84). Inclusion of biomass heat storage (S_bio_, AE6) increased the regression slopes further, yielding an additional improvement of up to +3.9% and raising the mean closure to 0.85 ± 0.01 (median = 0.83, 95% CI: 0.82–0.88). This result indicates that biomass heat storage, although often neglected, significantly contributes to the surface energy balance.

The highest average closure, 0.87 ± 0.01 (median = 0.86, 95% CI: 0.84–0.90) corresponding to an efficiency increment of +2.2%, was achieved when applying u* filtering to the TE compound in combination with the most complete AE definition. This result is consistent with the established effect of turbulence filtering in reducing low‐mixing biases. Overall differences between groups were statistically significant (Kruskal–Wallis rank sum test, *α* = 0.05). In general, accounting for all the major AE components and properly filtering TE under low‐turbulence conditions improved half‐hourly energy balance closure by 16% on average (interquartile range (IQR) 11%–20%, *n* = 79), with maximum improvements approaching 40%. Although closure did not consistently reach unity, the progressive convergence of regression slopes toward the 1:1 line indicates that a substantial fraction of the EBC gap arises from neglected energy storage terms rather than measurement error alone.

### 3.2 EBC Across Ecosystem Types

**FIGURE 2:**
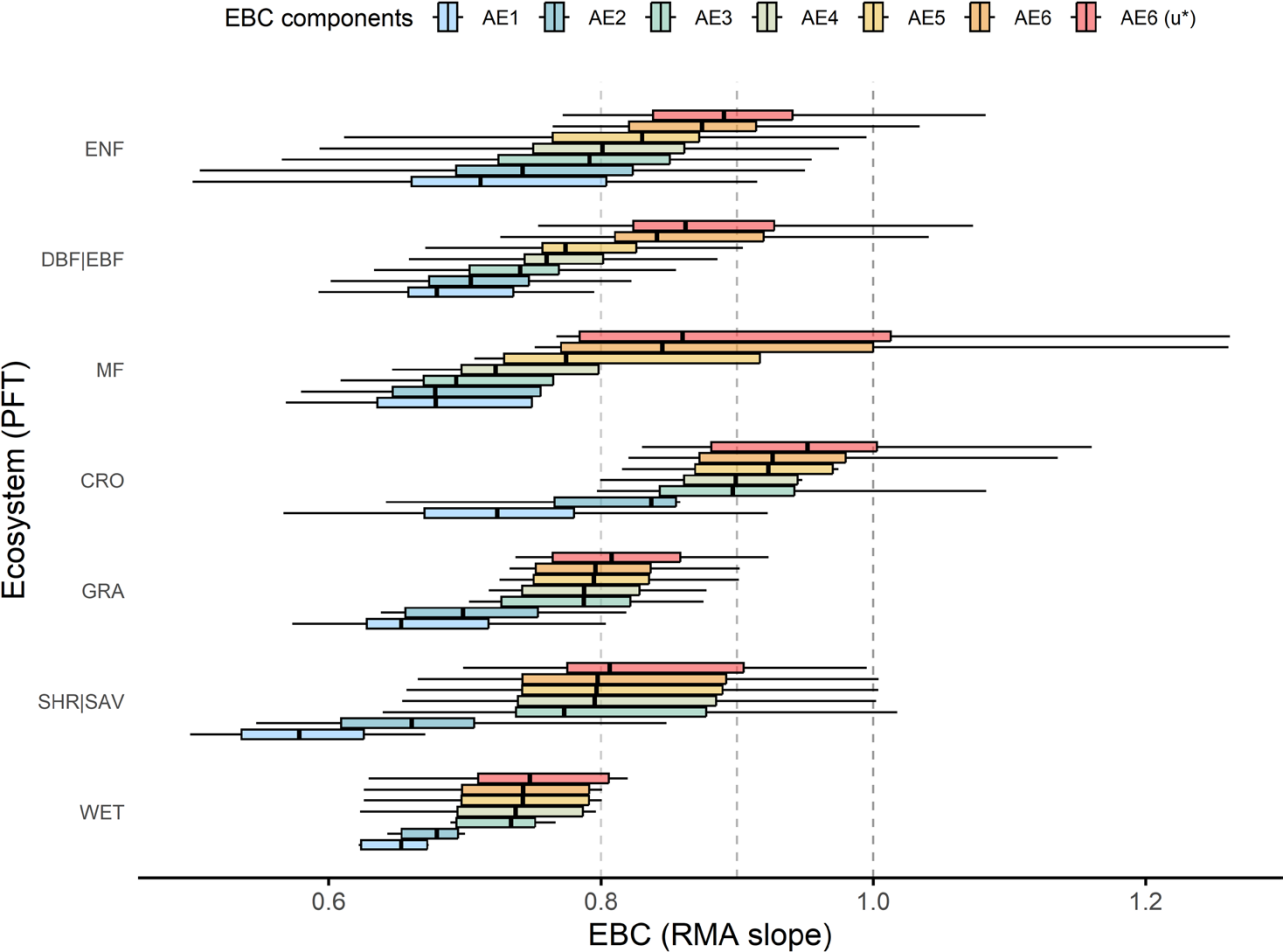
Boxplot representation of the step improvement of the Energy Balance Closure (EBC), all stations considered, by ecosystem type (PFT = plant functional type), reported as statistics of the RMA slopes between TE and AE for each PFT. The TE ~ AE pairings are the same as in Figure 1: Full_QC TE regressed against AE1 = NETRAD; AE2 = AE1 − G; AE3 = AE2 − S_G_; AE4 = AE3 − S_H_ − S_LE_; AE5 = AE4 − S_pho_; AE6 = AE5 − S_bio_. AE6(u*) is AE6 regressed against u*‐filtered TE. PFT sample sizes: Wetlands (WET, *n* = 7), savannas/shrublands (SHR|SAV, *n* = 7: 5 OSH, 1 CSH, 1 SAV), grasslands (GRA, *n* = 14), croplands (CRO, *n* = 6), mixed forests (MF, *n* = 5), evergreen needleleaf forests (ENF, *n* = 27), deciduous/evergreen broadleaf forests (DBF|EBF, *n* = 18: 16 DBF, 2 EBF). Vertical dashed lines are for reference, ideal at 1.

The EBC analysis across all sites revealed distinct variability patterns both among and within plant functional types (PFT) (Figure 2). Two broad ecosystem groups can be distinguished: short‐canopy and tall‐canopy ecosystems. Focusing on the optimal TE ~ AE pairing (AE6(u*), reddish box plots in Figures 1 and 2), within the short canopy group, natural and semi‐natural ecosystems, including wetlands (WET, *n* = 7), savannas/shrublands (SHR|SAV, *n* = 7: 5 OSH, 1 CSH, 1 SAV), and grasslands (GRA, *n* = 14) exhibited relatively lower energy balance closures around 80%. Mean EBCs ranged from 0.77 ± 0.04 in wetlands (median = 0.75, 95% CI: 0.69–0.85) to 0.82 ± 0.02 in grasslands (median = 0.81, 95% CI: 0.79–0.85). In contrast, croplands (CRO, *n* = 6) showed the highest energy budget closure, with a mean EBC of 0.96 ± 0.05 (median = 0.95, 95% CI: 0.87–1.06).

Tall‐canopy ecosystems, including evergreen needleleaf forests (ENF, *n* = 27), deciduous/evergreen broadleaf forests (DBF|EBF, *n* = 18: 16 DBF, 2 EBF), and mixed forests (MF, *n* = 5), exhibited generally better closure performances, with EBC values around 90%. Mean regression slopes ranged from 0.89 ± 0.20 (median = 0.86, 95% CI: 0.84–0.93) in broadleaf forests to 0.94 ± 0.11 (median = 0.86, 95% CI: 0.71–1.16) in mixed forests. Evergreen needleleaf forests, although similar to broadleaf forests in mean and variability, exhibited a higher median of 0.89. This pattern aligns with prior studies linking closure efficiencies to structural and functional ecosystem characteristics, although some PFT‐specific values differ from previous reports (Stoy et al. 2013; Mauder et al. 2024).

When considering all EBC formulations together, the PFT groups differ significantly from each other (Kruskal–Wallis test, *p* < 0.01), with varying degrees of significance in pairwise comparisons (Wilcoxon rank sum test, *α* = 0.05; Supporting Information, Table S4). Croplands clearly differentiate from all other ecosystem types. However, pairwise comparisons between PFTs for individual EBC components did not reveal clear differences, except for the AE6 and AE6(u*) formulations (*p* < 0.05). This is likely due to the relatively small differences in group means combined with substantial within‐group variability. Differently, pairwise comparisons between EBC formulations within each PFT revealed significant differences, in particular for SHR|SAV, GRA, DBF|EBF, and ENF (*p* < 0.01). These findings indicate that, for these ecosystems, rigorous quantification of all EBC components is critical. Within each PFT, progressive inclusion of additional AE components consistently increased closure slopes. Differentiating energy contributions between below‐ground and above‐ground heat storage reveals that soil heat storage exerts the strongest effect in short‐canopy ecosystems, with closure improvements ranging from 6% in WET to 20% in CRO and SHR|SAV (compared to 2%–4% in tall‐canopy ecosystems). Conversely, heat storage in air and biomass has greater impact on forest ecosystems, with closure improvements ranging from 7% in MF to 10% in DBF|EBF, reflecting their complex canopy structure and substantial internal energy reservoirs. Open ecosystems show smaller increases (1%–5%) in closure but exhibit larger variability across AE formulations, likely due to simpler structure and less prominent storage terms, yet high heterogeneity in local site characteristics.

Wetland sites maintain the lowest EBC values across AE formulations, with closure improving up to the addition of the air heat storage terms; subsequent inclusion of vegetation‐related components (biomass storage and photosynthesis) does not produce appreciable improvements. This was expected given the hydrological heterogeneities, potentially relevant unmeasured lateral fluxes, and small biomass and photosynthetic contributions that characterize these ecosystems, where turbulent and advective processes contribute more to the residual energy imbalance (Bambach et al. 2022) than vegetation‐mediated storage effects (Butterworth et al. 2024).

### 3.3 EBC Differences Among Networks

**FIGURE 3:**
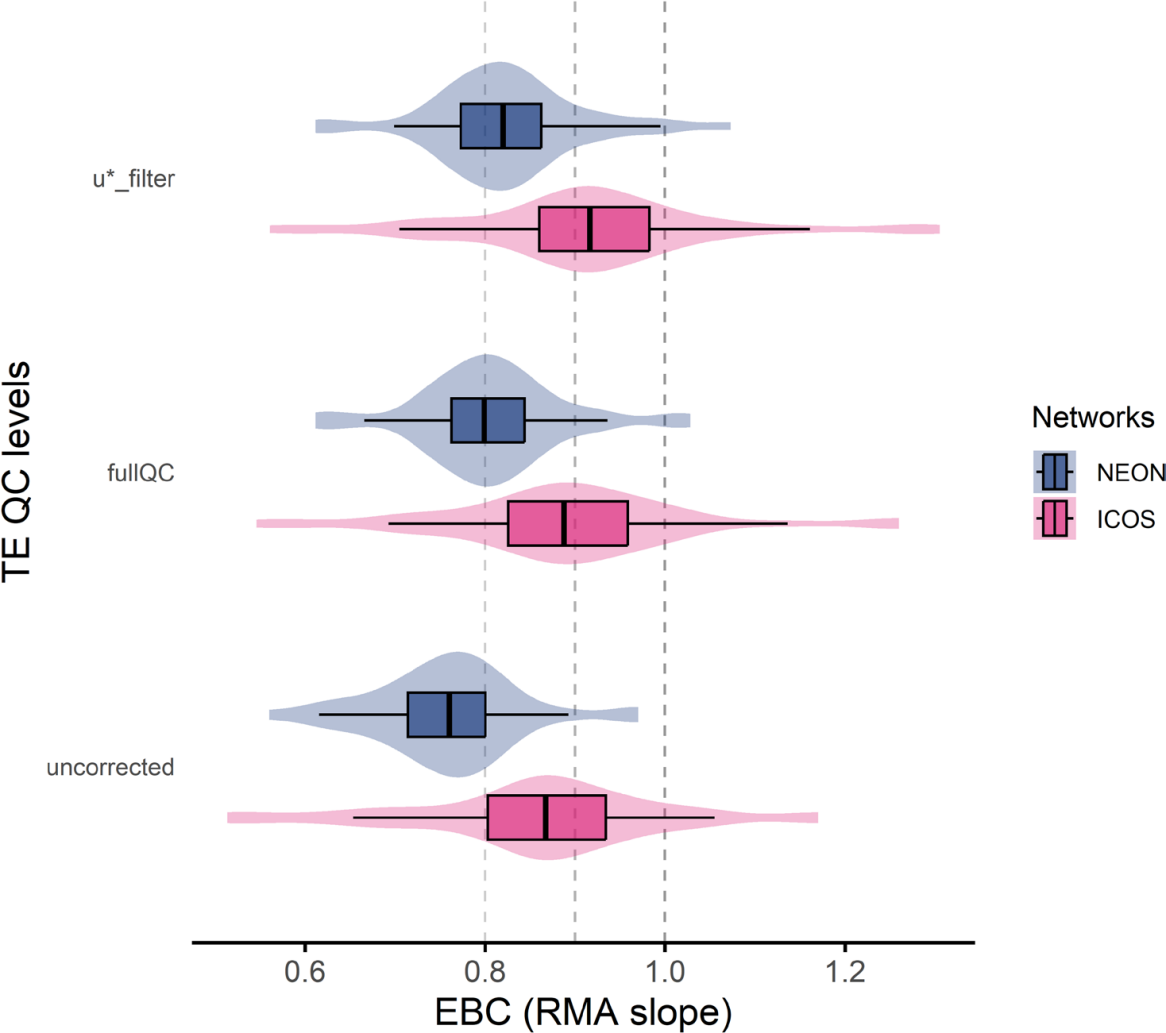
Step improvement of the Energy Balance Closure (EBC) according to the stringency of data quality (QC) filtering by network (increases upward). Closure performances are reported as statistics of the RMA slopes between TE and AE. Here the considered AE combination is the most complete one, AE6 = NETRAD − G − S_G_ − S_H_ − S_LE_ − S_pho_ − S_bio_. TE QC levels: *Uncorrected*, data without comprehensive quality control; *fullQC*, data with full quality‐control; u*_filter, TE further filtered for low turbulence conditions (i.e., AE6(u*), the topmost AE ~ TE pairing in Figures 1 and 2). Vertical dashed lines are for reference, ideal at 1.

Energy balance closure differed between ICOS and NEON stations and varied with the level of TE processing applied (Figure 3). When using TE data without comprehensive quality control (*uncorrected*) and all the components of the AE, ICOS sites exhibited higher closure (95% CI: 0.81–0.91) compared to NEON sites (0.73–0.79), corresponding to about 10% difference in mean EBC. Applying the full quality‐control pipeline (*fullQC*) resulted in larger relative improvements at NEON sites (+6.2%) compared to ICOS sites (+4.0%), indicating the robustness of each network's QA/QC frameworks. When TE was further filtered for low turbulence conditions (u*_filter), the EBC increased at ICOS sites to an average of 0.92 ± 0.02 (+2.4%, 95% CI: 0.87–0.96), while NEON sites reached 0.82 ± 0.01 (+2.2%, 95% CI: 0.80–0.85), remaining consistently lower than ICOS. Differences between networks were significant (Kruskal–Wallis test, α = 0.001).

The systematic offset between networks is notable given that measurement setups are broadly comparable, with the main hardware differences being the model of sonic anemometer deployed and a slightly different rain cup in the gas analyzer intake tube. This suggests that the portion of the discrepancy may come from instrument‐specific characteristics affecting turbulence measurements, in addition to network‐specific data processing protocols. A systematic intercomparison of instrumentation performance and processing pipelines using shared raw datasets is currently underway through a collaborative ICOS‐NEON effort (Durden et al., IN PREP.). Overall, results indicate that applying rigorous data processing can improve the EBC by 4% to 8% (IQR), with a maximum improvement of 16%. Improvement trends were similar across networks, but ICOS consistently shows higher closure across all filtering levels.

### 3.4 EBC At Different Time Scales

**FIGURE 4:**
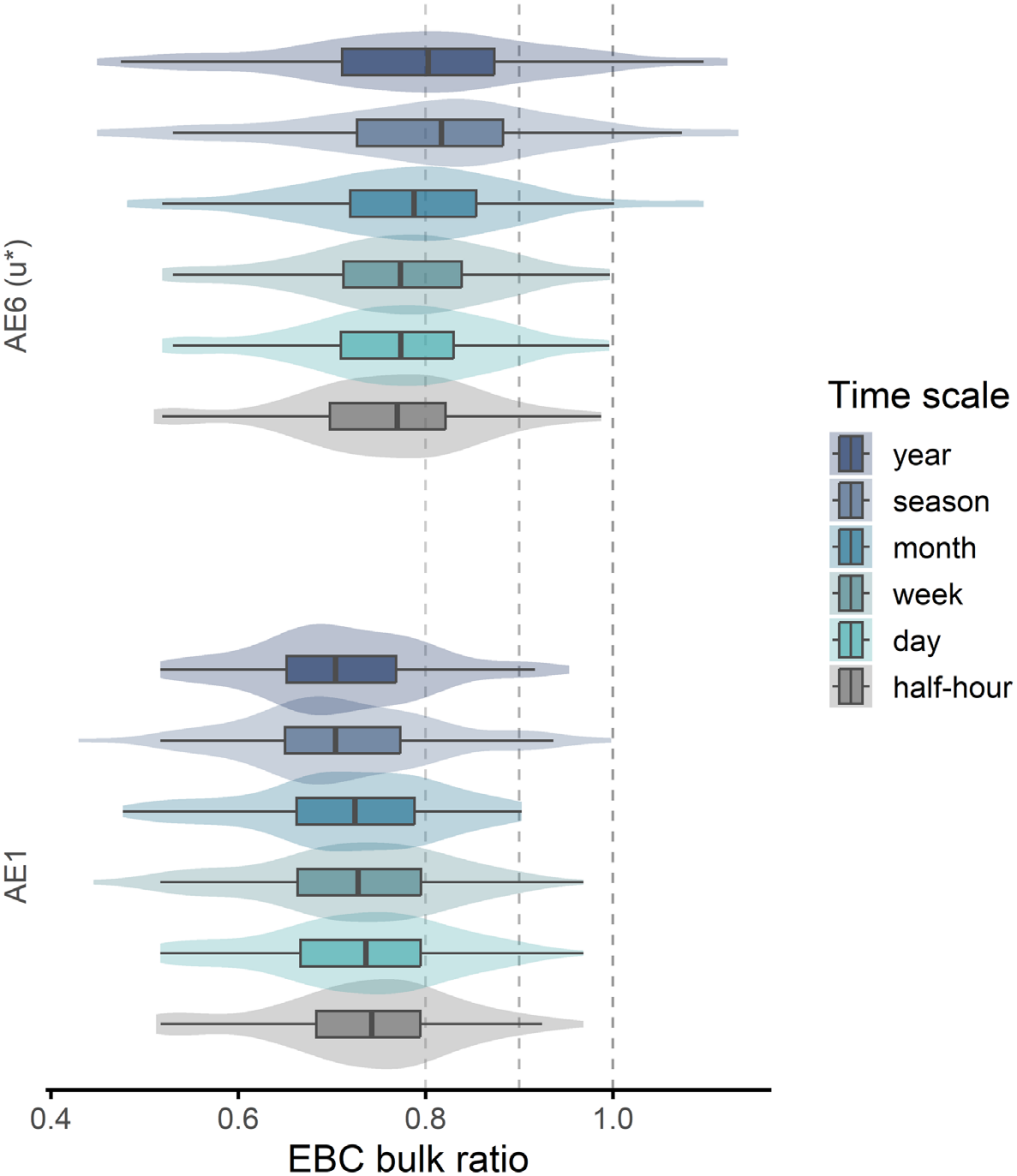
Step changes of the Energy Balance Closure (EBC) according to the temporal aggregation span. EBC is reported as statistics of EBC bulk ratios (∑TE/∑AE using gapfilled half−hourly data). Two EBC components pairings are compared: Full‐QC u* filtered TE (TE_u*_filter) combined with the simpler (AE1 = NETRAD), and the most complete (AE6 = NETRAD−G − S_G_ − S_H_ − S_LE_ − S_pho_ − S_bio_) AE definition (AE6(u*) as in Figures 1 and 2). Within each time scale, outliers were removed for sake of clarity based on the IQR range (discarded if < Q1–1.5*IQR or > Q3 + 1.5*IQR). Vertical dashed lines are for reference, ideal at 1.

To assess the dependence of energy balance closure on temporal aggregation, we examined how the EBC bulk ratio (∑TE/∑AE) evolves across temporal scales, from half‐hourly to annual (Figure 4). In this context, the bulk ratio is preferred over regression‐based EBC metrics, as aggregated TE and AE are gap‐filled and summed over progressively longer intervals, yielding a direct measure of balance agreement (Wilson et al. 2002). Under these conditions, the bulk ratio provides a direct and interpretable measure of energy balance agreement at the integrated temporal scale.

When all components contributing to available energy are accounted for, that is, NETRAD corrected for ground heat flux, soil and air energy storage, and biological and photochemical energy terms (AE6(u*)), the EBC ratio increases monotonically with longer averaging periods, approaching values close to unity at seasonal and annual scales (95% CI: 0.80–0.90 and 0.78–0.87, respectively). This behavior reflects not only a reduction in random variability, but also the progressive attenuation of short‐timescale systematic imbalances—such as non‐stationarity, phase mismatches between TE and AE, unresolved storage and low‐frequency transport—that strongly affect half‐hourly estimates and become less influential when fluxes are temporally integrated (McGloin et al. 2018; Meyers and Hollinger 2004; Oliphant et al. 2004; Twine et al. 2000). Comparable increases in closure at longer intervals have been consistently reported in prior multisite studies (Anderson and Wang 2014; Grachev et al. 2020; Sánchez et al. 2010) confirming that temporal integration mitigates much of the high‐frequency imbalance commonly observed at the half‐hourly scale.

In contrast, when available energy is represented solely by measured net radiation (AE1 = NETRAD), temporal aggregation leads to an opposite trend, with the EBC ratio tending to decrease at coarser time scales. While one would indeed expect the dispersion associated with true storage terms to be reduced when moving from half‐hourly to daily scales due to their partial diurnal cancellation, this expectation only holds if AE is formulated consistently with the underlying energy balance. When storage‐related terms are excluded from AE, their systematic contribution is not removed by temporal integration but instead manifests as a persistent bias. As a result, unresolved canopy heat storage, photosynthetic energy, and air‐column storage introduce a cumulative offset that becomes increasingly evident as fluxes are summed over longer periods, leading to a progressive deterioration of the EBC ratio. This behavior is consistent with previous findings showing that NETRAD alone inadequately represents true AE at diurnal to seasonal scales (Oliphant et al. 2004; Meyers and Hollinger 2004), particularly in forests or tall‐canopy sites where storage processes are non‐negligible (Lindroth et al. 2010; Moderow et al. 2009).

Collectively, these results highlight that temporal aggregation enhances apparent energy balance closure only when AE is represented comprehensively, that is, minor components (S_bio_, S_pho_) are small but not zero, and their inclusion demonstrates that closure improvements are not just a statistical smoothing of temporal aggregation.

### 3.5 Air Storage Flux and Relative Humidity Effects

**FIGURE 5:**
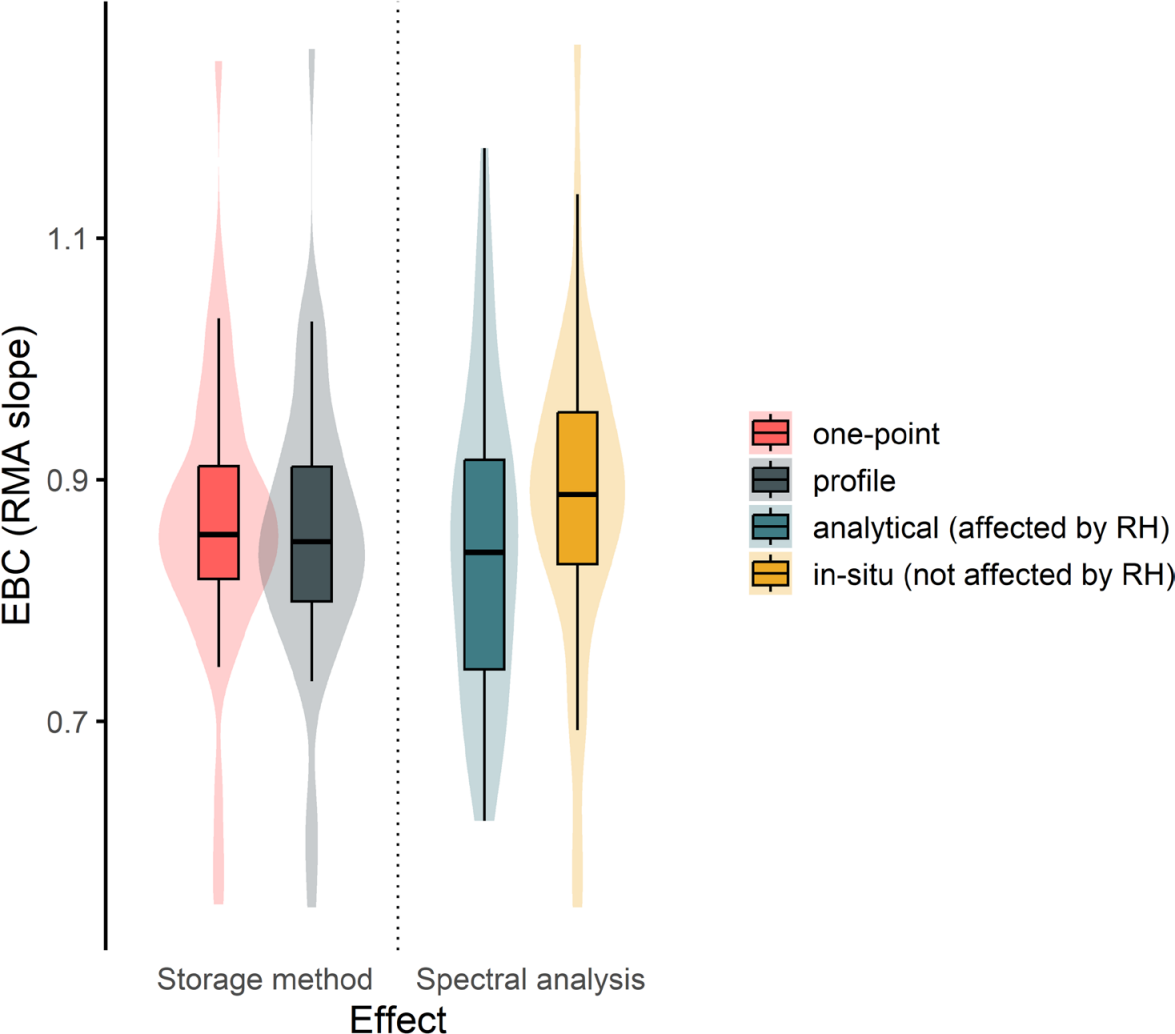
Effects of the air energy storage flux measurement method (left side) and of the spectral correction method for the turbulent energy fluxes (right side) on the energy balance closure (EBC), reported as statistics of the RMA slopes between half‐hourly TE (*TE_fullQC*, full QC applied, no friction velocity filtering) and AE (AE6, full terms accounting) data. As for the storage effect, *one‐point* refers to the single point approximation and *profile* to the temperature and moisture gradient profile measurement method; only forest stations with profile measurements were considered (*n* = 45). As for the spectral analysis effect, *analytical* refers to the analytical method for the energy spectral correction assessment (independent from and impacted by RH, Moncrieff et al. 1997), in situ refers to the data‐driven assessment of the attenuation of spectra (accounts and compensates for the RH effect, Fratini et al. 2012); only stations with concurrent *analytical* and in situ datasets were considered (L1 and L2 data products respectively, only ICOS stations, *n* = 36).

The method used to estimate air‐column storage as well as accounting for air relative humidity in the spectral corrections applied to turbulent fluxes both had surprisingly weak impact on energy balance closure (Figure 5).

Estimating energy storage using the profile method—based on temperature and humidity gradients measured across the air column—resulted in EBC distributions that were statistically indistinguishable from, and not systematically superior to, those obtained using the simpler one‐point approximation (95% CI: 0.82–0.89 and 0.83–0.90, respectively). Although the profile approach is more accurate because it captures vertical structure of temperature and moisture, results show no improvement in energy balance closure when the more complex measurement protocol is used. This outcome suggests that (i) errors in storage estimation are not the dominant contributor to the residual closure at these sites and during these periods, and/or (ii) unaccounted terms and uncertainties in profile measurements (e.g., advective contributions, sensor heights representativeness of respective air layer) partially offset the expected benefit. Note that this finding does not extend to CO_2_, for which the use of profile‐based storage measurements instead of single point estimates has been reported to improve net ecosystem exchange estimates (Nicolini et al. 2018; Xu et al. 2019).

In addition to the air column analysis, we evaluated the sensitivity of EBC to the effect of air relative humidity on energy fluxes attenuation by high frequency damping. The data indicate that applying the in situ, humidity‐sensitive spectral correction improved EBC on average with respect to the analytical RH‐independent correction, although the difference is not statistically significant. In our sample, the in situ approach tended to show higher median slopes (95% CI: 0.84–0.93) than the analytical method (95% CI: 0.80–0.89), in line with Mammarella et al. (2009) who found 1% to 4% EBC improvements when RH‐dependent corrections were applied to LE estimates from closed‐path systems, particularly under high‐humidity conditions. This behavior was consistent across the entire range of RH values, with larger differences for RH > 60% (Supporting Information, Figure S8). This result agrees with expectations from methodological studies showing that inadequate correction of high‐frequency signal losses generally leads to an underestimation of turbulent fluxes (Fratini et al. 2012; Zhang et al. 2023). However, the analytical method tends to substantially underestimate attenuation mainly when the sampling line is long and the tube is not heated due to the strong dependence of H_2_O attenuation on RH. The data used in this analysis originate from the ICOS network, where short and heated inlet tubes are used. This configuration likely partially explains why no statistically significant differences were observed.

### 3.6 Net Radiation Corrections Effects

**FIGURE 6:**
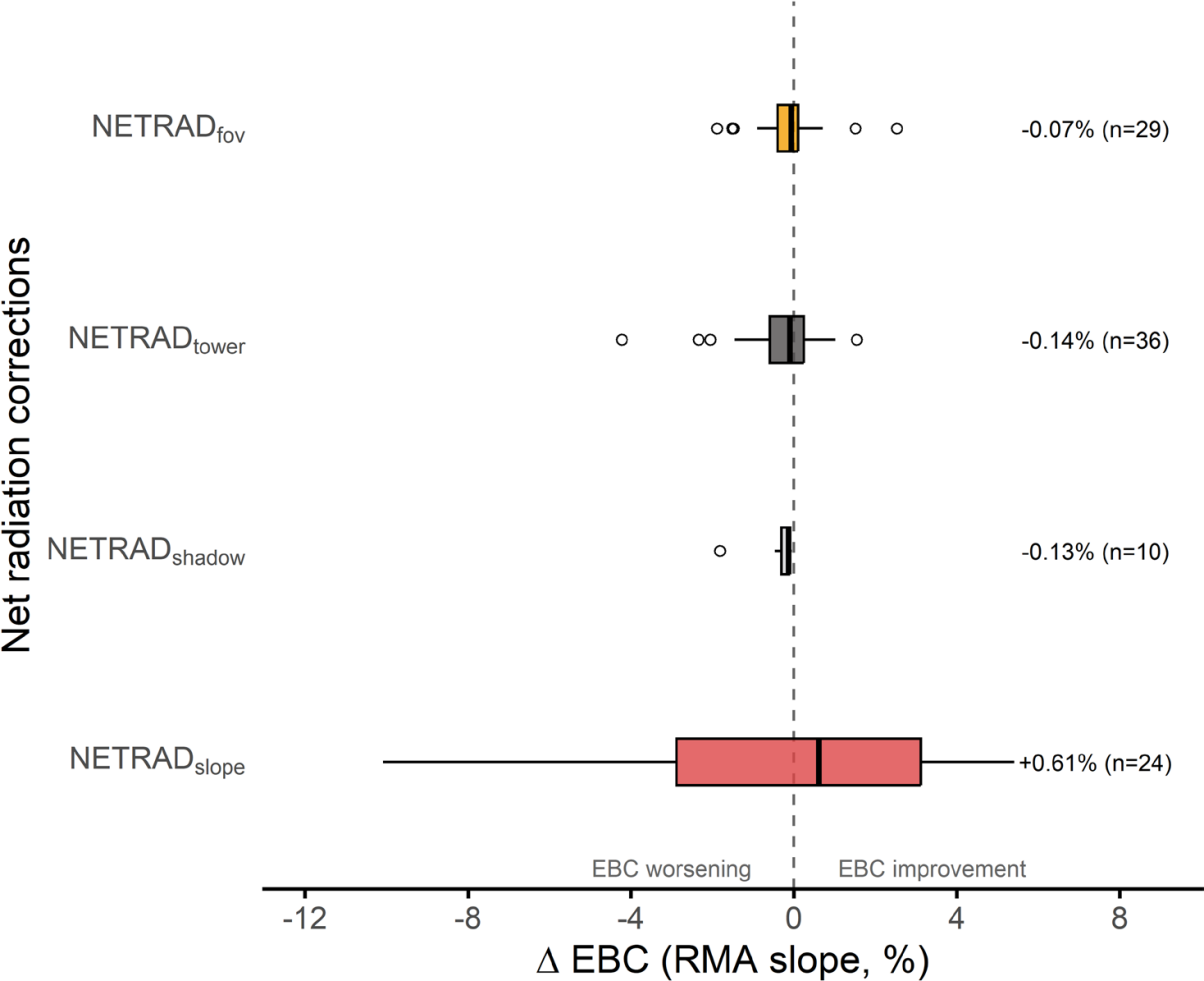
Effect of the net radiation (NETRAD) corrections on the energy balance closure. Boxplots show the distribution of the percentage differences between the EBC RMA slopes at 30 min resolution calculated considering the NETRAD‐corrected versions and the measured NETRAD (baseline) in the available energy compound. The percentages on the right refer to the median change with respect to the benchmark, with the numerosity of the samples between parentheses. NETRAD corrections refer to: Incoming radiation over sloping terrain (NETRAD_slope_, 10 ICOS and 14 NEON stations), shadowing by the surrounding topography (NETRAD_shadow_, 10 ICOS stations), outgoing radiations disturbance by the inclusion of the tower structure in the radiometer field‐of‐view (NETRAD_tower_, 36 ICOS stations), mismatch between radiative sources within the radiometer field‐of‐view and the turbulent fluxes footprint areas (NETRAD_fov_, 29 ICOS stations).

With respect to the benchmark EBC computed using measured radiation (NETRAD), the application of specific NETRAD corrections resulted in small but diverse effects on closure across ICOS sites (Figure 6). Overall, none of the applied corrections substantially altered the EBC statistics, but their relative effectiveness was strongly dependent on‐site characteristics and measurement configurations.

Among the tested corrections, accounting for slope‐induced effects on incoming radiation (NETRAD_slope_) produced the most variable response with a median relative improvement of +0.61% in the EBC slope (ΔEBC), but a wide interquartile range spanning both EBC worsening and improvement (Δ IQR: −3.1%–3.2%, Δ range: −10%–5%). Importantly, this correction was applied only at sites with surface slopes exceeding 2 degrees (*n* = 24), as flatter terrain would be unaffected by such geometric adjustments. The positive median response indicates that slope corrections can partially mitigate underestimation of incoming shortwave radiation on inclined surfaces, yet the large variability reflects how sensitive the adjustment is to local topographic orientation and radiometer alignment. These findings support earlier evidence that while slope corrections can be crucial for representing the radiative energy available for turbulent exchange over mountainous or hilly terrain (Serrano‐Ortiz et al. 2015; Wohlfahrt et al. 2016), they do not universally or consistently resolve the residual energy balance closure gap.

The correction for topographic shadowing (NETRAD_shadow_) showed a weak effect on the EBC in general, with a slightly negative median ΔEBC (Δ IQR: −0.3%–0.1%, though a single case reached −1.8%), consistent with the minimal shading across most ICOS sites.

Tower interference corrections (NETRAD_tower_) slightly worsened energy balance closure (Δ IQR: −0.9%–0.2%). However, at some stations this correction had a more relevant impact (ΔEBC up to −24%, data not shown), resulting in a mean ΔEBC of −1.5%, highlighting the sensitivity of this correction to the specific setup (e.g., tower width, structural pattern density).

Similarly, the correction for field‐of‐view mismatch (NETRAD_fov_) between the radiometer FoV projection and the EC footprint, applied only at sites where the overlap between the two areas was less than 60% of the largest area (*n* = 29), had a negligible overall effect on EBC (median Δ = −0.07%, Δ IQR −0.5%–0.1%), although individual cases reached ±2%–3%. This suggests that, while mismatched footprints can be a relevant source of radiative bias in complex or heterogeneous landscapes, applying this correction does not necessarily improve closure when land‐surface heterogeneity is small relative to the sensor field of view, as a main requirement for ICOS sites.

In conclusion, these results indicate that NETRAD corrections can play a relevant role in a few site‐specific contexts, particularly at inclined or heterogeneous sites, but their overall contribution to the EBC is limited (site‐specific outcomes of NETRAD corrections are reported in the Supporting Information, Figures S9 and S10).

### 3.7 Residual Vegetation‐Related Impacts on EBC

**FIGURE 7:**
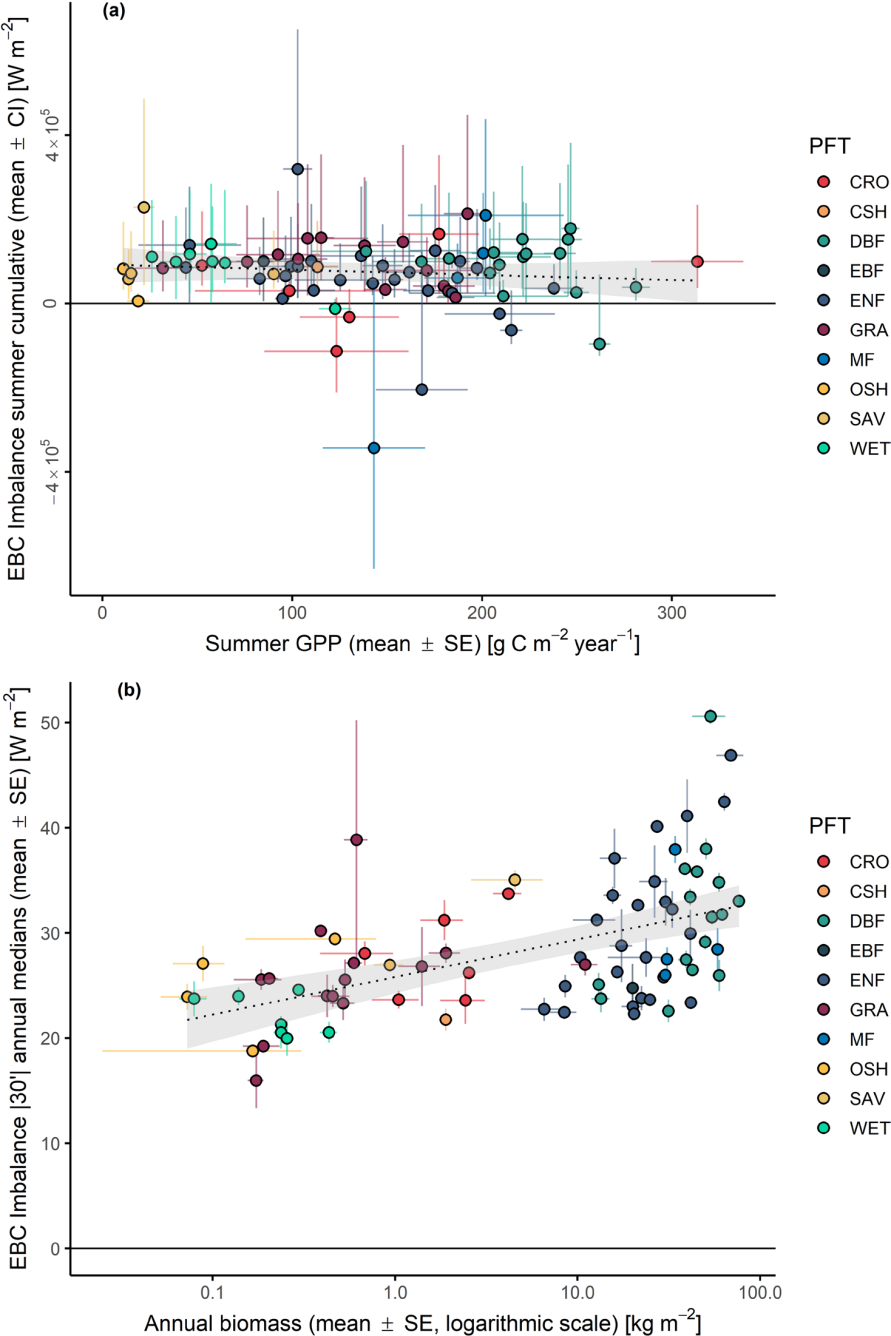
Energy imbalance (EBC Imbalance) in relation to (a) Gross Primary Productivity (GPP) and (b) above‐ground biomass. In (a), the imbalance (W m^−2^) is reported as mean ±95% CI across years of cumulative half‐hourly imbalances calculated during summer (June, July, August) and daytime. The GPP (gC m^−2^ year^−1^) is reported as mean ± SE of annual summertime GPP. The 95% CI was calculated by a non‐parametric bootstrapping of the means of seasonal cumulative imbalances. In (b), the imbalance (W m^−2^) is reported as mean ± SE across years of absolute half‐hourly imbalances annual medians. The biomass (wet biomass, kg m^−2^ year^−1^) is reported as mean ± SE of annual total values, on logarithmic scale.

Although the contribution of photosynthesis and heat storage in the biomass was considered in the analysis, we investigated for potential residual effects due to the relatively simple approach used for the quantification of these two components. Figure 7a shows the relationship between the energy balance closure (EBC) imbalance and gross primary productivity (GPP), considering cumulative half‐hourly imbalances during summer daytime conditions. Across the full range of summertime GPP values (from near zero up to ~300 g C m^−2^ year^−1^), no systematic relationship emerges between ecosystem productivity and the magnitude or sign of the energy imbalance. Mean cumulative imbalances cluster around zero for most sites, and both positive and negative imbalances are observed across the entire GPP range. The absence of a clear trend indicates that, even when restricting the analysis to the period of maximum plant activity (June–August, daytime), the energy imbalance does not scale with photosynthetic uptake. This supports the suitability of the adopted formulation for photosynthetic energy consumption (S_pho_), suggesting that residual imbalance is not primarily driven by short‐term variability in carbon assimilation but is instead linked to other ecosystem properties or unresolved processes.

In contrast, Figure 7b reveals a discernible relationship (*n* = 80, *R* ^*2*^ = 0.3, slope = 0.18 ± 0.03, *p* < 0.001, with a baseline imbalance of 25.0 ± 0.8 W m^−2^ at zero biomass) between the energy imbalance and above‐ground biomass. When the imbalance is expressed as the annual median of absolute half‐hourly values, sites with larger biomass tend to exhibit higher imbalance magnitudes. Median imbalances increase from approximately 20–30 W m^−2^ at low biomass sites (< 10 kg m^−2^) to values exceeding 35–45 W m^−2^ at sites with biomass above 10 kg m^−2^. Notably, this relationship persists despite the explicit correction for biomass heat storage already included in the AE formulation, which itself scales with biomass. This suggests that biomass heat storage is systematically underestimated, or that additional biomass‐linked processes contribute to the residual imbalance. While the limited number of high‐biomass sites increases uncertainty at the upper end of the biomass range, the observed trend points to a non‐negligible role of biomass in shaping the magnitude of the residual energy imbalance beyond the storage terms explicitly considered.

## 4 Discussion

### 4.1 Energy Balance Gap: Insights From Network Data

#### 4.1.1 Progressive Improvement of Energy Balance Closure

Across the 84 analyzed sites, half‐hourly energy balance closure (EBC) improved systematically as progressively more complete definitions of available energy (AE) were adopted. When AE was represented solely by net radiation, mean closure was lowest, averaging at 0.70. Although not representative of the physical processes contributing to the energy balance, this value is in line with values commonly reported in literature (e.g., Wilson et al. 2002; Foken 2008). Sequential inclusion of soil heat flux, soil heat storage, air‐column storage, and biological energy terms resulted in cumulative improvements of ~16% on average, with site‐specific gains reaching up to 40%. The largest single improvement followed the inclusion of soil heat storage (+6.8%), emphasizing the critical role of belowground heat dynamics, particularly at sites with strong diurnal soil temperature gradients. This highlights the importance of measuring ground heat flux at multiple locations representative of spatial variability within the flux footprint. Smaller but consistent gains were associated with air‐column storage (+2.1%), photosynthetic energy consumption (+2.3%), and biomass heat storage (+4.0%). The latter increased mean closure from 0.82 to 0.85, demonstrating that vegetation represents a non‐negligible energy reservoir, especially in tall or structurally complex canopies (Figure 2). The highest closure (0.84–0.90) was achieved when the most complete AE formulation was combined with fully quality controlled and friction‐velocity filtered turbulent fluxes. Although unity was not consistently reached, the progressive convergence toward the 1:1 line indicates that neglected storage terms account for a substantial fraction of the EBC gap, beyond random measurement error alone. This also highlights the need for continued methodological improvements in the estimation of storage components, particularly biomass heat storage and soil heat flux.

Although there is a general network‐level improvement, site‐specific differences are present. A subset of sites exhibited poorer closure when progressively more comprehensive AE formulations were applied: 1.2% when moving from AE1 to AE2, 2.4% (AE2 → AE3), 10.7% (AE3 → AE4), 0% (AE4 → AE5), 1.3% (AE5 → AE6), and 1.3% (AE6 → AE6(u*)). The largest worsening occurred when including biomass heat storage (AE3 → AE4), suggesting that measurement artifacts, spatial representativeness issues, or energy allocation assumptions are more critical for this energetic compound. Furthermore, comprehensive AE formulations increased the prevalence of energy balance overclosure (EBC > 1.0), with the proportion of sites exceeding unity rising from ~2% for AE = NETRAD to ~10% for the most complete formulations (AE6 or AE6(u*)).

Individual sites exhibiting overclosure do not necessarily indicate measurement error, as random uncertainties in both turbulent fluxes and available energy naturally produce scatter around the 1:1 line. However, systematic overclosure patterns may indicate overestimation of available energy, underestimation of unmeasured sinks (e.g., advection, drainage), or compounding biases across terms. These responses underscore that while more comprehensive AE formulations universally provide more physically accurate closure estimates, the magnitude of improvement from including additional energy storage terms varies substantially across sites. Storage terms should be included whenever physically relevant as their contribution scales with the actual magnitude of each energy compound, rather than being optional. For instance, biomass heat storage substantially improved closure at forested sites with high biomass and strong canopy temperature gradients, where this term is physically large, but contributed minimal corrections at grassland or cropland sites where aboveground biomass, and thus storage capacity, is naturally small. This highlights the importance of site‐specific evaluation of the most comprehensive AE formulation.

#### 4.1.2 Ecosystem Role on Closure Efficiency

EBC varied systematically among plant functional types (PFTs), reflecting ecosystem structure/phenology and energy storage capacity. When including all the components of AE, short‐canopy ecosystems (wetlands, grasslands, shrublands) exhibited lower closure (0.77–0.84) at the half‐hourly scale, whereas tall‐canopy ecosystems clustered around 0.90. Croplands showed the highest mean closure (0.96), consistent with their relatively homogeneous surfaces, limited vertical complexity, and low biomass storage. Forested ecosystems exhibited the strongest sensitivity to AE completeness, with marked improvements following the inclusion of biomass and air‐column storage terms. This behavior reflects their larger heat capacity and multilayer canopy structure (Oliphant et al. 2004; Moderow et al. 2009; Lindroth et al. 2010). Wetlands exhibited lower EBC across different AE formulations, reflecting the combined effects of physical complexity and methodological limitations. Beyond the inherent physical challenges, including lateral water and energy fluxes, high spatial heterogeneity in hydrological conditions, unmeasured water layer heat storage above the soil surface, and relatively small contributions from biomass heat storage and photosynthetic energy fixation, measurement uncertainties and representativeness issues critically constrain EBC quantification in these ecosystems (Rouse 2000; Rinne et al. 2025). Water heat storage, in particular, can represent a substantial energy reservoir, but direct quantification requires water temperature profile measurements that were unavailable at our sites, representing a priority for future site‐specific investigations. These challenges are particularly important for variables related to soil–water–atmosphere exchanges, where sensor placement, footprint mismatch, and phase‐shifts complicate the energy partitioning.

These results confirm that ecosystem‐specific processes modulate closure efficiency and that rigorous AE accounting is particularly critical in structurally complex systems.

#### 4.1.3 Network‐Related Differences and Processing Effects

Systematic differences in EBC emerged between ICOS and NEON sites, with ICOS consistently exhibiting higher closure across all turbulent energy (TE) processing levels. The largest offset (~10%) occurred for uncorrected TE, while deep quality control and u* filtering reduced but did not eliminate it. This suggests that network‐specific processing pipelines manage to filter low‐quality fluxes improving EBC; however, residual differences persist even after stringent quality control. Part of this offset may also reflect differences in the distribution of ecosystem types between networks, with certain ecosystems exhibiting inherently better or worse closure (see Supporting Information Figure S11 for ecosystem‐network breakdown). Also, ICOS sites displayed greater inter‐site EBC variability than NEON (Figure 2, wider boxplot distributions, especially near or above unity). This likely reflects operational differences: ICOS relies on decentralized station teams managing the application of the standard protocols, whereas NEON employs centralized field operations with standardized technician teams. For this reason, in ICOS a greater heterogeneity in protocol application, measurement practices, and on‐site decision‐making is expected that can propagate into flux uncertainty distributions. Nonetheless, given the broadly similar ecosystem representation and measurement protocols, the remaining systematic offset likely reflects a combination of instrumental hardware differences and data processing algorithms.

##### 4.1.3.1 Instrumental Hardware Contributions

The two networks employ different core instrumentation, which introduces potential systematic biases. ICOS sites use the Gill HS‐50/100, whereas NEON relies on the Campbell Scientific CSAT3 (or CSAT3B) anemometers. Field intercomparison experiments have documented non‐negligible differences between sonic models, particularly under high wind speeds, low temperatures, or precipitation events (Mauder and Zeeman 2018). Firmware settings, including coordinate system definitions, digital filtering, and spike detection thresholds, can further impact measured turbulence statistics, though these effects are not systematically quantified across networks. The CSAT3's well‐documented flow distortion issues in certain wind sectors (Horst et al. 2015) may also contribute differentially depending on dominant wind patterns at NEON versus ICOS sites.

Both networks primarily rely on enclosed‐path infrared gas analyzers (IRGA), but tube configuration differs: ICOS sites use LI‐7200 with fixed tube lengths (0.71 m) and heating strategies, NEON still employs the LI‐7200 yet with fixed heated tube lengths (0.72 m), but a different rain cap at the intake designed to minimize spectral attenuation (Metzger et al. 2016). Tube length, heating rate, and inline filter characteristics critically affect high‐frequency attenuation of water vapor and CO_2_ signals (Ibrom et al. 2007). Smidt et al. (2025) demonstrated that inadequate heating of LI‐7200 intake tubes under humid conditions can induce substantial latent heat flux underestimation—up to 10%–15% in extreme cases—which is partially but not fully recovered by standard spectral correction algorithms. Filter mesh size, possible filter fouling and its placement further affect attenuation, with finer filters providing cleaner signals but at the cost of increased damping (Peltola et al. 2021).

##### 4.1.3.2 Data Processing and Spectral Correction Differences

Beyond hardware, differences in flux computation algorithms could contribute to the observed systematic offsets. In particular, ICOS Level 2 processing applies an empirical (in situ) spectral correction approach that adapts to site‐specific attenuation characteristics, whereas NEON employs a wavelet‐based approach to high‐frequency spectral corrections which directly corrects the high‐frequency data instead of the co‐spectrum and corrects each individual averaging period (Nordbo and Katul 2012; Metzger et al. 2022). The wavelet‐based approach has some limitations when the spectra are noisy, which can result in default spectral corrections that could underestimate attenuation. Time‐lag estimation between vertical wind speed and scalar concentrations differs between processing pipelines. ICOS software dynamically optimizes lag windows using covariance maximization with stability‐dependent constraints, whereas NEON uses a maximization of the cross‐correlation in conjunction with applying a high‐pass filtering to reduce noise and maximize signal strength (Metzger et al. 2022). Peltola et al. (2021) showed that suboptimal lag estimation, particularly under low‐turbulence conditions, can cause 3%–5% flux underestimation due to incomplete alignment of turbulent structures. This effect aggregates with high‐frequency loss corrections, which rely on accurate lag specification.

While both networks implement stationarity and integral turbulence characteristic (ITC) tests following Foken and Wichura (1996), their QC frameworks differ substantially. ICOS employs an advanced, statistically robust QC system combining traditional turbulence tests with the comprehensive data cleaning procedure described in Vitale et al. (2020). This procedure integrates multiple statistical tests aimed at detecting specific sources of systematic error (including instrumental malfunctions, violation of EC assumptions, and aberrant flux values) through a flexible, scalable framework. The approach is fully data‐driven and avoids subjective decision rules, making it particularly suitable for centralized processing pipelines where reproducibility and high‐quality standards are essential. ICOS flags are hierarchically inherited: flux quality flags combine results from constituent variables (e.g., vertical wind speed, sonic temperature for H; vertical wind and H_2_O for LE) with turbulence characteristic tests, producing a final quality assessment where flag = 2 indicates severe quality issues warranting data removal.

NEON implements a QC strategy based on the eddy4R processing framework (Metzger et al. 2017) that is in line with NEON quality assurance and quality control framework (Smith et al. 2014). NEON quality flag thresholds are more lenient than ICOS criteria, resulting in higher data retention but potentially lower flux quality. Flux quality flags similarly inherit assessments from input variables and are combined with stationarity and ITC tests. However, NEON's QC emphasizes operational robustness and data coverage across a continent‐scale heterogeneous network, accepting somewhat greater variability in data quality to maximize spatial and temporal coverage. When full QC filtering is applied, NEON sites experience more substantial closure efficiency improvement than ICOS sites, narrowing but not eliminating the network‐level closure offset. Variability in the EBC across NEON sites is well‐bounded, indicating sufficient quality control and suggesting some other source of consistent bias. It is important to note, however, that overly conservative filtering can potentially bias retained data toward ideal atmospheric conditions, potentially masking systematic instrumental issues that manifest primarily under challenging meteorological conditions (high humidity, low turbulence, temperature extremes). This trade‐off between data coverage and data quality represents a fundamental challenge in network flux monitoring.

#### 4.1.4 Temporal Aggregation and AE Completeness

When all storage and biological terms were included, the EBC bulk ratio (∑TE/∑AE) increased monotonically from half‐hourly to seasonal and annual scales. In contrast, when AE was approximated by NETRAD alone, aggregation led to closure worsening at longer timescales (Figure 4). This indicates that unaccounted storage terms introduce systematic biases that accumulate rather than average out. Similar limitations of AE formulations at daily to seasonal scales have been reported previously, particularly in forested ecosystems (Meyers and Hollinger 2004; Oliphant et al. 2004). These results demonstrate that improved long‐term closure depends critically on comprehensive AE representation. Relying solely on NETRAD as a proxy for AE can misrepresent long‐term energy partitioning and produce progressively biased EBC ratios as the integration window increases. The divergence between two analyzed AE formulations (net radiation only, and full terms inclusion) underscores the necessity of including all relevant storage components in AE when evaluating energy balance closure across multiple temporal scales. The potential influence of gap‐filled data on EBC estimates was evaluated through a sensitivity analysis based on the fraction of gap‐filled observations (see Supporting Information Figure S7). Across sites, no systematic relationship emerged between the overall fraction of gap‐filled data and the resulting EBC values across temporal aggregation scales. When considering only gaps in turbulent fluxes, a slight tendency toward lower EBC ratios was observed at higher gap fractions, although the distributions remained largely overlapping. These results suggest that, within the range of conditions represented in the analyzed datasets, the amount of gap‐filled data does not exert a dominant control over long‐term EBC statistics.

#### 4.1.5 Limited Impact of Air Storage Method, Air Relative Humidity, and Net Radiation Corrections

Neither the use of profile‐based air storage measurements nor the application of humidity‐sensitive spectral corrections produced statistically significant improvements in EBC. While more accurate with respect to the one‐point approximation, profile methods may still be affected by representativeness and uncertainties also linked to advective fluxes. The limited impact of the humidity‐sensitive spectral correction observed here likely reflects the use of short, heated sampling lines that effectively minimize high‐frequency attenuation, rather than indicating a negligible role of relative humidity in shaping energy balance closure.

Similarly, corrections applied to the net radiation (due to slope/aspect, shadowing, tower interference, and sensors' footprint mismatch) yielded small and site‐specific effects that largely canceled out at network scale. While such corrections can be essential at individual sites—particularly in complex terrain—they do not resolve the persistent residual imbalance when considered collectively, and in some cases also lead to worsening the EB closure.

#### 4.1.6 Role of Vegetation Activity on the Residual Energy Imbalance

The absence of a clear relationship among residual energy imbalance and plants gross primary productivity (GPP) indicates that photosynthesis itself is unlikely to be a dominant driver of the residual imbalance at the temporal scales considered. In turn, it supports the adequacy of the adopted approach to account for photosynthetic energy consumption within the available energy formulation, indicating that this term effectively captures the energetic cost of carbon assimilation. Our formulation (Equation 5) only accounts for the chemical energy stored in reduced carbon at the molar level. A key issue is how to consistently partition stored chemical energy, metabolic expenditure, and subsequent heat dissipation. Incorporating the full biochemical energy costs (e.g., ATP and NADPH turnover) at half‐hourly resolution would require a more detailed mechanistic representation of intracellular pathways (Gu et al. 2019) and may introduce methodological challenges, including potential double counting with sensible and storage heat fluxes and phase shifts between radiation absorption and respiratory heat release. For these reasons, we adopted a conservative but thermodynamically consistent approach that accounts for the chemical energy stored in fixed carbon. While a more detailed treatment could yield some improvement in energy balance closure, this remains a topic for future investigation. Any remaining imbalance appears to be decoupled from short‐term variations in photosynthetic activity and is more likely associated with other ecosystem properties or processes, such as biomass‐related heat storage, canopy structure, or unresolved transport mechanisms. While more detailed analyses would be required to assess potential additional effects, our results suggest that residual errors in the representation of photosynthetic energy consumption are not a critical contributor to the observed energy balance nonclosure.

On the other hand, the observed association between energy imbalance and above‐ground biomass suggests that biomass‐related processes may influence the magnitude of the residual nonclosure (although the relationship is characterized by considerable scatter). Its persistence after explicitly accounting for biomass heat storage indicates that the imbalance may also reflect systematic effects linked to ecosystem structure. This pattern may point to limitations in the current parameterization of biomass heat storage, which likely accounts for partial contributions only. It is worth noting that using air temperature as a proxy for biomass temperature can underestimate true canopy temperature dynamics, particularly under unstable daytime conditions. Any resulting bias would underestimate biomass heat storage, thereby reinforcing our conclusion that residual imbalances may reflect unmeasured biomass‐related processes. This underscores the importance of direct canopy temperature measurements for comprehensive energy balance assessment.

At the same time, biomass covaries with other ecosystem attributes, including canopy height and aerodynamic complexity, making it difficult to isolate its direct role. Indeed, the emerging relationship between residual energy imbalance and ecosystem structural attributes such as biomass may reflect not only incomplete accounting of storage terms, but also the increasing role of surface heterogeneity in organizing large coherent turbulent motions, which can bias eddy covariance flux measurements and contribute to persistent nonclosure.

Overall, these results suggest that biomass‐related effects warrant further investigation, through targeted measurements, to better constrain their contribution to the remaining energy balance nonclosure.

### 4.2 Toward a Bounded Interpretation of Energy Balance Closure

Our analysis shows that systematically expanding the definition of the available energy term to include soil, air, biomass, and biological heat storage leads to consistent and non‐trivial improvements in EBC across ecosystems, confirming that a substantial, yet not complete, portion of the apparent imbalance is methodological rather than physical. This finding reinforces the need for harmonized, comprehensive AE formulations as a baseline requirement in EC studies. However, even under optimal conditions—standardized measurements, rigorous quality control, and comprehensive energy accounting—closure rarely reaches unity at half‐hourly scales. The persistent residual, resistant to methodological corrections, likely originates from canopy/sub‐canopy heterogeneities, advective processes, and mesoscale circulations operating beyond the tower‐based EC systems reference volume. The application of the u* filtering to remove periods affected by low turbulence and potential advective fluxes improves in general the EBC (+2.3% on average), suggesting the importance of its application also to LE and H. The u* filtering criterion targets conditions where low turbulent mixing allows non‐negligible advective fluxes and thermal decoupling events. Filtering removed between 7% and 63% of half‐hourly data across sites, reflecting differences in canopy structure, surface roughness, and nocturnal stratification characteristics. Critically, the percentage of data removed by u* filtering showed no systematic relationship with energy balance closure quality (see Figure S6), supporting that filtering removes systematically biased measurements rather than reducing valuable information.

Network‐level differences, particularly between ICOS and NEON, further illustrate that instrumental choices and processing pipelines can systematically bias closure estimates, even within otherwise harmonized frameworks. Rather than seeking a single “best” correction, these differences highlight the importance of transparency, traceability, and uncertainty bounds in comparative studies.

#### 4.2.1 Implications Beyond Micrometeorology

Although energy balance closure (EBC) is traditionally treated as a micrometeorological diagnostic, its implications extend beyond turbulence measurements. From both modeling and carbon‐accounting perspectives, treating EBC purely as a technical correction problem risks masking structurally unresolved processes. Closure‐based adjustments of energy fluxes (e.g., Charuchittipan et al. 2014; Twine et al. 2000), can lead to artificial agreement between observations and models if applied without explicitly acknowledging residual imbalance, weakening rather than strengthening inference. In contrast, explicitly bounding the irreducible component of the energy imbalance enhances the transparency, interpretability, and robustness of EC datasets, particularly when used for model evaluation, data assimilation, or upscaling applications.

## 5 Conclusions and Outlook

### 5.1 Key Findings

Overall, these findings demonstrate that substantial improvements in energy balance closure are achieved through comprehensive accounting of available energy and rigorous filtering of turbulent energy. The observed residual imbalance may reflect unresolved turbulent transport processes, such as boundary layer entrainment and heterogeneity‐driven large eddies, that are not fully captured by conventional eddy covariance measurements and require further investigation for proper quantification.

Increasing standardization of measurement hardware and data processing, combined with large‐scale, multisite analyses, now enables a robust quantification of the residual energy balance nonclosure. This, in turn, provides a solid basis to move beyond descriptive assessments and to critically evaluate which physical and biological processes are most likely responsible for the remaining imbalance.

No single correction resolves the energy gap, but their cumulative application significantly narrows it and clarifies its physical origins, supporting a more robust interpretation of eddy‐covariance energy fluxes across ecosystems and time scales.

### 5.2 Final Remarks and Future Directions

Based on the evidence synthesized here, we propose that energy balance closure should be treated as a diagnostic constraint rather than an optimization target. This shift is critical to avoid compensating unresolved physical processes with methodological adjustments. This implies: (1) accounting for all storage terms in the energy balance; (2) using EBC metrics as indicators of data quality rather than acceptance criteria; and (3) explicitly reporting residual imbalance ranges as part of uncertainty characterization.

By reframing energy balance closure as a bounded and interpretable diagnostic rather than an error to be eliminated, this perspective provides both a critical warning and a practical pathway for the robust use of EC data across Earth system science. It highlights that the observed energy imbalance largely arises from methodological limitations in how available energy and turbulent fluxes are quantified, while a residual reflects physical processes that are not fully resolved by current observational approaches.

Looking forward, tower‐based EC measurements should be recognized as a foundational but spatially constrained EBC diagnostic rather than a complete energy balance solution. Meaningful progress requires integrating EC observations with spatially explicit methods (e.g., remote sensing, landscape‐scale experiments) to explicitly address advection, surface heterogeneity, and the scale mismatches inherent to single‐point measurements.

Embracing this bounded uncertainty framework strengthens the scientific rigor of flux‐based research and ensures more transparent communication of EC data limitations to the broader modeling and policy communities.

## Author Contributions

**Giacomo Nicolini:** conceptualization, data curation, formal analysis, methodology, software, writing – original draft. **David Durden:** data curation, investigation, writing – review and editing. **Luca Di Fiore:** data curation, methodology, writing – review and editing. **Christopher Florian:** data curation, writing – review and editing. **Simone Sabbatini:** methodology, writing – review and editing. **Bert Gielen:** data curation, writing – review and editing. **Arne Iserbyt:** data curation. **Benjamin Loubet:** data curation, writing – review and editing. **Ivan Mammarella:** data curation, writing – review and editing. **Adriana Mariotti:** data curation. **Maarten Op de Beeck:** data curation, writing – review and editing. **Caleb Slemmons:** data curation, writing – review and editing. **Carlo Trotta:** data curation. **Adam Young:** data curation, writing – review and editing. **Abad Chabbi:** writing – review and editing. **Iris Feigenwinter:** data curation, writing – review and editing. **Bernard Heinesch:** data curation, writing – review and editing. **Natalia Kowalska:** data curation, writing – review and editing. **Matthias Mauder:** data curation, writing – review and editing. **Ladislav Šigut:** data curation, writing – review and editing. **Michiel van der Molen:** data curation, writing – review and editing. **Flavio Bastos Campos:** data curation, writing – review and editing. **Daniel Berveiller:** data curation, writing – review and editing. **Christian Brümmer:** data curation, writing – review and editing. **Matthias Cuntz:** data curation, writing – review and editing. **Jean‐Christophe Domec:** data curation, writing – review and editing. **Benjamin Dumont:** data curation, writing – review and editing. **Silvano Fares:** data curation, writing – review and editing. **Damiano Gianelle:** data curation, writing – review and editing. **Rasmus Jensen:** data curation, writing – review and editing. **Carmen Kalalian:** data curation, writing – review and editing. **Natascha Kljun:** data curation, writing – review and editing. **Holger Lange:** data curation, writing – review and editing. **Jean‐Marc Limousin:** data curation, writing – review and editing. **Erik Lundin:** data curation, writing – review and editing. **Antonio Manco:** data curation, writing – review and editing. **Leonardo Montagnani:** data curation, writing – review and editing. **Eiko Nemitz:** data curation, writing – review and editing. **Matthias Peichl:** data curation, writing – review and editing. **Erkka Rinne:** data curation, writing – review and editing. **Marilyn Roland:** data curation, writing – review and editing. **Marius Schmidt:** data curation, writing – review and editing. **Guillaume Simioni:** data curation, writing – review and editing. **Abin Thomas:** data curation, writing – review and editing. **Caroline Vincke:** data curation, writing – review and editing. **Dario Papale:** conceptualization, funding acquisition, methodology, project administration, supervision, validation, writing – review and editing.

## Conflicts of Interest

The authors declare no conflicts of interest.

## Supporting information


**Figure S1:** Map of the 84 stations included in the analysis, covering a wide range of terrestrial ecosystems (IGBP, named as plant functional types (PFT) in the main text). Thirty‐eight stations belong to the ICOS network and 46 stations to the NEON network.
**Table S1:** Vegetation cover (%) and leaf area index (LAI) stats at the analyzed stations. ICOS stations, assessed for 2024: vegetation cover was calculated using Sentinel‐2 Enhanced Vegetation Index (EVI; range: −1 to +1, with active vegetation typically > 0.20), pixels with EVI ≥ 0.25 were classified as vegetated; LAI is measured indirectly at forest sites with digital hemispherical photography (DHP) or a linear ceptometer, depending on the seasonal max value, at nonforest sites, LAI is measured with a ceptometer. NEON stations: vegetation covers were not available; LAI values were derived from MODIS imagery: LAI values were extracted for a 1 km radius surrounding each tower for 2022–2023 then the average was taken across the pixels to generate site‑level LAI, and then seasonal means for winter and summer were computed.
**Figure S2:** Vegetation cover maps at analyzed ICOS stations. Green and grey pixels indicate vegetated and nonvegetated areas, respectively. The EC flux footprint climatology (80% cumulative contribution, red contours) defines the reference surface for the analysis. Vegetation cover was assessed for 2024 using Sentinel‐2 Enhanced Vegetation Index (EVI; range: −1 to +1, with active vegetation typically > 0.20). Pixels with EVI ≥ 0.25 were classified as vegetated, and annual medians computed accordingly. Red numbers in the bottom‐right corner indicate the fractional vegetation cover (%) within the EC flux footprint. Low vegetation cover values at SE‐Nor (ENF) are due to a clear‐cutting event in 2022–2023.
**Table S2:** Species composition at crop stations.
**Figure S3:** Distribution of energy balance closure (EBC) at half‐hour resolution as estimated by OLS (ordinary linear) and RMA (reduced major axis) slopes for each AE/TE pairing (same as Figures 1 and 2 of the main text: AE1 = NETRAD; AE2 = NETRAD − G; AE3 = NETRAD − G − SG; AE4 = NETRAD − G − SG − SH − SLE; AE5 = AE4 − Spho; AE6 = AE5 − Sbio; AE6(u*) = AE6 with u*‐filtered TE). Boxplots show the median and interquartile range, while colors distinguish the regression method.
**Figure S4:** Sensitivity of energy balance closure (EBC) to spatial sampling of storage corrected soil heat flux (G + SG). The plot shows the change in EBC RMA slopes (H + LE ~ AE) relative to the reference configuration (AE3 = NETRAD − G − SG, used in the main analysis) when different combinations of soil heat flux sensors are used to estimate G. Only combinations including three or four sensors were considered. Each point represents a different sensor combination at a given site. Point colors indicate fractional vegetation cover derived from remote sensing. Numbers on the bottom side represent the used combinations: 3 means all the permutations with 3 sensors (for stations with 4 measurement points), 3–4 means all the permutations with 3 and 4 sensors (for stations with 5 measurement points).
**Figure S5:** Relationship between site‐specific u* threshold values and percentage of data removed by filtering. Points are colored by ecosystem type.
**Table S3:** Friction velocity (u*) filtering assessment. PFT is the plant functional types, u* thrs is the site‐specific calculated u* threshold for data filtering, u* filter 24H, u* filter NT, and u* filter DT are the amount of data discarded (%) because of the u* filtering for the whole period, nighttime and daytime data respectively, EBC_RMA is the energy balance closure computed at half‐hourly scale (RMA slope, %), EBC_BR is the energy balance closure computed at monthly scale (bulk ratio, %).
**Figure S6:** Energy balance closure (EBC) as a function of the percentage of data removed by u* filtering, by temporal aggregation scale, a. 30 min (EBC as RMA slope), and b. monthly resolution (EBC as bulk ratio).
**Figure S7:** Influence of gap‐filled data on energy balance closure (EBC) across temporal aggregation scales. Violin plots show the overall distribution of the EBC bulk ratio for each time scale. Boxplots represent sites grouped into terciles of gap fraction. Panel (a) shows the overall gap fraction considering all variables entering the energy balance, while panel (b) shows the gap fraction considering only turbulent fluxes. Gap classes are arranged from lower to higher gap fractions from bottom to top within each violin. Vertical dashed lines indicate reference closure thresholds.
**Figure S8:** Sensitivity of the energy balance closure (EBC, reported here as RMA slopes at half hourly time scale) to the effect of air relative humidity (RH) on energy fluxes attenuation.
**Figure S9a:** ICOS stations: BE‐Bra—FR‐Fon. Site‐level mean diurnal cycles of correction‐induced differences in net radiation (ΔNETRAD = NETRAD_corr − NETRAD). For each site and correction (slope, topographic shadowing, tower disturbance, and field‐of‐view/footprint effects), half‐hourly means were calculated across 2024. Shaded bands indicate the standard error of the mean. Each panel uses an independent y‐axis scale to emphasize intra‐site dynamics.
**Figure S9b:** ICOS stations: FR‐Gri—UK‐AMo. Site‐level mean diurnal cycles of correction‐induced differences in net radiation. See the caption of Figure S9 a (above).
**Figure S10:** Aerial maps of ICOS stations comparing the EC flux footprint climatology (80% cumulative contribution ECFoV), with the surface projection of the pyrgeometers (LWradFoV) and pyranometer (SWradFoV) field of view.
**Table S4a:** EBC comparison among PFT, all EBC formulations included (AE1‐AE6(u*)). Wilcoxon rank sum test: pairwise comparisons between group levels with corrections for multiple testing. Statistically significant difference if *p* < 0.05 (values in bold).
**Table S4b:** EBC comparison among PFT, only AE6(u*) EBC formulation. Wilcoxon rank sum test: pairwise comparisons between group levels with corrections for multiple testing. Statistically significant difference if *p* < 0.05 (values in bold).
**Figure S11a:** Distribution of EBC (RMA slopes at half‐hourly time scale) for each forest ecosystem plant functional type (PFT), separated by network. The number of sites per PFT and network is indicated below each group (n). PFTs are: deciduous broadleaf forests and evergreen broadleaf forests (DBF|EBF), evergreen needleleaf forests (ENF), and mixed forests (MF). Individual sites are shown as jittered points, with the color indicating the AE‐TE coupling and the shape indicating the network. Medians for each PFT × AE/TE × network combination are shown as larger points. Boxplots indicate the PFT/network overall EBC distribution.
**Figure S11b:** Distribution of EBC (RMA slopes at half‐hourly time scale) for each non‐forest ecosystem plant functional type (PFT), separated by network. The number of sites per PFT and network is indicated below each group (n). PFTs are: croplands (CRO), grasslands (GRA), savannas/shrublands (SHR|SAV), and wetlands (WET). Individual sites are shown as jittered points, with the color indicating the AE‐TE coupling and the shape indicating the network. Medians for each PFT × AE/TE × network combination are shown as larger points. Boxplots indicate the PFT/network overall EBC distribution.

## Data Availability

Raw eddy‐covariance data used as input are publicly available from the ICOS Carbon Portal (ICOS RI et al. 2025; https://doi.org/10.18160/S6HM‐CP8Q) and the NEON data portal (NEON (47) 2025; https://doi.org/10.48443/r7zp‐y487). The processed datasets and R code supporting the findings of this study are openly available at https://doi.org/10.5281/zenodo.19608436 and https://doi.org/10.5281/zenodo.19631582, respectively.
