# Supplementary material for: Bridging the Energy Balance Gap in Eddy‐Covariance Measurements: Insights From Standardized Network Data
Source: Glob Chang Biol. 2026 May 4;32:e70892. doi: 10.1111/gcb.70892 (PMC13137408; doi:10.1111/gcb.70892)
Supplement: Supplementary file 1 — Figure S1: Map of the 84 stations included in the analysis, covering a wide range of terrestrial ecosystems (IGBP, named as plant functional types (PFT) in the main text). Thirty‐eight stations belong to the ICOS network and 46 stations to the NEON network. Table S1: Vegetation cover (%) and leaf area index (LAI) stats at the analyzed stations. ICOS stations, assessed for 2024: vegetation cover was calculated using Sentinel‐2 Enhanced Vegetation Index (EVI; range: −1 to +1, with active vegetation typically > 0.20), pixels with EVI ≥ 0.25 were classified as vegetated; LAI is measured indirectly at forest sites with digital hemispherical photography (DHP) or a linear ceptometer, depending on the seasonal max value, at nonforest sites, LAI is measured with a ceptometer. NEON stations: vegetation covers were not available; LAI values were derived from MODIS imagery: LAI values were extracted for a 1 km radius surrounding each tower for 2022–2023 then the average was taken across the pixels to generate site‑level LAI, and then seasonal means for winter and summer were computed. Figure S2: Vegetation cover maps at analyzed ICOS stations. Green and grey pixels indicate vegetated and nonvegetated areas, respectively. The EC flux footprint climatology (80% cumulative contribution, red contours) defines the reference surface for the analysis. Vegetation cover was assessed for 2024 using Sentinel‐2 Enhanced Vegetation Index (EVI; range: −1 to +1, with active vegetation typically > 0.20). Pixels with EVI ≥ 0.25 were classified as vegetated, and annual medians computed accordingly. Red numbers in the bottom‐right corner indicate the fractional vegetation cover (%) within the EC flux footprint. Low vegetation cover values at SE‐Nor (ENF) are due to a clear‐cutting event in 2022–2023. Table S2: Species composition at crop stations. Figure S3: Distribution of energy balance closure (EBC) at half‐hour resolution as estimated by OLS (ordinary linear) and RMA (reduced major axis) slop [file GCB-32-e70892-s001.pdf]

Bridging the energy balance gap in Eddy-Covariance measurements: insights from standardized network data.

Giacomo Nicolini, David Durden, Luca Di Fiore, Christopher Florian, Simone Sabbatini, Bert Gielen, Arne Iserbyt, Benjamin Loubet, Ivan Mammarella, Adriana Mariotti, Maarten Op de Beeck, Caleb Slemmons, Carlo Trotta, Adam Young, Abad Chabbi, Iris Feigenwinter, Bernard Heinesch, Natalia Kowalska, Matthias Mauder, Ladislav Šigut, Michiel van der Molen, Flavio Bastos Campos, Daniel Berveiller, Christian Brümmer, Matthias Cuntz, Jean-Christophe Domec, Benjamin Dumont, Silvano Fares, Damiano Gianelle, Rasmus Jensen, Carmen Kalalian, Natascha Kljun, Holger Lange, Jean-Marc Limousin, Erik Lundin, Antonio Manco, Leonardo Montagnani, Eiko Nemitz, Matthias Peichl, Erkka Rinne, Marilyn Roland, Marius Schmidt, Guillaume Simioni, Abin Thomas, Caroline Vincke, Dario Papale.

SUPPLEMENTARY MATERIAL

Stations map

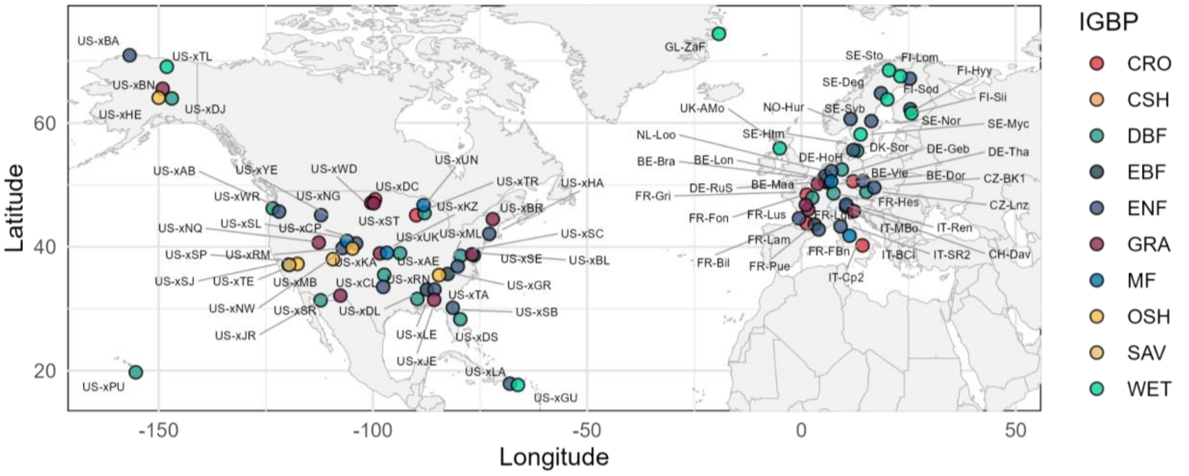

FIGURE S1 | Map of the 84 stations included in the analysis, covering a wide range of terrestrial ecosystems (IGBP, named as plant functional types (PFT) in the main text). 38 stations belong to the ICOS network and 46 stations to the NEON network.

Vegetation cover

TABLE S1 | Vegetation cover (%) and leaf area index (LAI) stats at the analyzed stations. ICOS stations, assessed for 2024: vegetation cover was calculated using Sentinel-2 Enhanced Vegetation Index (EVI; range: -1 to +1, with active vegetation typically > 0.20), pixels with EVI ≥ 0.25 were classified as vegetated; LAI is measured indirectly at forest sites with digital hemispherical photography (DHP) or a linear ceptometer, depending on the seasonal max value, at non-forest sites, LAI is measured with a ceptometer. NEON stations: vegetation covers were not available; LAI values were derived from MODIS imagery: LAI values were extracted for a 1km radius surrounding each tower for 2022-2023 then the average was taken across the pixels to generate site-level LAI, and then seasonal means for winter and summer were computed.

station	vegetation cover		LAI	
	summer	winter	summer	winter
BE-Bra	100.0	75.0	3.0	1.9
BE-Dor	100.0	100.0	0.8	0.8
BE-Lon	3.0	67.0	3.4	1.5
BE-Maa	100.0	75.0	1.4	0.8
BE-Vie	99.0	59.0	3.5	2.5
CH-Dav	98.0	67.0	3.4	
CZ-BK1	100.0	6.0	4.1	3.6
CZ-Lnz	99.0	0.0	6.0	1.5
DE-Geb	14.0	99.0	3.1	
DE-HoH	100.0	9.0	4.2	1.7
DE-RuS	100.0	1.0	3.6	1.5
DE-Tha	99.0	66.0	4.0	2.5
DK-Sor	100.0	92.0	5.3	1.6
FI-Hyy	100.0	99.0	1.8	1.6
FI-Sii	100.0	0.0	0.5	
FR-Bil	100.0	100.0	1.9	1.6
FR-FBn	100.0	97.0	2.6	2.5
FR-Fon	100.0	16.0	4.9	1.9
FR-Gri	100.0	3.0	4.3	2.8
FR-Hes	100.0	4.0	5.9	2.0
FR-Lqu	100.0	100.0	0.8	0.4
FR-Pue	100.0	100.0	2.5	2.6
GL-ZaF	6.0	0.0	0.7	
IT-BCi	100.0	100.0	3.9	
IT-Cp2	92.0	84.0	4.3	4.0
IT-MBo	100.0	0.0	1.5	
IT-Ren	100.0	45.0	4.1	3.9
IT-SR2	100.0	97.0	3.1	2.9
NL-Loo	100.0	92.0	2.7	2.2
NO-Hur	98.0	72.0	2.2	2.3
SE-Deg	99.0	0.0	0.6	
SE-Htm	100.0	93.0	4.4	4.3
SE-Nor*	32.0	1.0	2.7	2.5
SE-Sto	100.0	0.0	0.8	
SE-Svb	96.0	89.0	3.5	
UK-AMo	100.0	99.0	1.3	
US-xAB			4.6	2.5
US-xBA			0.7	0.1

station	vegetation cover		LAI	
	summer	winter	summer	winter
US-xBR			4.9	0.6
US-xBL			2.5	0.6
US-xBN			3.2	0.2
US-xCL			1.6	0.4
US-xCP			0.7	0.1
US-xDC			2.6	0.0
US-xDJ			1.4	0.1
US-xDL			4.4	0.6
US-xDS			2.2	1.1
US-xGR			4.7	0.7
PR-xGU			3.7	3.6
US-xHA			5.4	0.7
US-xHE			2.0	0.1
US-xJE			3.1	1.1
US-xJR			0.2	0.2
US-xKA			2.6	0.3
US-xKZ			3.1	0.3
PR-xLA			1.1	1.1
US-xLE			4.9	0.8
US-xML			4.6	0.6
US-xMB			0.3	0.1
US-xNW			0.9	0.1
US-xNG			1.8	0.1
US-xAE			1.0	0.3
US-xNQ			0.3	0.2
US-xRN			4.4	0.5
US-xSB			1.9	1.1
US-xPU			2.6	2.0
US-xRM			1.4	0.6
US-xSC			5.3	0.5
US-xSE			4.8	0.6
US-xSJ			0.8	1.0
US-xSP			1.8	0.9
US-xSR			0.3	0.2
US-xST			5.0	0.3
US-xSL			0.9	0.1
US-xTA			5.6	2.0
US-xTE			1.5	0.7
US-xTL			1.4	0.1
US-xTR			5.0	0.4
US-xUK			2.9	0.3
US-xUN			5.1	0.2
US-xWD			2.0	0.0
US-xWR			3.8	1.4
US-xYE			1.2	0.0

* The low vegetation cover and LAI values reported for SE-Nor (classified as evergreen needleleaf forest, ENF) reflect a clear-cutting event that occurred at the site between 2022 and 2023; the reported data refer to 2024 only.

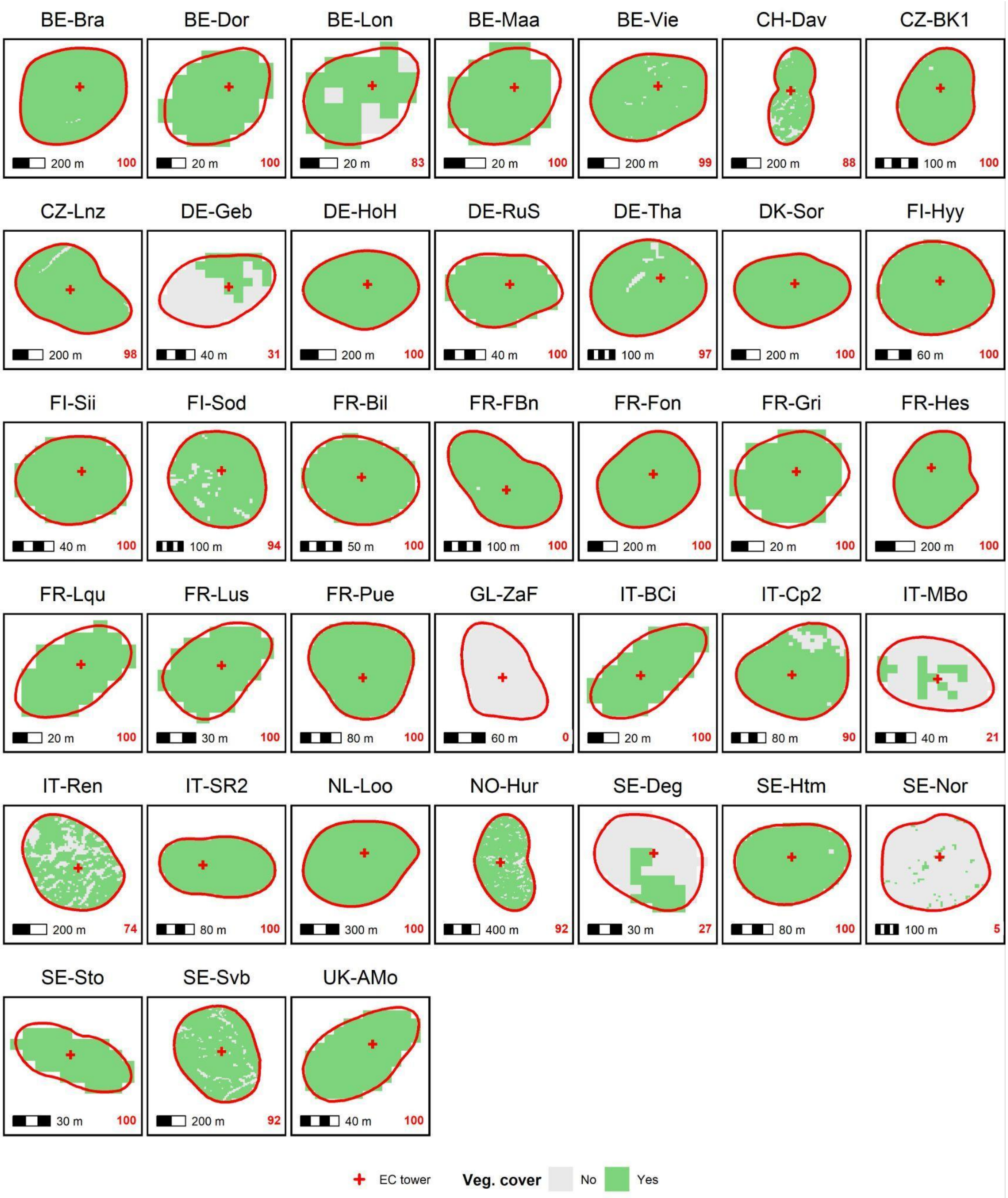

FIGURE S2 | Vegetation cover maps at analyzed ICOS stations. Green and grey pixels indicate vegetated and non-vegetated areas, respectively. The EC flux footprint climatology (80% cumulative contribution, red contours) defines the reference surface for the analysis. Vegetation cover was assessed for 2024 using Sentinel-2 Enhanced Vegetation Index (EVI; range: -1 to +1, with active vegetation typically > 0.20). Pixels with EVI ≥ 0.25 were classified as vegetated, and annual medians computed accordingly. Red numbers in the bottom-right corner indicate the fractional vegetation cover (%) within the EC flux footprint. Low vegetation cover values at SE-Nor (ENF) are due to a clear-cutting event in 2022–2023.

Species composition at crop stations

TABLE S2 | Species composition at crop stations.

station	species	sowing date
BE-Lon	Triticum aestivum L.	20161029
	Sinapis alba L.	20170907
	Solanum tuberosum L.	20180423
	Triticum aestivum L.	20181010
	Avena sativa L.	20190809
	Vicia faba L.	20190809
	Beta vulgaris L.	20200401
	Spinacia oleracea L.	20210401
	Phaseolus vulgaris L.	20210723
	Triticum aestivum L.	20211028
	Sinapis alba L.	20220913
	Solanum tuberosum L.	20230503
	Triticum aestivum L.	20231016
	Phacelia tanacetifolia Benth.	20240831
	Trifolium alexandrinum L.	20240831
DE-Geb	Raphanus raphanistrum subsp. sativus (L.) Domin	20190902
	Solanum tuberosum L.	20200421
	Triticum aestivum L.	20201013
	Triticum aestivum L.	20210930
	Helianthus annuus L.	20230427
	Triticum aestivum L.	20231001
DE-RuS	Triticum aestivum L.	20171025
	Solanum tuberosum L.	20190426
	Triticum aestivum L.	20191026
	Fagopyrum esculentum Moench	20200826
	Helianthus annuus L.	20200826
	Lupinus angustifolius L.	20200826
	Ornithopus sativus Brot.	20200826
	Phacelia tanacetifolia Benth.	20200826
	Raphanus raphanistrum subsp. sativus (L.) Domin	20200826
	Sinapis alba L.	20200826
	Trifolium alexandrinum L.	20200826
	Beta vulgaris L.	20210421
	Solanum tuberosum L.	20220430
	Triticum aestivum L.	20221015
	Avena sativa L.	20230905
	Calendula officinalis L.	20230905
	Guizotia abyssinica Cass.	20230905
	Helianthus annuus L.	20230905
	Lathyrus oleraceus Lam.	20230905
	Linum usitatissimum L.	20230905
	Lupinus angustifolius L.	20230905
	Phacelia tanacetifolia Benth.	20230905
	Raphanus raphanistrum subsp. sativus (L.) Domin	20230905
	Sorghum bicolor (L.) Moench	20230905
	Trifolium alexandrinum L.	20230905
	Trifolium michelianum Savi	20230905
	Vicia sativa L.	20230905
	Beta vulgaris L.	20240501
	Triticum aestivum L.	20241025

station	species	sowing date
FR-Gri	Triticum aestivum L.	20170925
	Avena sativa L.	20180803
	Zea mays L.	20190420
	Triticum aestivum L.	20191003
	Brassica napus L.	20200818
	Triticum aestivum L.	20211009
	Hordeum vulgare L.	20221013
	Beta vulgaris L.	20240413
	Triticum aestivum L.	20241202
IT-BCi	Medicago sativa L.	20191014
	Zea mays L.	20230531
	Lolium perenne L.	20231013
	Zea mays L.	20240607
US-xSL	Triticum aestivum L.	
	Zea mays L.	
	Panicum miliaceum L.	
	Sorghum bicolor (L.) Moench	
	Pennisetum glaucum (L.) R.Br.	
	Helianthus annuus L.	

Soil heat flux storage equation

The soil heat flux storage S_G was estimated using soil temperature profile measurement (Eq. 2 in the main text) as:

$$S_G = \sum_{i=1}^{i=n} \left(c_{s,i} \frac{\Delta T_{s,i}}{\Delta t} \Delta z_i \right)$$

where $c_{s,i}$ (J °C^{-1} m^{-3}) is the soil volumetric heat capacity calculated as a function of soil moisture at the layer i, $\Delta T_{S,i}$ (°C) is the temporal temperature change ($T_{S,t+\Delta t} - T_{S,t}$) measured at layer i, Δz_i (0.05 m) is the thickness of layer i, Δt is the averaging interval (1800 s), and the summation is performed over all layers between the soil surface and the depth at which G is measured. Volumetric heat capacity of soil (c_s, J °C^{-1} m^{-3}) can be calculated as:

$$c_s = \varphi_{sm}\rho_{sm}\theta_{sm} + \varphi_o\rho_o\theta_o + \varphi_w\rho_w SWC/100$$

Where φ is the specific heat per unit mass of soil minerals (*sm*), organic matter (*o*) and water (*w*), ρ is the density of particles and θ is the volume fraction, *SWC* is the soil water content at 0.05 m (%).
For all sites, except mires, we neglect the contribution of organic matter to c_s and assume an average bulk density (*BD*) of 2.65 g cm^{-3}. The working equation for c_s then becomes:

$$c_s = \varphi_{sm}BD + \varphi_w\rho_w SWC/100$$

For wetland sites, we only consider organic material (Sphagnum peat) and water contribution to c_s. We considered a value of 0.7 J g^{-1} °C for the specific heat of Sphagnum peat (φ_{peat}) and we take a fixed dry peat bulk density (BD_{peat}) of 0.05 g cm^{-3} (this value agrees with field measurements done at SE-Deg and FI-Sii). The equation for c_s of wetland is then:

$$c_s = \varphi_{peat}BD_{peat} + \varphi_w\rho_w SWC/100$$

OLS and RMA regression comparison

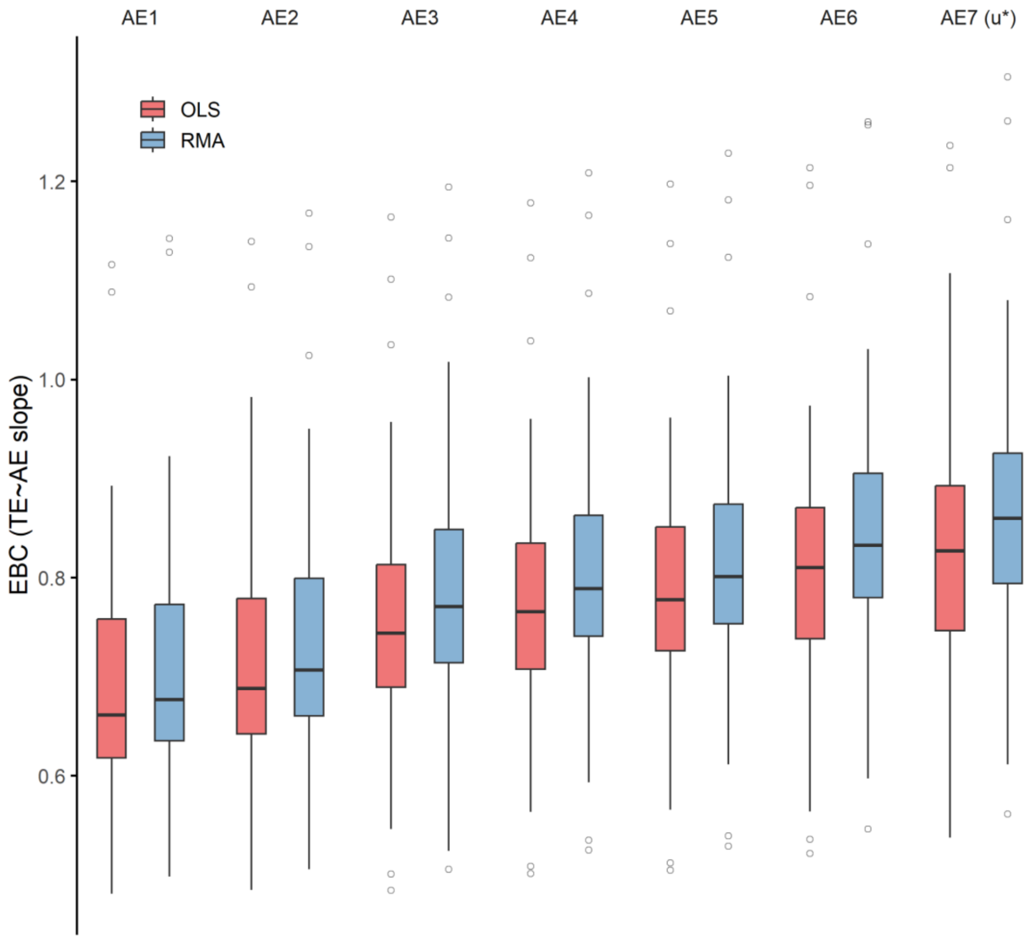

FIGURE S3 | Distribution of energy balance closure (EBC) at half-hour resolution as estimated by OLS (ordinary linear) and RMA (reduced major axis) slopes for each AE/TE pairing (same as Fig.1 and Fig. 2 of the main text: AE1 = NETRAD; AE2 = NETRAD − G; AE3 = NETRAD − G − S_G; AE4 = NETRAD − G − S_G − S_H − S_{LE}; AE5 = AE4 − S_{pho}; AE6 = AE5 − S_{bio}; AE6(u*) = AE6 with *u**-filtered TE). Boxplots show the median and interquartile range, while colors distinguish the regression method.

Sensitivity of energy balance closure to spatial variability in soil heat flux

The representativeness of soil heat flux (SHF) measurements is often considered a potential source of uncertainty in energy balance studies, as heat flux plates sample only a very small soil volume and may not fully capture spatial variability within the turbulent flux footprint (Kustas et al., 2000; Shao et al., 2008). This issue can be particularly relevant in ecosystems characterized by heterogeneous vegetation cover, variable soil properties, or patchy radiation environments.

To assess the potential impact of this limitation on our results, we conducted a sensitivity analysis at the ICOS sites, equipped with 3 to 5 SHF measurement points and placed systematically around the tower to maximize the representativeness of spatial variability. Measurement points are indeed located within permanent sample plots, and the position of these plots is determined during the site labelling phase and immediately checked to verify that soil and vegetation cover variability within the target area is adequately represented. SHF

is measured by self-calibrating plates that periodically apply a known heat pulse to estimate an in-situ calibration factor, reducing biases associated with plate thermal conductivity and soil contact resistance.
For each site, we recomputed energy balance closure using different combinations of sensors to estimate G. Combinations were set based on permutations of the sensors used, leaving one out. Only combinations including three or four sensors were considered, since estimates based on fewer sensors would not provide a meaningful representation of spatial variability. The resulting changes in EBC (RMA slopes) relative to the reference configuration used in the main manuscript (i.e., H+LE ~ AE3, where AE3 = NETRAD − G − S_G and G − S_G is the storage corrected soil heat flux taken as average of all the available sensors) are shown in figure S4.
The results show that the choice of sensor combinations produces only minor variations in the estimated EBC slope relative to the reference configuration. Across sites, most deviations remain within ±0.01–0.02, with no consistent tendency toward either improved or reduced closure. This finding is independent of the fractional vegetation cover (see the Vegetation cover Section for more details).
Interestingly, the larger variability is observed at a few sites, FR-Gri, IT-BCi, DE-Geb and BE-Lon, which are all crops. However, the overall magnitude of the effect remains small.

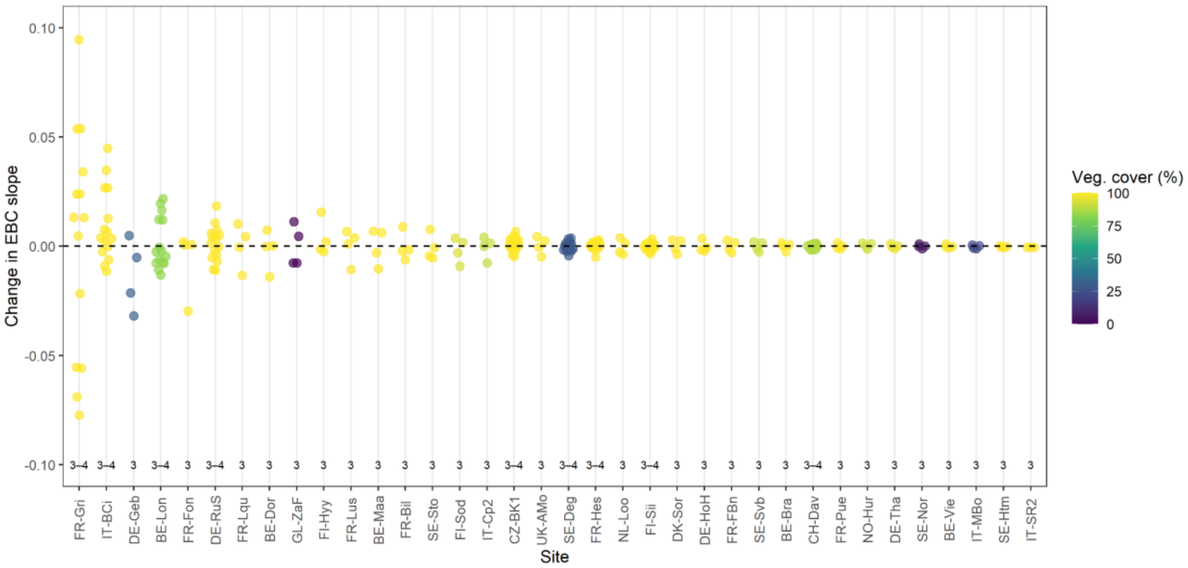

FIGURE S4 |Sensitivity of energy balance closure (EBC) to spatial sampling of storage corrected soil heat flux (G+S_G). The plot shows the change in EBC RMA slopes (H+LE ~ AE) relative to the reference configuration (AE3 = NETRAD − G − S_G, used in the main analysis) when different combinations of soil heat flux sensors are used to estimate G. Only combinations including three or four sensors were considered. Each point represents a different sensor combination at a given site. Point colors indicate fractional vegetation cover derived from remote sensing. Numbers on the bottom side represent the used combinations: 3 means all the permutations with 3 sensors (for stations with 4 measurement points), 3-4 means all the permutations with 3 and 4 sensors (for stations with 5 measurement points).

These findings suggest that, for the sites analyzed here, spatial variability in SHF does not have a significant impact on the estimated energy balance closure.
Although point measurements of G cannot fully resolve spatial heterogeneity across the turbulent flux footprint, the resulting uncertainty appears to be small relative to other sources of imbalance considered in this study.

Friction velocity filtering, gap-filling and partitioning procedure

All flux processing steps followed the standardized ONEFlux framework.

u* threshold determination

Low-turbulence filtering was applied following the standardized procedure described in Papale et al. (2006). Briefly, nighttime net ecosystem exchange (NEE) data were stratified by season, temperature classes and friction velocity (*u**) bins. Within each temperature class, NEE was examined as a function of *u** to identify the threshold above which fluxes become independent of turbulence intensity, indicating sufficient mixing. The *u** threshold was determined using a moving point test that detects stabilization of mean flux values across increasing *u** classes. To account for uncertainty, a bootstrap approach was implemented by repeatedly resampling the dataset and recalculating the threshold, generating a distribution of *u** values. The median threshold was used for filtering, and associated variability was propagated as part of flux uncertainty.

Gap-filling procedure (MDS method)

Missing flux data were filled using the Marginal Distribution Sampling (MDS) algorithm described in Reichstein et al. (2005). The method exploits short-term meteorological similarity by replacing missing values with measured fluxes observed under comparable conditions of incoming radiation, air temperature, and vapor pressure deficit within a moving temporal window. When insufficient matches were found within narrow environmental tolerances, the search window and similarity criteria were progressively relaxed. If no suitable meteorological analogues were available, diurnal mean courses were used as a fallback. This hierarchical approach preserves observed flux-environment relationships while minimizing model structural assumptions.

Daytime flux partitioning

Daytime partitioning of NEE into gross primary production (GPP) and ecosystem respiration (Reco) was performed following the method of Lasslop et al. (2009). The approach fits a light-response curve to daytime NEE data using a rectangular hyperbolic function that describes photosynthetic uptake as a function of incoming shortwave radiation, combined with a temperature-dependent formulation of ecosystem respiration.
Specifically, NEE is modelled as the sum of a light-saturated gross assimilation term and an exponential temperature-driven respiration component. Parameters are estimated within moving time windows to capture seasonal variability in physiological responses. The respiration temperature sensitivity is constrained using nighttime data, while daytime data are used to optimize light-response parameters (initial slope, maximum uptake, and curvature). The fitted model is then applied to derive continuous estimates of GPP and Reco under observed environmental conditions.

Friction velocity filtering assessment

Physical basis for friction velocity filtering

Friction velocity (*u**) filtering is widely applied in eddy covariance measurements to remove periods when turbulent mixing is insufficient to ensure representative flux measurements. The primary target of *u** filtering is nighttime conditions under stable atmospheric stratification, when radiative surface cooling suppresses vertical turbulent exchange. Under these

conditions, horizontal advective transport can become significant relative to vertical turbulent fluxes, violating the fundamental assumptions of the eddy covariance method.

The relationship between low *u** conditions and advective fluxes warrants clarification. Strong advective events driven by synoptic-scale pressure gradients typically occur with elevated wind speeds and substantial *u** values. However, the low-turbulence regime targeted by *u** filtering represents a distinct physical process: thermally-driven drainage flows and mesoscale circulations that can transport energy horizontally even under relatively light winds when vertical mixing is sufficiently suppressed by stable stratification. The critical factor is the ratio of horizontal to vertical transport. When this ratio becomes unfavorable, as occurs under strong surface-based inversions, measured vertical fluxes no longer represent true surface-atmosphere exchange, regardless of whether horizontal winds are weak or moderate.

Additional conditions triggering *u** filtering include *i*) decoupling events: under highly convective daytime conditions with light winds, the surface layer can decouple from the mixed layer aloft, creating representativeness issues even when *u** remains moderate. Filtering is most common during transition periods (morning/evening) when convection weakens but stable stratification has not yet fully developed; *ii*) site-specific threshold variability: *u** thresholds are determined site-specifically following the moving point test method (Papale et al., 2006) and vary substantially with canopy height, surface roughness, and topographic setting. Forest sites typically have higher thresholds (0.3–0.5 m s^{-1}), while smooth cropland or grassland sites exhibit lower values (0.1–0.2 m s^{-1}). Consequently, the meteorological conditions modulating filtering differ systematically across sites.

Sites located on slopes, in valley bottoms, or near complex terrain experience more frequent drainage flows and thermally-driven circulations under stable conditions, regardless of vegetation cover.

Local meteorological climatology such as wind patterns, frequency of calm nights, cloud cover distribution, and synoptic weather regimes determine turbulence characteristics more strongly than canopy structure alone. Also, measurement configuration impact on the filtering as sensor height above the canopy influences *u** thresholds and the frequency of low-turbulence conditions, even within the same ecosystem type. Lastly, canopy architecture within a single ecosystem type exerts a within-site influence on data filtering because substantial variation in canopy height, density, and roughness length creates site-specific turbulence regimes.

Site-specific patterns of data removal

We quantified the percentage of half-hourly data removed by *u** filtering across all analyzed sites and examined whether ecosystem type systematically influences data loss (Tab. S3). *u** filtering removed between 7.5% and 62.5% of half-hourly observations across sites, with an overall median removal rate of 33.6%. This wide range reflects substantial site-to-site variability in turbulence climatology and local environmental conditions.

Contrary to expectations, ecosystem type did not systematically predict the amount of data removed by *u** filtering (Tab. S3, Fig. S5). While median removal rates varied from 21.7% (wetlands, WET) to 47.5% (open shrublands, OSH), the ranges overlapped extensively across vegetation types. For instance:

Wetlands (WET): 7.5–35.7% (median 21.7%)
Grasslands (GRA): 11.2–45.5% (median 31.1%)
Croplands (CRO): 32.4–51.2% (median 39.8%)
Evergreen needleleaf forests (ENF): 13.4–56.9% (median 37.3%)
Deciduous broadleaf forests (DBF): 16.7–50.8% (median 28.6%)

Some ecosystem types exhibited narrow ranges (closed shrublands, CSH: 25.3%; savannas, SAV: 45.7%), but these categories contained only one or two sites, precluding robust statistical inference. Ecosystem types with larger sample sizes (GRA, CRO, ENF, DBF) all showed

30%–40% in data removal, indicating that within-ecosystem variability exceeded between-ecosystem differences.

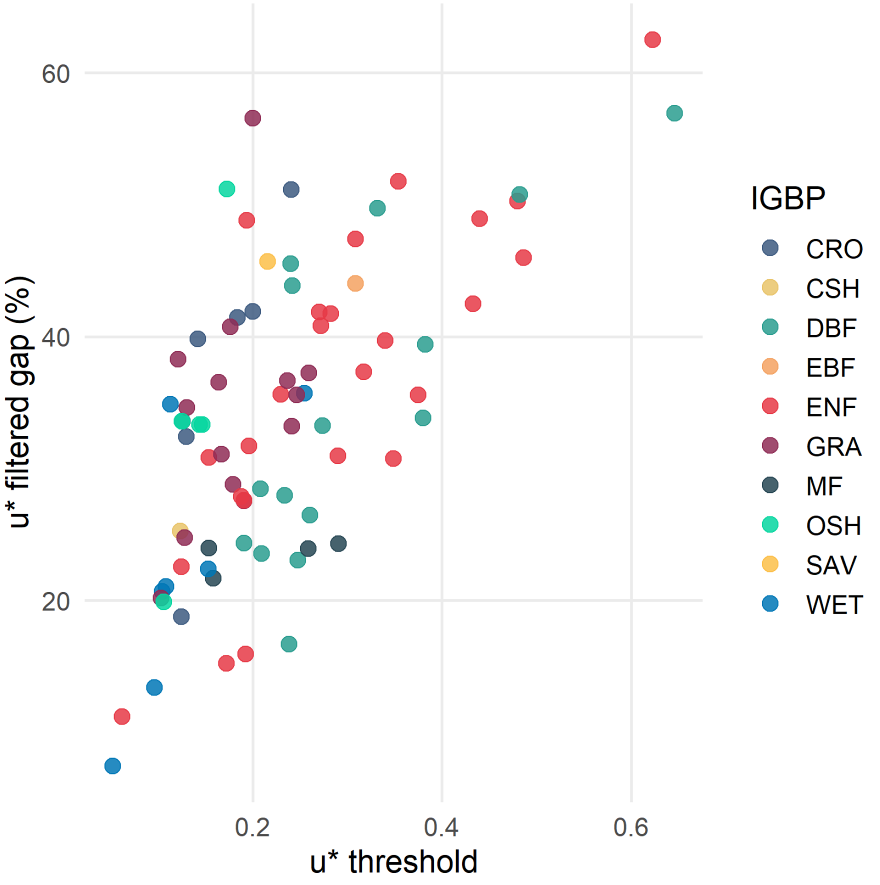

FIGURE S5 | Relationship between site-specific *u** threshold values and percentage of data removed by filtering. Points are colored by ecosystem type.

This result indicates that data loss from *u** filtering is driven primarily by site-specific factors rather than vegetation type. Sites with higher site-specific *u** thresholds generally lost more data to filtering (Fig. S5), as expected. However, this relationship was highly variable, with substantial scatter indicating that threshold magnitude alone weakly explains variance in data removal.

TABLE S3 | Friction velocity (*u**) filtering assessment. *PFT* is the plant functional types, *u* thrs* is the site-specific calculated *u** threshold for data filtering, *u* filter 24H*, *u* filter NT*, and *u* filter DT* are the amount of data discarded (%) because of the *u** filtering for the whole period, nighttime and daytime data respectively, EBC_RMA is the energy balance closure computed at half-hourly scale (RMA slope, %), EBC_BR is the energy balance closure computed at monthly scale (bulk ratio, %).

site	PFT	u* thrs	u* filter 24H	u* filter NT	u* filter DT	EBC_RMA	EBC_BR
BE-Bra	ENF	0.19	15.9	25.6	6.2	1.04	0.86
BE-Dor	GRA	0.16	36.6	51.0	22.0	1.06	0.82
BE-Lon	CRO	0.20	41.9	55.4	28.4	1.19	0.77
BE-Maa	CSH	0.12	25.3	40.9	9.6	0.97	0.68
BE-Vie	MF	0.29	24.3	36.2	12.3	0.99	0.77
CH-Dav	ENF	0.27	41.8	53.4	30.2	0.57	0.45
CZ-BK1	ENF	0.44	48.9	62.6	35.2	1.25	1.10
CZ-Lnz	DBF	0.19	24.4	37.0	11.7	0.89	0.80

site	PFT	u* thrs	u* filter 24H	u* filter NT	u* filter DT	EBC_RMA	EBC_BR
DE-Geb	CRO	0.14	39.8	56.2	23.4	1.29	1.24
DE-HoH	DBF	0.38	33.8	46.4	21.2	1.25	0.87
DE-RuS	CRO	0.13	32.4	48.2	16.4	0.88	0.53
DE-Tha	ENF	0.35	30.7	45.4	16.0	0.92	0.82
DK-Sor	DBF	0.24	16.7	26.3	7.0	0.95	0.93
FI-Hyy	ENF	0.37	35.6	48.8	22.3	0.93	0.71
FI-Sii	WET	0.10	20.7	32.6	8.7	0.89	0.67
FI-Sod	ENF	0.34	39.7	52.9	26.5	1.16	0.64
FR-Bil	ENF	0.20	31.7	49.2	14.1	1.17	0.80
FR-FBn	ENF	0.49	46.0	58.4	33.4	0.95	0.77
FR-Fon	DBF	0.25	23.1	34.8	11.2	0.85	0.68
FR-Gri	CRO	0.24	51.2	65.6	36.6	1.05	0.60
FR-Hes	DBF	0.23	27.9	40.4	15.5	0.89	0.63
FR-Lqu	GRA	0.19	27.5	36.1	19.1	0.84	0.70
FR-Lus	GRA	0.13	24.8	36.9	12.6	1.04	0.88
FR-Pue	EBF	0.31	44.0	57.3	30.8	0.95	0.73
GL-ZaF	WET	0.05	7.5	9.3	5.7	0.67	0.69
IT-BCi	CRO	0.18	41.4	61.0	21.8	1.00	0.92
IT-Cp2	MF	0.16	21.7	39.6	3.6	1.45	1.84
IT-MBo	GRA	0.12	38.3	53.2	23.3	0.89	1.08
IT-Ren	ENF	0.31	47.4	61.6	33.1	0.72	1.38
IT-SR2	ENF	0.28	41.7	60.2	23.2	1.15	0.79
NL-Loo	ENF	0.17	15.2	25.3	5.1	0.98	0.63
NO-Hur	ENF	0.23	35.6	46.8	24.4	0.66	0.24
SE-Deg	WET	0.11	21.1	28.9	13.3	0.80	0.55
SE-Htm	ENF	0.43	42.5	57.2	27.8	0.95	0.52
SE-Nor	ENF	0.32	37.3	48.9	25.8	0.95	0.72
SE-Sto	WET	0.15	22.4	28.0	16.8	0.80	0.45
SE-Svb	ENF	0.48	50.2	61.6	38.9	0.98	0.73
UK-AMo	WET	0.25	35.7	46.6	24.7	1.16	0.77
US-xAB	ENF	0.19	48.8	70.8	26.9	1.15	0.77
US-xAE	GRA	0.18	28.8	45.9	11.6	0.79	0.89
US-xBA	WET	0.10	13.4	16.3	10.5	0.65	0.91
US-xBL	DBF	0.13	33.6	55.3	12.0	0.84	0.72
US-xBN	ENF	0.06	11.2	15.2	7.2	1.60	3.60
US-xBR	DBF	0.24	45.5	61.8	29.5	0.92	0.79
US-xCL	GRA	0.26	37.3	58.7	15.8	0.75	0.84
US-xCP	GRA	0.18	40.7	58.9	22.4	0.87	0.90
US-xDC	GRA	0.25	35.6	46.7	24.3	0.81	0.73
US-xDJ	ENF	0.15	30.8	43.5	18.2	0.88	0.78
US-xDL	MF	0.15	24.0	40.7	7.2	0.71	0.81
US-xDS	GRA	0.13	34.6	58.0	11.6	0.71	0.79
US-xGR	DBF	0.24	43.8	54.1	33.6	0.95	0.64
US-xHA	DBF	0.38	39.4	54.8	24.1	0.77	0.68
US-xHE	OSH	0.17	51.2	59.8	42.6	0.83	0.48
US-xJE	ENF	0.19	27.9	45.1	10.8	0.79	0.87
US-xJR	OSH	0.13	33.6	55.1	11.4	0.91	0.91
US-xKA	GRA	0.10	20.2	33.0	7.4	0.77	0.74
US-xKZ	GRA	0.24	33.2	44.6	21.6	0.70	0.73
US-xLA	GRA	0.20	56.5	88.7	25.2	0.70	0.95
US-xMB	OSH	0.14	33.3	50.3	16.0	0.80	0.85
US-xML	DBF	0.65	56.9	63.2	50.7	0.86	0.82
US-xNG	GRA	0.17	31.1	45.7	16.1	0.79	0.91

site	PFT	u* thrs	u* filter 24H	u* filter NT	u* filter DT	EBC_RMA	EBC_BR
US-xNQ	OSH	0.11	19.9	30.7	8.9	0.86	0.81
US-xNW	ENF	0.62	62.5	62.8	62.3	0.88	0.62
US-xRM	ENF	0.29	30.9	47.9	14.2	0.78	0.85
US-xRN	DBF	0.33	49.7	63.7	35.6	0.91	1.00
US-xSB	ENF	0.19	27.6	45.6	9.5	0.85	0.83
US-xSC	DBF	0.48	50.8	61.0	40.6	0.83	1.00
US-xSE	DBF	0.21	28.4	45.4	11.7	0.73	0.83
US-xSJ	SAV	0.22	45.7	69.4	21.9	0.68	0.77
US-xSL	CRO	0.12	18.8	30.0	7.7	0.79	0.74
US-xSR	OSH	0.15	33.3	53.6	13.0	0.88	0.91
US-xST	DBF	0.27	33.2	51.1	15.5	0.83	0.81
US-xTA	ENF	0.35	51.8	68.7	34.7	0.74	0.75
US-xTL	WET	0.11	34.9	39.8	30.0	0.75	0.65
US-xTR	DBF	0.21	23.5	39.0	8.1	0.83	0.82
US-xUK	DBF	0.26	26.5	41.4	11.7	0.74	0.82
US-xUN	MF	0.26	23.9	37.9	10.0	0.85	0.82
US-xWD	GRA	0.24	36.7	50.8	22.3	0.81	0.72
US-xWR	ENF	0.12	22.6	33.5	11.9	1.02	0.72
US-xYE	ENF	0.27	40.8	57.9	23.9	0.90	0.87

IGBP: WET = wetlands, MF = mixed forests, CSH = closed shrublands, DBF = deciduous broadleaf forests, GRA = grasslands, ENF = evergreen needleleaf forests, CRO = croplands, EBF = evergreen broadleaf forests, SAV = savannas, OSH = open shrublands.

Impact on energy balance closure

A critical question is whether the percentage of data removed by *u** filtering systematically affects energy balance closure quality. If filtering removes primarily noise or low-quality data, higher removal rates should not degrade, and may even improve, EBC. Conversely, if filtering removes good quality flux data, higher removal rates might reduce closure and biasing temporal integration.

Our analysis reveals no systematic relationship between the percentage of data removed by *u** filtering and energy balance closure quality, both at half-hourly and longer aggregation temporal scales (Fig. S6). Sites losing 10–20% of data to *u** filtering exhibited EBC distributions statistically indistinguishable from sites losing 40–50% (Kruskal-Wallis test, $p = 0.67$).

We interpret this result as evidence that *u** filtering effectively identifies and removes periods when eddy covariance assumptions are violated. Retaining these low-quality periods would introduce systematic biases (typically flux underestimation due to missed advective transport) rather than improve statistical power through increased sample size.

The lack of EBC degradation with increased filtering stringency supports an important methodological principle: the quality of retained flux data is more important than the quantity removed. This finding justifies the widespread application of site-specific *u** thresholds in network flux processing, even when substantial data loss occurs, and argues against relaxing thresholds to maximize data coverage at the expense of data quality. These results reinforce the importance of rigorous, site-specific quality control in eddy covariance flux measurements and demonstrate that data coverage alone is not a reliable indicator of flux dataset quality.

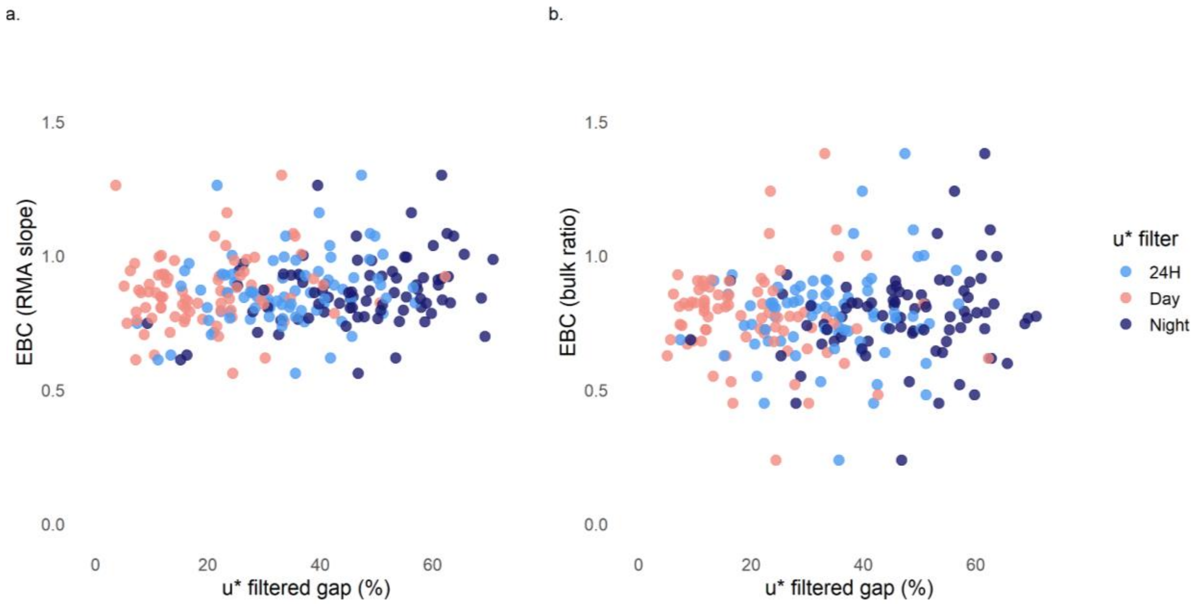

FIGURE S6 | Energy balance closure (EBC) as a function of the percentage of data removed by *u** filtering, by temporal aggregation scale, **a.** 30 minutes (EBC as RMA slope), and **b.** monthly resolution (EBC as bulk ratio).

Sensitivity of energy balance closure to data gaps length and gap-filling

The potential influence of gap filling on energy balance closure (EBC) estimates is an important concern when evaluating closure statistics at aggregated temporal scales. As temporal aggregation increases (e.g. from half-hourly to monthly or seasonal scales), the fraction of gap-filled values contributing to aggregated flux estimates may also increase, potentially affecting the resulting EBC metrics.

To assess this effect, we performed a sensitivity analysis based on the observed fraction of gap-filled data in the dataset. For each site, we quantified the fraction of gap-filled observations relative to the total number of available records. This fraction was calculated considering two cases: a) the overall gap fraction across all variables contributing to the energy balance, including net radiation, soil heat flux, turbulent fluxes, and storage components, and b) the gap fraction affecting only the turbulent fluxes H and LE. Sites were then grouped into three classes of equal size (terciles, n=144) according to these gap fractions.

The resulting distributions of the EBC bulk ratio across temporal aggregation scales are shown in figure S7. When considering the overall gap fraction (Fig. S7 a.), no clear or systematic relationship emerges between the proportion of gap-filled data and the resulting EBC values. Across all temporal scales, the distributions of EBC ratios remain largely overlapping between gap classes, although EBC ratios are shifted toward lower values for higher gap fractions. This indicates that higher fractions of gap-filled observations do not consistently lead to either improved or degraded energy balance closure.

A slightly different behavior emerges when considering only the gap fraction in turbulent fluxes (Fig. S7 b.). In this case, higher gap fractions are associated with a modest tendency toward lower EBC ratios, particularly at longer aggregation scales, although the distributions across classes remain strongly overlapped. This pattern may reflect the central role of turbulent fluxes in determining closure statistics. Gap filling of H and LE typically relies on empirical or semi-empirical models that may smooth short-term variability or extreme events, which can influence the covariance structure between turbulent fluxes and available energy.

Nevertheless, the substantial overlap among gap classes suggests that the amount of gap filling alone does not represent a dominant control over closure statistics within the range of

conditions represented in this dataset. Instead, site characteristics, measurement uncertainties, and the relative contribution of different storage terms likely play a larger role in shaping the observed variability of EBC.

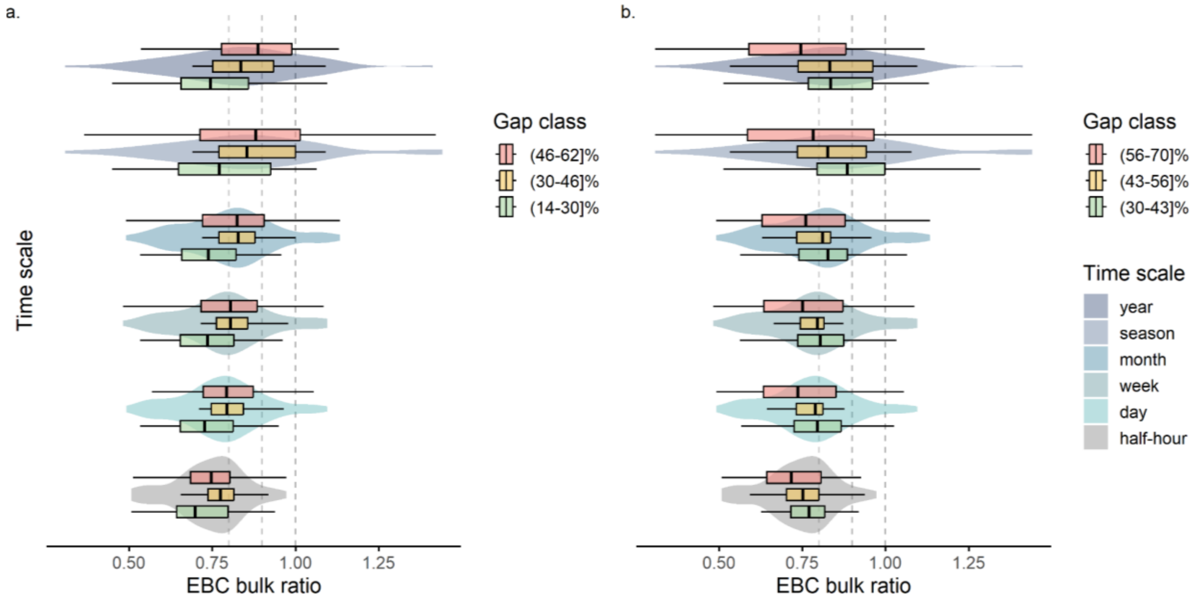

FIGURE S7 | Influence of gap-filled data on energy balance closure (EBC) across temporal aggregation scales. Violin plots show the overall distribution of the EBC bulk ratio for each time scale. Boxplots represent sites grouped into terciles of gap fraction. Panel **a.** shows the overall gap fraction considering all variables entering the energy balance, while panel **b.** shows the gap fraction considering only turbulent fluxes. Gap classes are arranged from lower to higher gap fractions from bottom to top within each violin. Vertical dashed lines indicate reference closure thresholds.

This result can be interpreted in light of the structure of the energy balance equation, through several mechanisms:

- Gap distribution bias: gaps predominantly occur during low-turbulence conditions (nighttime, precipitation, instrument malfunction), when both turbulent fluxes and available energy are reduced. Gap-filling algorithms recreate these low-flux periods, but this does not systematically alter the TE/AE ratio because both numerator and denominator scale proportionally.
- Error compensation: gap-filling can both overestimate and underestimate fluxes depending on atmospheric conditions and algorithm assumptions. Over longer integration periods, these errors may partially compensate rather than accumulate unidirectionally.
- Multi-variable gap complexity: the effective missing data fraction in the energy balance differs from the gap fraction in turbulent fluxes alone, because net radiation, ground heat flux, and storage terms have independent gap patterns that do not necessarily correlate with turbulent flux gaps. Because these variables are affected by different measurement uncertainties and gap patterns, the influence of gap filling in a single component may be partially compensated by variability in other terms of the balance. As a result, the overall fraction of gap-filled data does not translate directly into systematic changes in EBC.
- Quality filtering stringency: sites with high gap fractions may reflect more aggressive quality control filtering, which removes low-quality data that would otherwise degrade closure. Thus, high gap fraction can contradictorily indicate data of higher quality.

Air storage flux and relative humidity effects

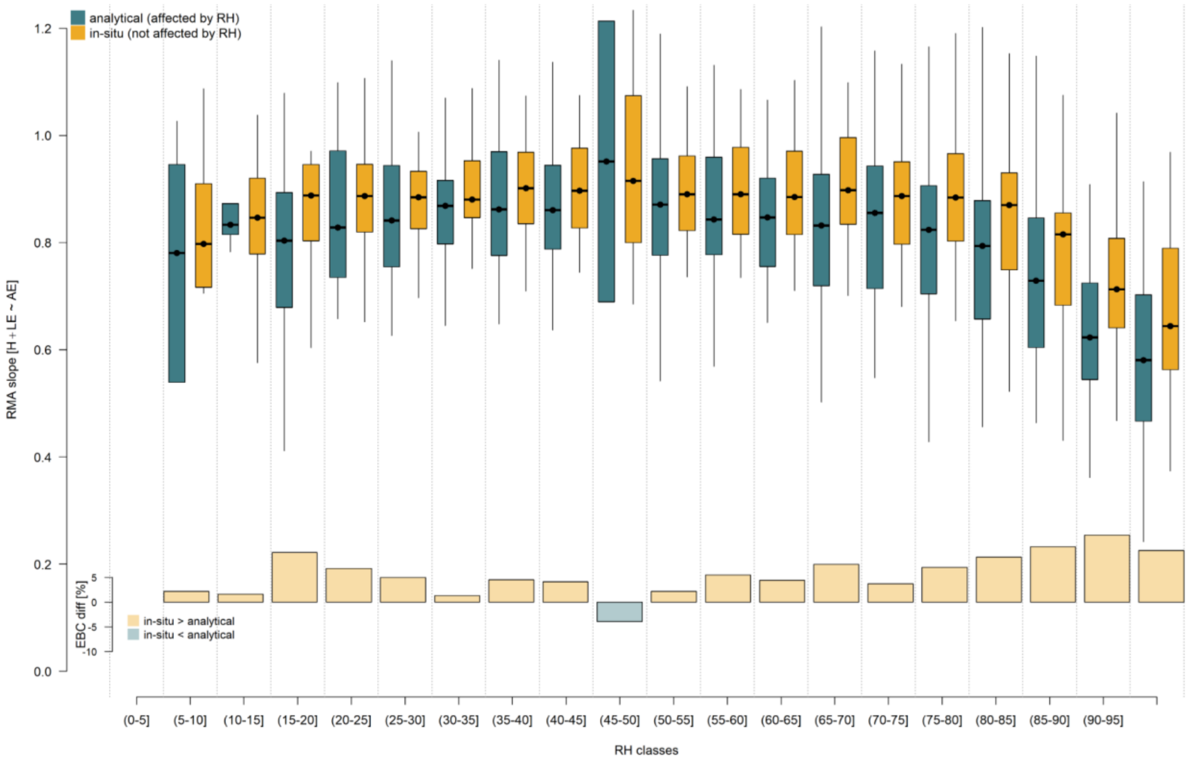

FIGURE S8 | Sensitivity of the energy balance closure (EBC, reported here as RMA slopes at half-hourly time scale) to the effect of air relative humidity (RH) on energy fluxes attenuation.

Site-specific net radiation corrections

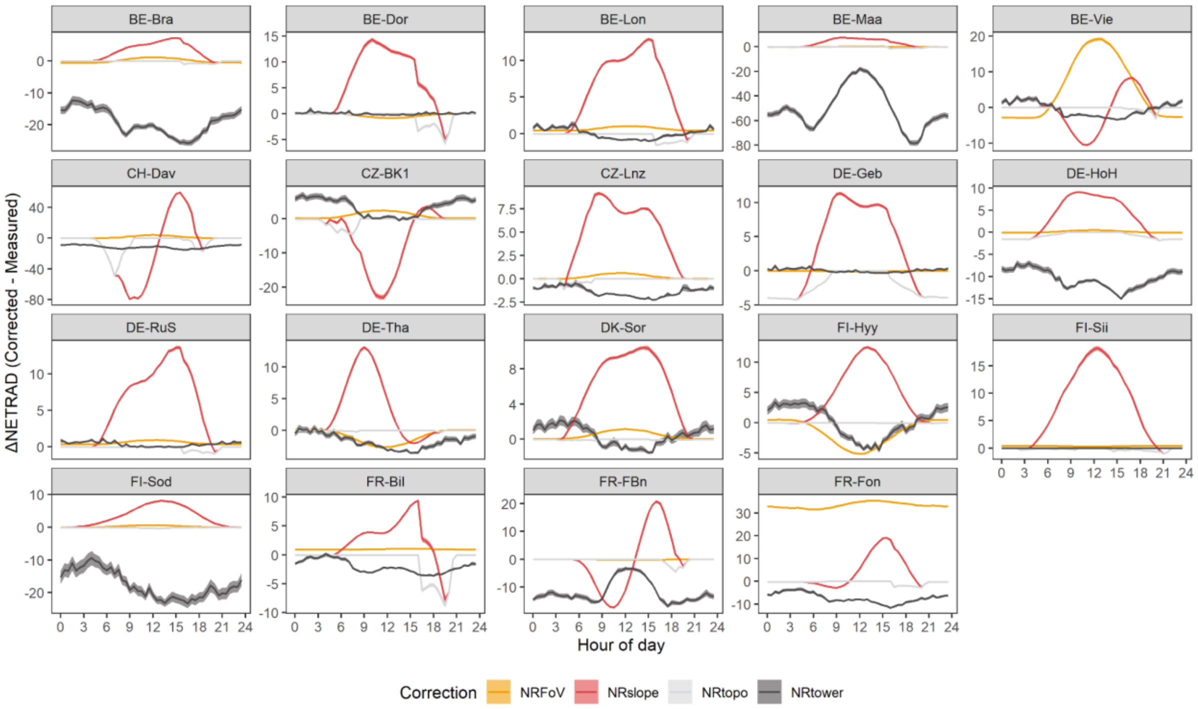

FIGURE S9 a | ICOS stations: BE-Bra - FR-Fon. Site-level mean diurnal cycles of correction-induced differences in net radiation (ΔNETRAD = NETRAD_corr − NETRAD). For each site and correction (slope, topographic shadowing, tower disturbance, and field-of-view/footprint effects), half-hourly means were calculated across 2024. Shaded bands indicate the standard error of the mean. Each panel uses an independent y-axis scale to emphasize intra-site dynamics.

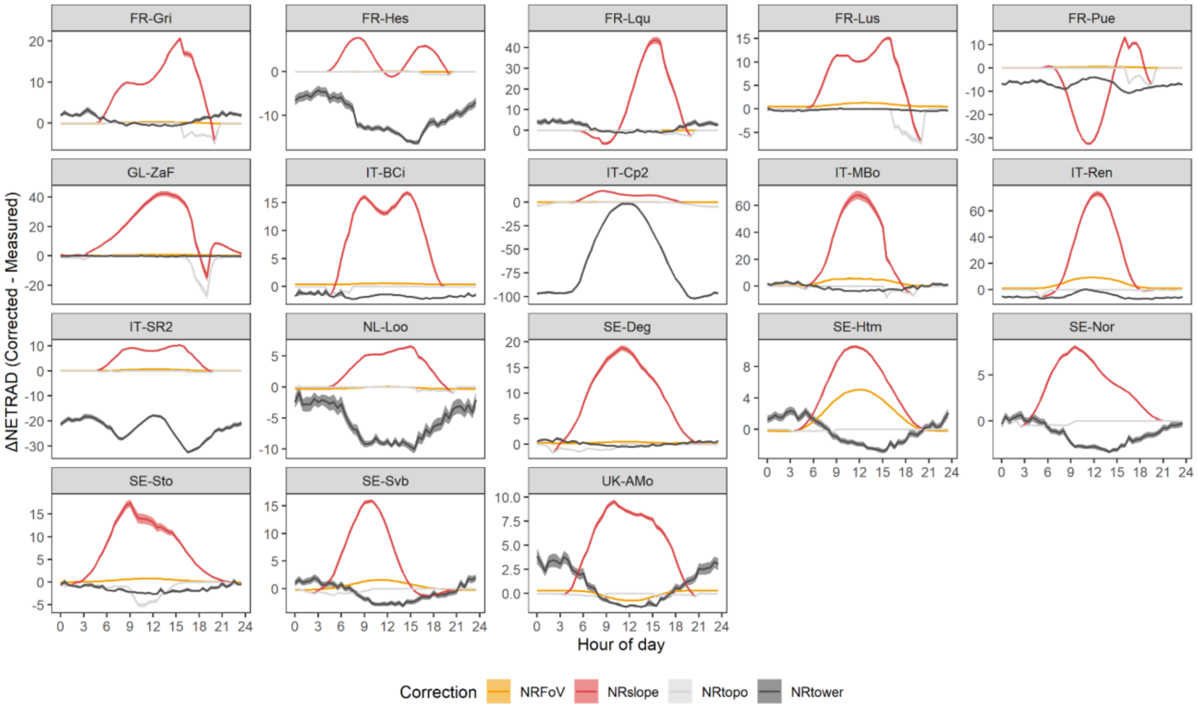

FIGURE S9 b | ICOS stations: FR-Gri - UK-AMo. Site-level mean diurnal cycles of correction-induced differences in net radiation. See the caption of figure S9 a (above).

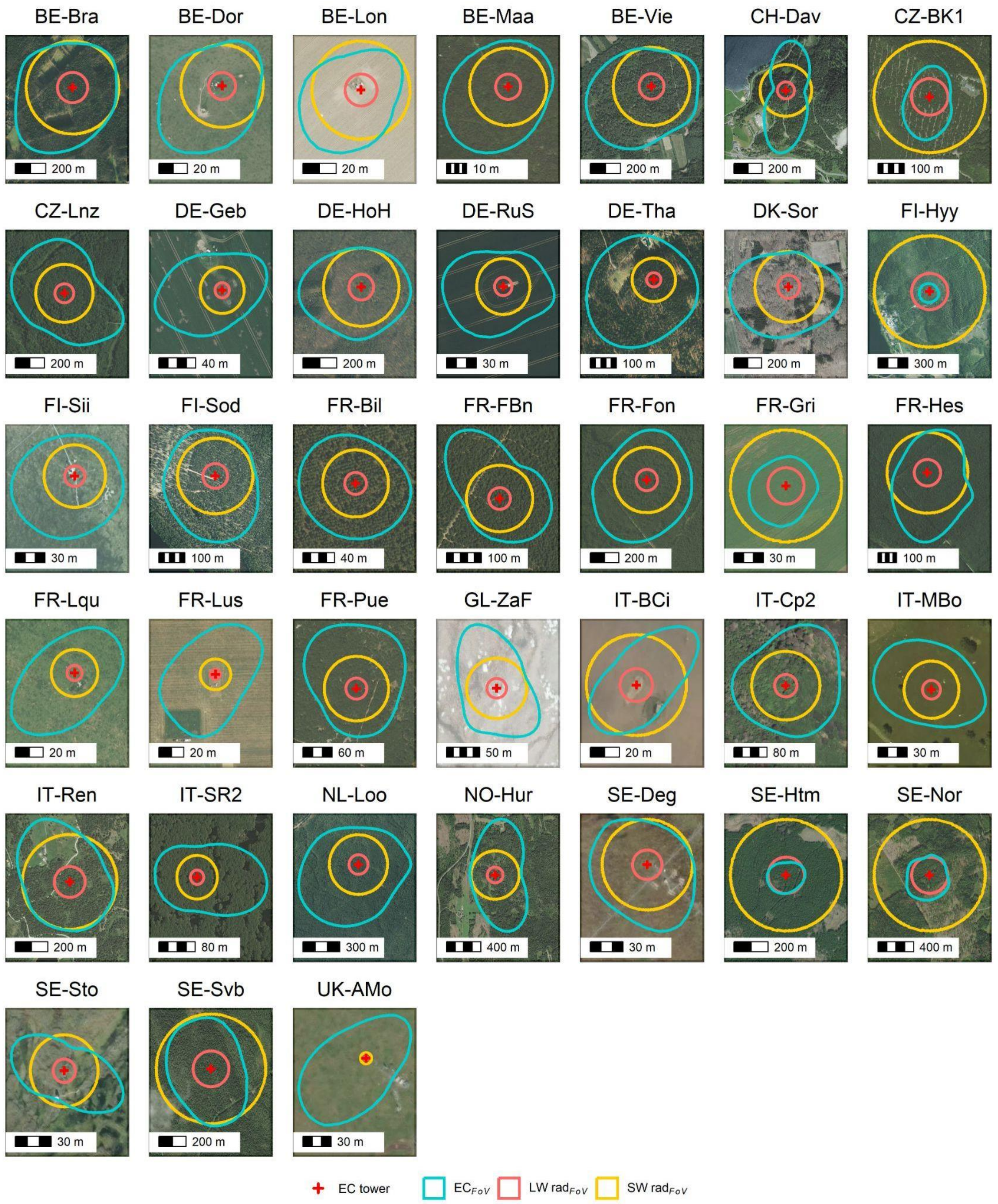

FIGURE S10 | Aerial maps of ICOS stations comparing the EC flux footprint climatology (80% cumulative contribution EC_{FoV}), with the surface projection of the pyrgeometers ($LWrad_{FoV}$) and pyranometer ($SWrad_{FoV}$) field of view.

EBC differences across ecosystem types

Pairwise statistical comparisons of EBC across plant functional types (PFTs) reveal both robust and formulation-dependent patterns (Tables S4 a-b). When all EBC formulations (AE1 through AE6(u*)) are pooled together (Table S4_a), several ecosystem pairs exhibit statistically significant differences ($p < 0.05$), indicating that vegetation type influences energy balance closure quality. However, when analysis is restricted to the most comprehensive AE formulation and rigorous quality filtering on turbulent energy fluxes (AE6(u*), Table S4_b), most of these differences become statistically non-significant, suggesting that methodological choices (energy accounting completeness and quality control stringency) exert stronger influence on EBC than ecosystem type in itself.

TABLE S4 a | EBC comparison among PFT, all EBC formulations included (AE1-AE6(u*)). Wilcoxon rank sum test: pairwise comparisons between group levels with corrections for multiple testing. Statistically significant difference if $p < 0.05$ (values in bold).

	WET	SHR|SAV	GRA	CRO	MF	ENF
SHR|SAV	0.231	-	-	-	-	-
GRA	**0.012**	0.891	-	-	-	-
CRO	**0.000**	**0.000**	**0.000**	-	-	-
MF	0.196	0.756	0.846	**0.022**	-	-
ENF	**0.001**	0.196	**0.014**	**0.001**	0.464	-
DBF|EBF	**0.005**	0.711	0.711	**0.000**	0.758	0.061

TABLE S4 b | EBC comparison among PFT, only AE6(u*) EBC formulation. Wilcoxon rank sum test: pairwise comparisons between group levels with corrections for multiple testing. Statistically significant difference if $p < 0.05$ (values in bold).

	WET	SHR|SAV	GRA	CRO	MF	ENF
SHR|SAV	0.476	-	-	-	-	-
GRA	0.315	0.822	-	-	-	-
CRO	0.053	0.304	0.053	-	-	-
MF	0.476	0.670	0.534	0.711	-	-
ENF	0.118	0.476	0.053	0.476	0.927	-
DBF|EBF	0.053	0.476	0.118	0.315	0.927	0.670

EBC network-related differences among ecosystems

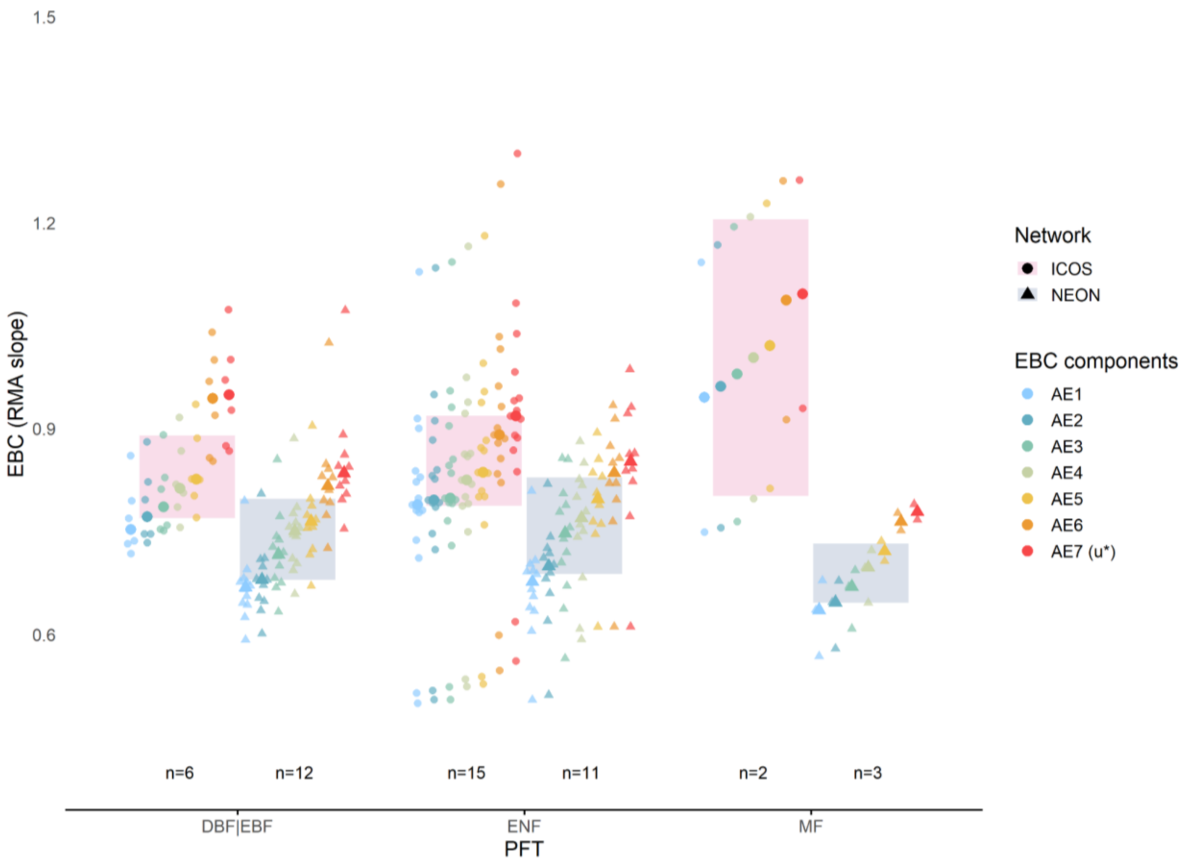

FIGURE S11 a | Distribution of EBC (RMA slopes at half-hourly time scale) for each *forest ecosystem* plant functional type (PFT), separated by network. The number of sites per PFT and network is indicated below each group (n). PFTs are: deciduous broadleaf forests and evergreen broadleaf forests (DBF|EBF), evergreen needleleaf forests (ENF), and mixed forests (MF). Individual sites are shown as jittered points, with the color indicating the AE-TE coupling and the shape indicating the network. Medians for each PFT × AE/TE × network combination are shown as larger points. Boxplots indicate the PFT/network overall EBC distribution.

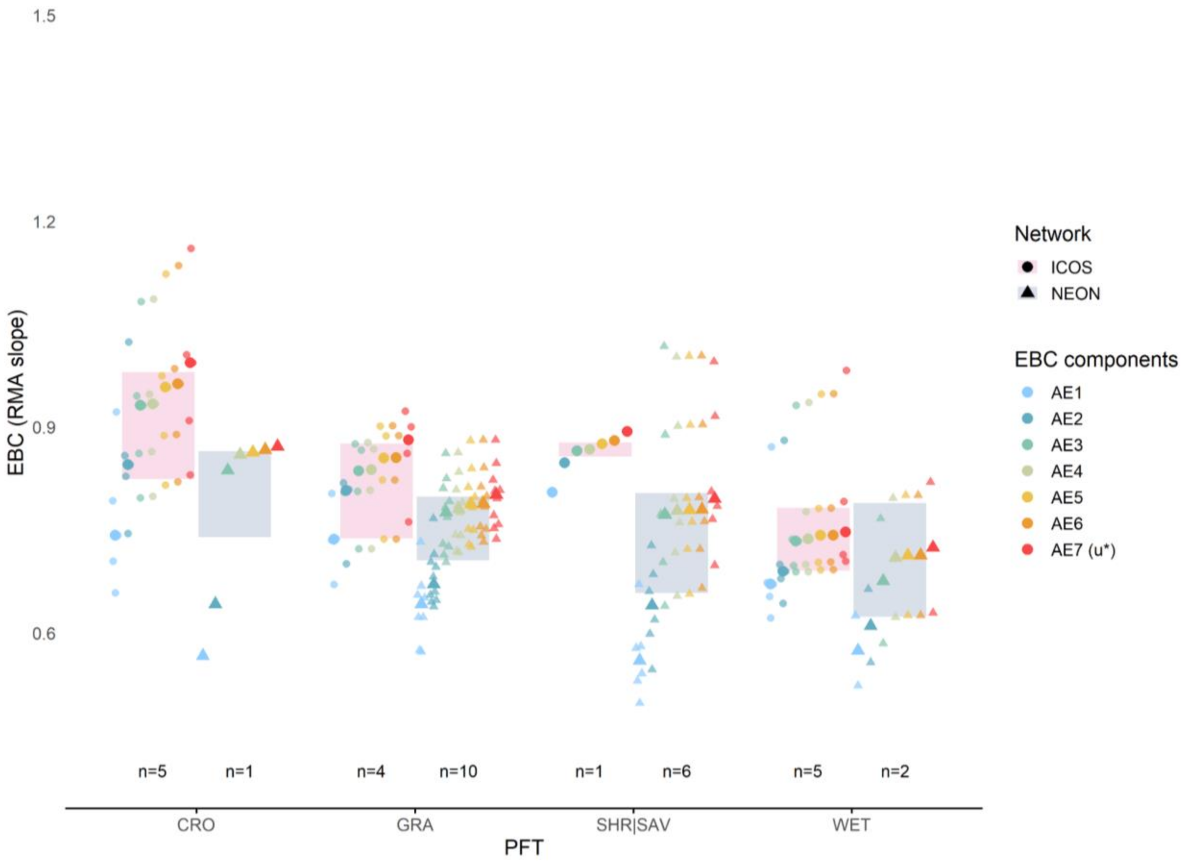

FIGURE S11 b | Distribution of EBC (RMA slopes at half-hourly time scale) for each non-*forest ecosystem* plant functional type (PFT), separated by network. The number of sites per PFT and network is indicated below each group (n). PFTs are: croplands (CRO), grasslands (GRA), savannas/shrublands (SHR|SAV), and wetlands (WET). Individual sites are shown as jittered points, with the color indicating the AE-TE coupling and the shape indicating the network. Medians for each PFT × AE/TE × network combination are shown as larger points. Boxplots indicate the PFT/network overall EBC distribution.

References

Kustas, W., Prueger, J., Hatfield, J., Ramalingam, K., Hipps, L., 2000. Variability in soil heat flux from a mesquite dune site. Agricultural and Forest Meteorology, 103, 3, 2000, 249-264. https://doi.org/10.1016/S0168-1923(00)00131-3.

Lasslop, G., Reichstein, M., Papale, D., Richardson, A.D., Arneth, A., Barr, A.G., Stoy, P.C., Wohlfahrt, G., 2009. Separation of net ecosystem exchange into assimilation and respiration using a light response curve approach: critical issues and global evaluation. Global Change Biology 16, 187–208. doi:10.1111/j.1365-2486.2009.02041.x

Papale, D., Reichstein, M., Aubinet, M., Canfora, E., Bernhofer, C., Kutsch, W., Longdoz, B., Rambal, S., Valentini, R., Vesala, T., Yakir, D., 2006. Towards a standardized processing of Net Ecosystem Exchange measured with eddy covariance technique: algorithms and uncertainty estimation. Biogeosciences 3, 571–583. doi:10.5194/bg-3-571-2006

Shao, C., Chen, J., Li, L., Xu, W., Chen, S., Gwen, T., Xu, J., Zhang, W., 2008. Spatial variability in soil heat flux at three Inner Mongolia steppe ecosystems. Agricultural and Forest Meteorology, 148, 10, 1433-1443. https://doi.org/10.1016/j.agrformet.2008.04.008.

Reichstein, M., Falge, E., Baldocchi, D.D., Papale, D., Aubinet, M., Berbigier, P., Bernhofer, C., Buchmann, N., Gilmanov, T., Granier, A., Grunwald, T., Havrankova, K., Ilvesniemi, H., Janous, D., Knohl, A., Laurila, T., Lohila, A., Loustau, D., Matteucci, G., Meyers, T., Miglietta, F., Ourcival, J.-M., Pumpanen, J., Rambal, S., Rotenberg, E., Sanz, M., Tenhunen, J., Seufert, G., Vaccari, F., Vesala, T., Yakir, D., Valentini, R., 2005. On the separation of net ecosystem exchange into assimilation and ecosystem respiration: review and improved algorithm. Global Change Biology 11, 1424–1439. doi:10.1111/j.1365-2486.2005.001002.x
